# Reduced pro-inflammatory dendritic cell phenotypes are a potential indicator of successful peanut oral immunotherapy

**DOI:** 10.1371/journal.pone.0264674

**Published:** 2022-05-26

**Authors:** Sara Anvari, Levi B. Watkin, Charles G. Minard, Kimberly Schuster, Oluwatomi Hassan, Aikaterini Anagnostou, Jordan S. Orange, David B. Corry, Carla M. Davis

**Affiliations:** 1 Department of Pediatrics, Section of Immunology, Allergy and Retrovirology, Baylor College of Medicine, Texas Children’s Hospital, Houston, Texas, United States of America; 2 William T. Shearer Center for Human Immunobiology, Texas Children’s Hospital, Houston, Texas, United States of America; 3 Dan L. Duncan Institute for Clinical and Translational Research, Baylor College of Medicine, Houston, Texas, United States of America; 4 Department of Pediatrics, Vagelos College of Physicians and Surgeons Columbia University, New York, New York, United States of America; 5 Department of Medicine Section of Immunology, Allergy and Rheumatology and Biology of Inflammation Center, Baylor College of Medicine, Houston, Texas, United States of America; 6 Michael E. DeBakey VA Medical Center for Translational Research in Inflammatory Diseases, Baylor College of Medicine, Houston, Texas, United States of America; National Cancer Institute, UNITED STATES

## Abstract

Dendritic cells are important mediators in the early presentation of antigen and regulation of the differentiation of T cells. Peanut oral immunotherapy (POIT) results in desensitization in most peanut allergic individuals (responders), but not in others due to allergic reactions (non-responders). Delineation of early immunologic changes contributing to desensitization would help clarify the POIT mechanism of action. We analyzed dendritic cells in 15 pediatric subjects (5–12 years) undergoing a phase 1 single-center POIT study. We examined dendritic cells at baseline, 6-, 12-, 18- and 24-weeks after initiation of POIT and responders of therapy were compared to non-responders and healthy controls. The distribution frequency of myeloid DCs (mDCs) and plasmacytoid DCs (pDCs) from peripheral blood samples were measured *in vitro*. A general linear mixed model was used, and included fixed effects for cohort (responder, non-responder, or healthy control), time (0-, 6-, 12-, 18-, and 24-weeks), and the cohort-time interaction term. P-values were adjusted for multiple hypothesis testing using Tukey’s method. We observed that POIT responders had reduced TNFa producing myeloid dendritic cells (mDCs) compared to non-responders. Additionally, non-responders had increased OX40L expressing mDCs at 18-weeks compared to responders. In conclusion, our findings suggest that a reduced pro-inflammatory phenotype in DCs could potentially serve as a predictor of early outcome and success of POIT desensitization.

## Introduction

Peanut oral immunotherapy (POIT) has been shown to help circumvent the morbidity of accidental food exposures [1]. The mechanisms of POIT have yet to be comprehensively elucidated, and delineation of early immunologic changes contributing to the development of peanut desensitization without adverse side effects would help clarify optimal patients for the therapy.

Studies suggest that immunotherapy induces antigen-specific T-regulatory cells (Tregs), which attenuate allergen-driven Th2 cytokine responses [2, 3]. Antigen specific T-cells are induced by priming naïve T-cells by dendritic cells (DCs) that shape adaptive immune responses, bridging innate and adaptive immunity. DCs are phenotypically and functionally unique and guide T-cell differentiation, making them important in immunogenic and tolerogenic responses. Different DC populations provide immune context to T-cells by co-stimulatory markers expressed or cytokines secreted [4]. Understanding DCs involved in POIT can help define the immune context of allergy-associated antigens. For this work, we investigated myeloid DCs (mDCs) and plasmacytoid DCs (pDCs), both known to have antigen-presenting capabilities. We examined dendritic cell co-stimulatory markers and dendritic cell cytokines during the first 6-months of a 3-year high-dose open-label POIT treatment, to identify differences between POIT responders and non-responders and identify an early immune profile that could potentially predict successful POIT response.

## Materials and methods

### Study design

The open label study was approved by the Baylor College of Medicine Institutional Review Board (H-26819). All patients and parents provided written and informed assent/consent. This trial was registered under ClinicalTrials.org (NCT 02203799). Fifteen subjects between the ages of 5–12 years with IgE-mediated peanut allergy were recruited for a 3-year high-dose open label POIT pilot study. Patient baseline demographic and clinical characteristics are summarized in Table 1. All patients underwent a baseline double-blind placebo-controlled food challenge to confirm their peanut allergy. Patients received daily doses of peanut protein and underwent a dose escalation of peanut protein every 2 weeks in the clinical research unit and were observed for 2 hours following a dose challenge. Patients were advised to take a daily dose at home around the same time, followed by a 2-hour observation. Patients returned every 2 weeks for a dose-escalation until they achieved the maintenance dose of 3900mg peanut protein. The duration to escalate to the maintenance dose was approximately 1 year (Mean = 59 weeks, Range = 44–78 weeks) and patients remained on maintenance for an additional 2 years. DC populations were analyzed during the first 6 months of build-up to the POIT maintenance dose. “Responders” of POIT (n = 11) were defined by successful escalation to the maintenance dose and had no adverse events requiring epinephrine during POIT build-up. “Non-responders” of POIT (n = 4) had >/ = 1 adverse events requiring epinephrine, had more than 1 dose drop in the peanut dose level during build-up, and were eventually unable to achieve the maintenance dose. Healthy non-food allergic controls (n = 5) were compared to responders and non-responders to assess baseline differences in the expression of DC costimulatory markers and dendritic cell cytokines. Healthy non-food allergic subjects did not receive treatment with POIT. Blood samples were collected at baseline (prior to each DBPCFC) and every 6-weeks during the build-up phase, until the maintenance dose was achieved.

**Table 1:** Baseline patient demographics and clinical characteristics.

Characteristics	RESPONDERS (N = 11)	NON-RESPONDERS (N = 4)	P-value*
**Age (y), median (range)**	**9 (5.2–12.6)**	**8.7 (5.3–9.8)**	**0.57**
**Sex**			
Female	**6/11 (55%)**	**1/4 (25%)**	**0.57**
**Baseline Peanut IgE (kU/L), median (range)**	**51 (16–571)**	**413 (32–784)**	**0.18**
**Baseline Peanut Skin Test Wheal (mm), median (range)**	**14 (7–27)**	**24 (18–36)**	**0.07**
**Baseline Food Challenge Reaction Dose (mg), median (range)**	**75 (15–250)**	**64.5 (15–1000)**	**0.88**
**Other allergic diseases**			
Allergic Rhinitis	**8/11 (79%)**	**1/3 (33%)**	**0.51**
Atopic Dermatitis	**4/11 (36%)**	**0/3 (0%)**	**0.51**
Asthma	**2/11 (18%)**	**1/3 (33%)**	**1.00**
Other Food Allergies	**7/11 (64%)**	**2/3 (67%)**	**1.00**

*P-values comparing responders versus non-responders using Fisher’s exact test or Wilcoxon rank sum test.

### Inclusion & exclusion criteria

Patients were required to have a peanut specific IgE value >7kU/L and positive peanut specific skin prick test (SPT) (wheal diameter size 3mm greater than the negative saline control). All participants underwent a double-blind placebo-controlled food challenge (DBPCFC) to peanut prior to enrollment. Once enrolled, each patient underwent peanut oral immunotherapy (POIT) build-up every 2 weeks, with a final goal maintenance dose of 3900mg of peanut protein (approx. 20 peanuts), which was achieved within 1 year of starting therapy. Patients underwent DBPCFC up to 6000mg peanut protein (cumulative dose 26,225mg peanut protein) within 1 month of achieving the maintenance dose. “Responders” successfully achieved the maintenance dose and had no adverse events requiring epinephrine during POIT build-up. “Non-responders” had >/ = 1 adverse events requiring epinephrine, had more than 1 dose drop in the dose level during build-up, and were unable to achieve the maintenance dose. The maintenance dose for each non-responder was maintained below 3900mg and adjusted to prevent any allergic reactions. Blood samples were collected at baseline (prior to each DBPCFC) and then every 6 weeks thereafter, until the maintenance dose was achieved.

Patients were excluded from study enrollment if they met any of the following exclusion criteria: History of severe anaphylaxis to peanut (Grade 3 or higher per WAO guidelines); participation in another study using an investigational new drug; participation in any interventional study for the treatment of food allergy in the past 12 months or while participating in this study; poor control of persistent atopic dermatitis; a diagnosis of asthma; inability to discontinue antihistamines for skin testing and oral food challenges; pregnant females; chronic medical conditions requiring frequent use of oral steroids, chronic psychiatric illness or history of substance abuse; active eosinophilic esophagitis requiring therapy 12 months prior to study enrollment; subjects with known wheat or oat allergy; subjects on the build-up phase of environmental allergen immunotherapy injection; and/or subject living more than 175 miles away from Texas Children’s Hospital.

### Preparation of cryopreserved peripheral blood mononuclear cells (PBMCs)

Blood was collected from each subject at baseline and every 6-weeks during their POIT build-up phase. Peripheral blood mononuclear cells (PBMCs) were isolated from blood samples by density gradient centrifugation over Ficoll-Paque, and cryopreserved in 10% dimethyl sulfoxide in fetal bovine serum and stored in liquid nitrogen.

### Cell culture

Cryopreserved PBMCs were thawed and then cultured in media (RPMI-1640, glutamine, 10% fetal bovine serum, 1% penicillin/streptomycin) and incubated in 96-well plates with media alone, lipopolysaccharide (LPS) (1ng/mL) from *Escherichia coli* 0111:B4 (Sigma) or crude peanut extract (CPE) (200ug/mL) for 24 hours at 37C°, 5% CO_2_. LPS is a negative stimulator of type 2 allergic inflammation, therefore we used LPS as a control to assess perturbations in the non-type 2 allergic response between peanut allergic and non-peanut allergic subjects. CPE is a stimulant promoting Type 2 allergic inflammation in peanut allergic subjects, thus it would not promote a type 2 response in non-peanut allergic subjects. CPE was used to assess the peanut antigen effect on DCs in the peanut allergic subjects undergoing POIT treatment, since CPE clinically promotes Type 2 inflammation in peanut allergic subjects. To capture intracellular cytokines, brefeldin A (BD Biosciences, 1uL/mL) and monensin (BD Biosciences 0.7uL/mL) were added to each culture at the beginning of incubation.

### Cell staining

Cell cultures were collected after 24 hours of incubation. Cells were stained with a viability dye/Ghost V510 (Tonbo Biosciences, San Diego, CA) and incubated at room temperature for 15 minutes protected from light. Cell were stained with cell surface antibody for 30 minutes at room temperature and protected from light, followed by washing with wash buffer (PBS+0.25% BSA) twice. Surface marker antibody cocktail was made using 2.5uL of each antibody for every 1e6 cells: anti-CD123 (BV605, clone 6H6, BioLegend), anti-HLA-DR (BV786, clone G46-6, BD Horizon), anti-CD11c (PE/Cy7, clone B-ly6, BD Pharmingen), anti-CD14 (APC/Cy7, clone M5E2, BioLegend), anti-CD252 (OX40L) (PE, clone 11C3.1, BioLegend), anti-CD19 (AF700, clone HIB19, BioLegend), anti-CD56 (BV510, clone NCAM16.2, BD Horizon) and anti-CD3 (BV510, clone OKT3, BioLegend). Cells were incubated with surface cocktail for 30 minutes protected from light at room temperature. For staining intracellular cytokines, cells were washed twice with fixation/permeabilization buffer (BD Biosciences). Permeabilized cells were stained with an intracellular marker antibody cocktail of the following antibodies (5uL of each antibody for every 1e6 cells): anti-IL4 (BV711, clone MP4-25D2, BD Horizon), anti-IL6 (FITC, clone MQ2-13A5, BioLegend), anti-IL-10 (PE-CF594, clone JES3-19F1, BD Horizon), and anti-TNFa (BV421, clone Mab11, BioLegend). Cells were incubated with intracellular cocktail for 30 minutes protected from light at room temperature. Cells were washed once with permeabilization buffer and once with wash buffer. In order to standardize samples across time, all cryopreserved POIT samples were run in separate batches of 3 analyzing all timepoints using the same techniques.

### Flow cytometric analysis

**Fig 1:**
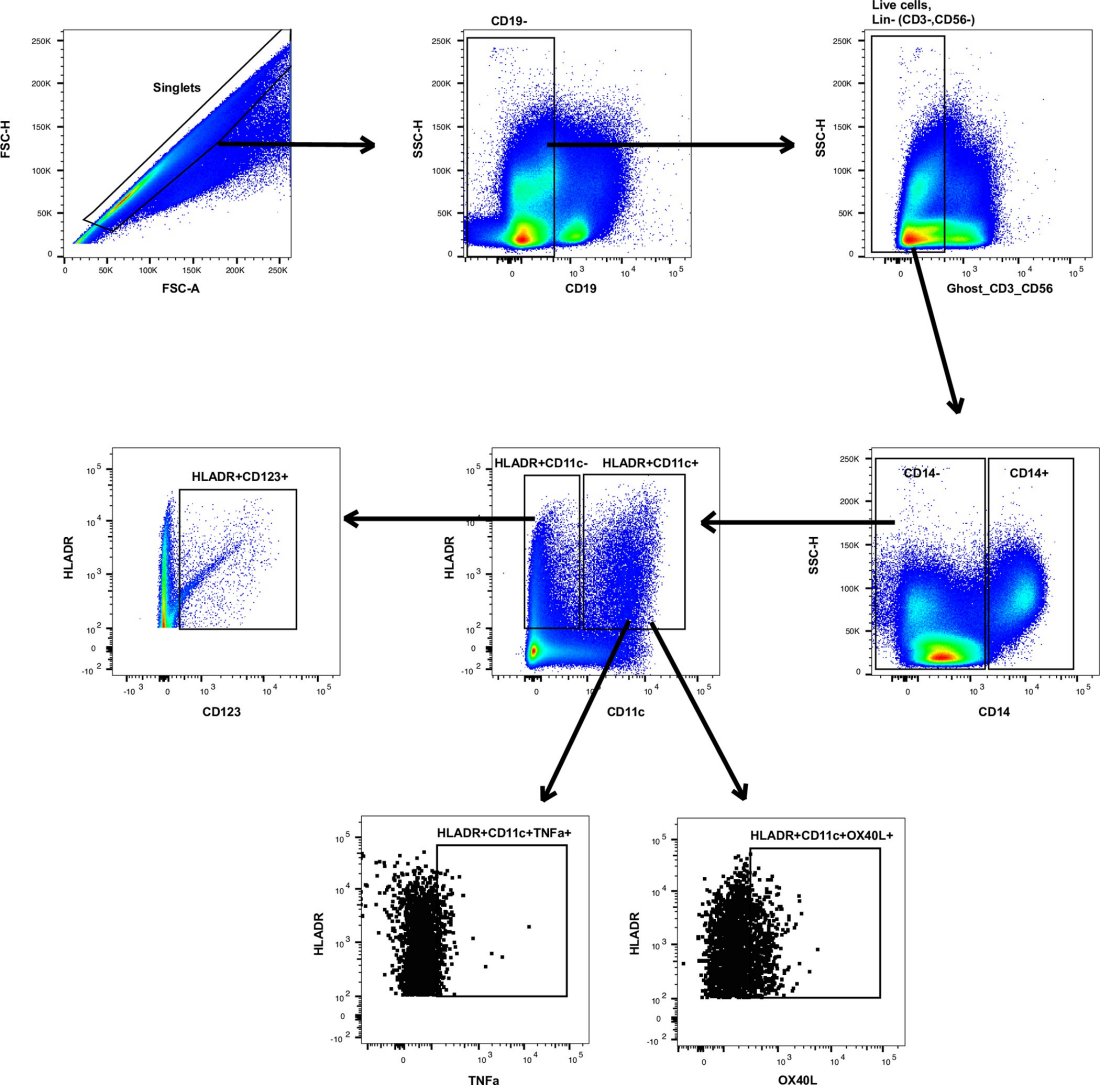
Gating strategy to identify dendritic cell subpopulations from PBMCs. For DC phenotyping, B-cell negative (CD19-), Live positive, and Lineage negative (CD3-CD56-) populations were selected. From the CD19-Live+CD3-CD56- parent population, monocytes were identified by CD14+ expression. From the non-monocyte (CD14-) parent gate, myeloid DCs (mDC) were defined by expression of HLADR+CD11c+. From the mDC parent gate, OX40L and TNFa were identified. From the CD14-HLADR+CD11c- parent gate, plasmacytoid DCs (pDCs) were defined by the expression of HLADR+CD123+.

**Fig 2:**
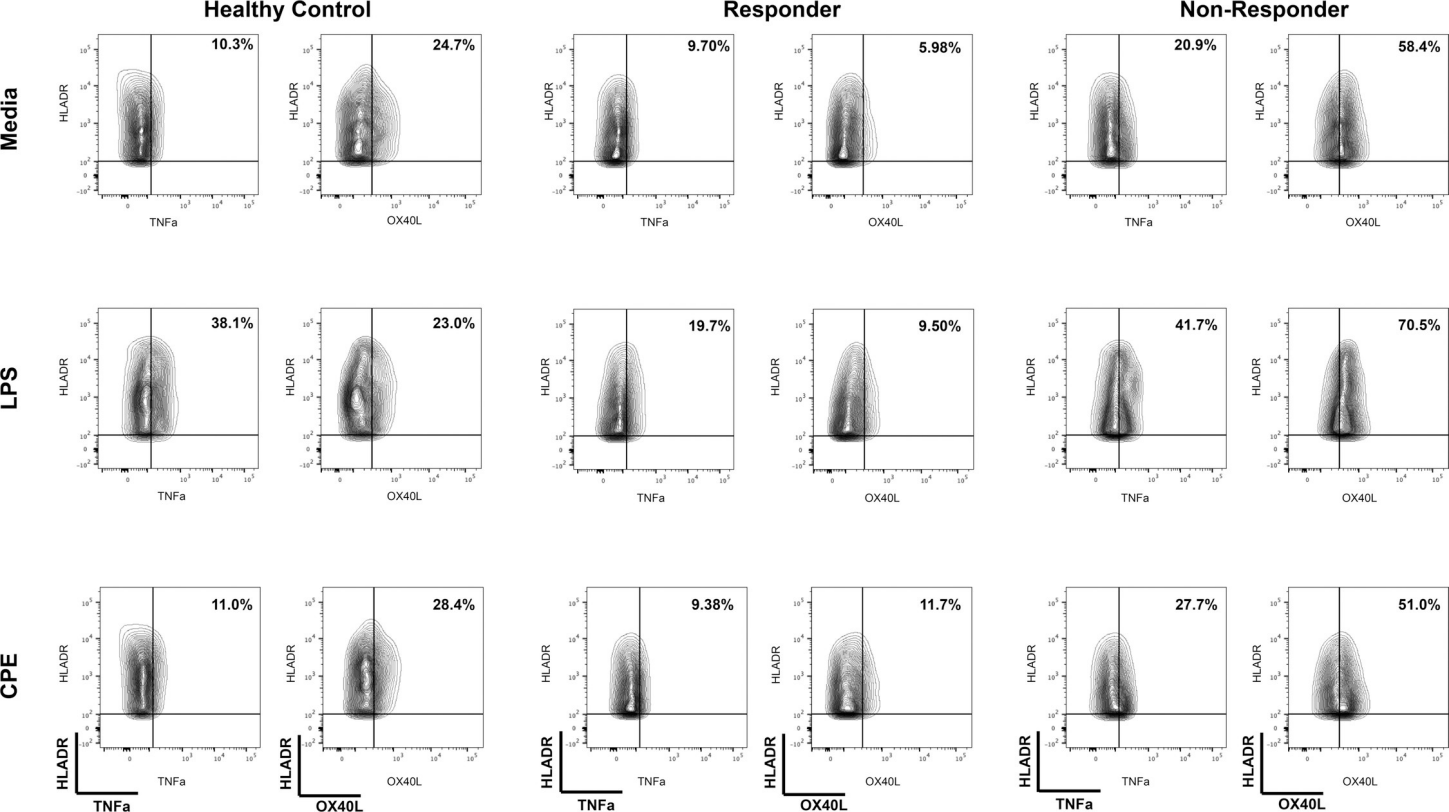
Gating strategy to identify OX40L and TNFa expression on myeloid dendritic cell (mDC) following stimulation with media, lipopolysaccharide (LPS), or crude peanut extract (CPE). (a) Baseline healthy control non-peanut allergic, (b) responder of peanut oral immunotherapy, and (c) non-responder of peanut oral immune therapy. Percentages of each population are based on the parent gate (CD14-HLADR+CD11c+ mDCs).

All samples were run on an 18-parameter flow cytometer (LSRII, BD Immunocytometry Systems^®^, San Jose, CA), and analyses were completed using FlowJo Version 10.5.0 software (FlowJo^®^ LLC). Fig 1 outlines the flow cytometric gating strategy to identify dendritic cell subpopulations from PBMCs. For DC phenotyping, single cell events were identified by the parameters FSC-H and FSC-A. To eliminate B-cells, cells expressing CD19+ were excluded and gated out. Live and lineage negative populations were selected and defined as (Live+ CD3- CD56-). From the CD19-Live+CD3-CD56-parent population, monocytes (CD14+) were identified and excluded. From the non-monocyte (CD14-) parent population, cells were then evaluated for the expression of HLA-DR and CD11c. Myeloid DCs (mDC) were defined as CD14- HLA-DR+ CD11c+ From the mDC parent gate, OX40L and TNFa were separately gated. From the CD14- HLA-DR+ CD11c- parent gate, plasmacytoid DCs (pDC) were identified by their expression of CD14- HLADR+ CD11c- CD123+ (Fig 1). To identify the cytokines and surface markers of interest, the gates were based on unstimulated, single stained and unstained controls. The gating strategy to identify OX40L and TNFa expression on myeloid dendritic cell (mDC) following stimulation with media, lipopolysaccharide (LPS), or crude peanut extract (CPE) can be seen in Fig 2.

### Statistical analysis

Sample size was not prospectively planned for this pilot, hypothesis generating study. The sample size for the POIT study was initially estimated to detect a statistically significant difference in the proportion of T cells producing interferon-gamma (IFN gamma), at the 1-year time point compared with baseline using a paired t-test. A difference in proportions of 0.60 units (.2 at baseline and .8 at 1 year) would be clinically important. Assuming a common SD = 0.6 at each time point, correlation between repeated measures is 0.50 and alpha = 0.05, a sample size of 10 subjects would be required to detect this difference with 80% power. Therefore, assuming 50% attrition, we enrolled a total of 15 subjects. A general linear mixed model was used, and included fixed effects for cohort (responder, non-responder, or healthy control), time (0, 6, 12, 18, and 24 weeks), and the cohort-time interaction term. The matrix of correlated residuals assumed a first-order auto-regressive structure. The model was used to estimate mean frequency (95% CI) and compare all possible pairwise comparisons. P-values were adjusted for multiple hypothesis testing using Tukey’s method. A separate model was fit for each cell type-stimulant combination. Statistical significance was assessed at the 0.05 level.

## Results and discussion

**Fig 3:**
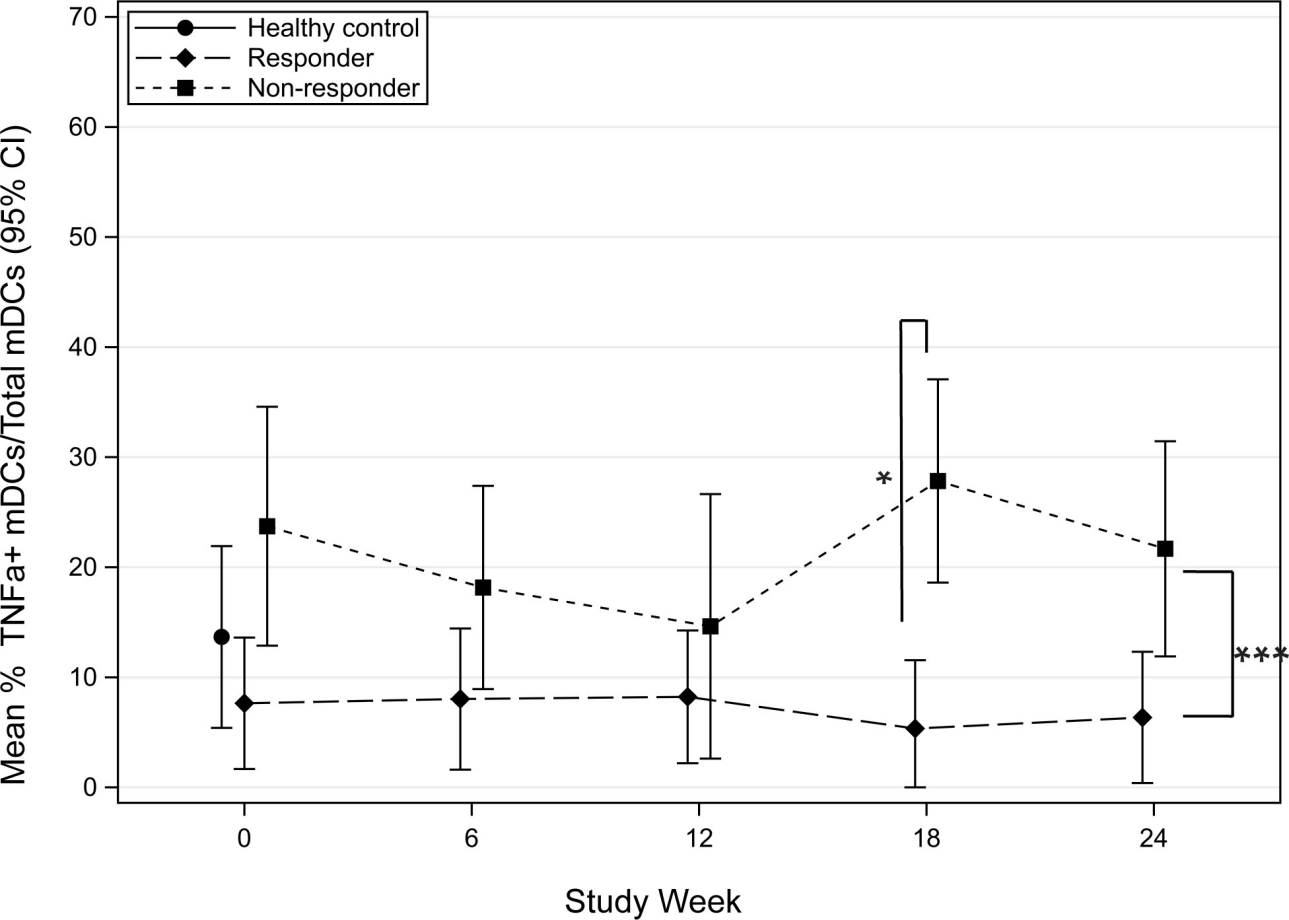
Mean percent frequency (95% CI) of TNFa-producing myeloid dendritic cells (mDCs) following LPS stimulation in POIT responders and non-responders in the first 24-weeks.

**Fig 4:**
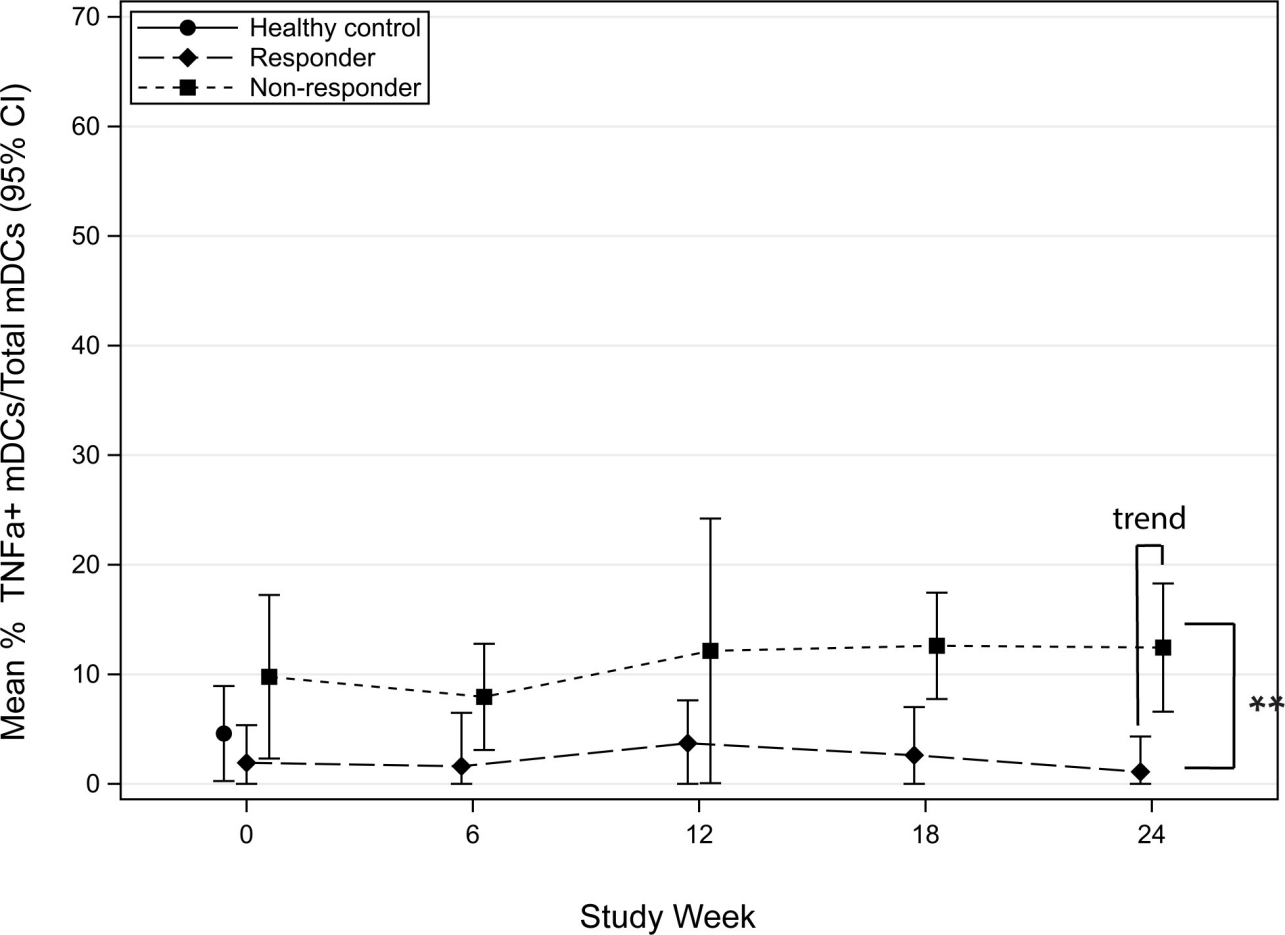
Mean percent frequency (95% CI) of TNFa-producing myeloid dendritic cells (mDCs) following CPE stimulation in POIT responders and non-responders in the first 24-weeks.

Tumor necrosis factor alpha (TNFa), a pleotropic cytokine, has both pro-inflammatory and anti-inflammatory effects [5]. The biological function of TNFa is dependent on the receptor it stimulates. Through the activation of TNFR1, TNFa can stimulate an allergic inflammatory response, whereas stimulation through TNFR2 has been shown to promote immune tolerance [5, 6]. To investigate the early effects of POIT on TNFa, we compared the mean frequency (95% CI) of TNFa-producing mDCs before and during the first 6-months of POIT build-up in responders and non-responders. When stimulated with lipopolysaccharide (LPS), TNFa-producing mDCs, were significantly increased in POIT non-responders compared to responders at 18-weeks of therapy (p = 0.017) (Fig 3). When stimulated with CPE, a statistical trend in TNFa-producing mDCs was observed between non-responders and responders at 24-weeks of therapy, where non-responders had increased TNFa compared to responders (p = 0.075) (Fig 4). On average, TNFa-producing mDCs were 8.8 units greater in non-responders than responders across all time-points (95% CI: 4.8, 12.8; P = 0.0007) following LPS stimulation (Fig 3) and 14.1 units greater in non-responders compared to responders (95% CI: 4.4, 23.8; P = 0.010) following CPE stimulation (Fig 4). Overall, non-responders had more TNFa-producing mDCs compared to responders.

**Fig 5:**
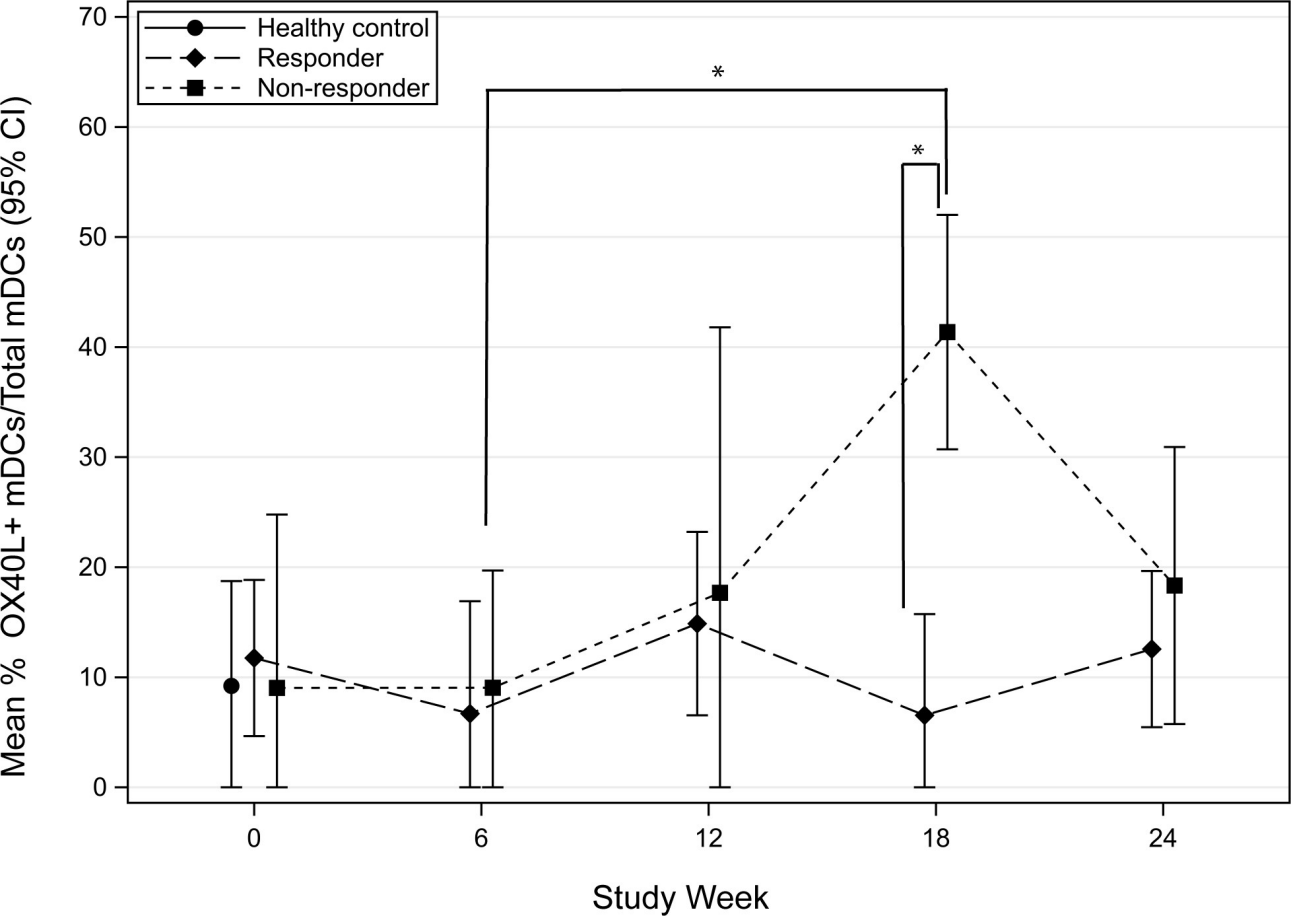
Mean percent frequency (95% CI) of OX40L myeloid dendritic cells (mDCs) following LPS stimulation in the 1^st^ 24-weeks in POIT responders and non-responders.

**Fig 6:**
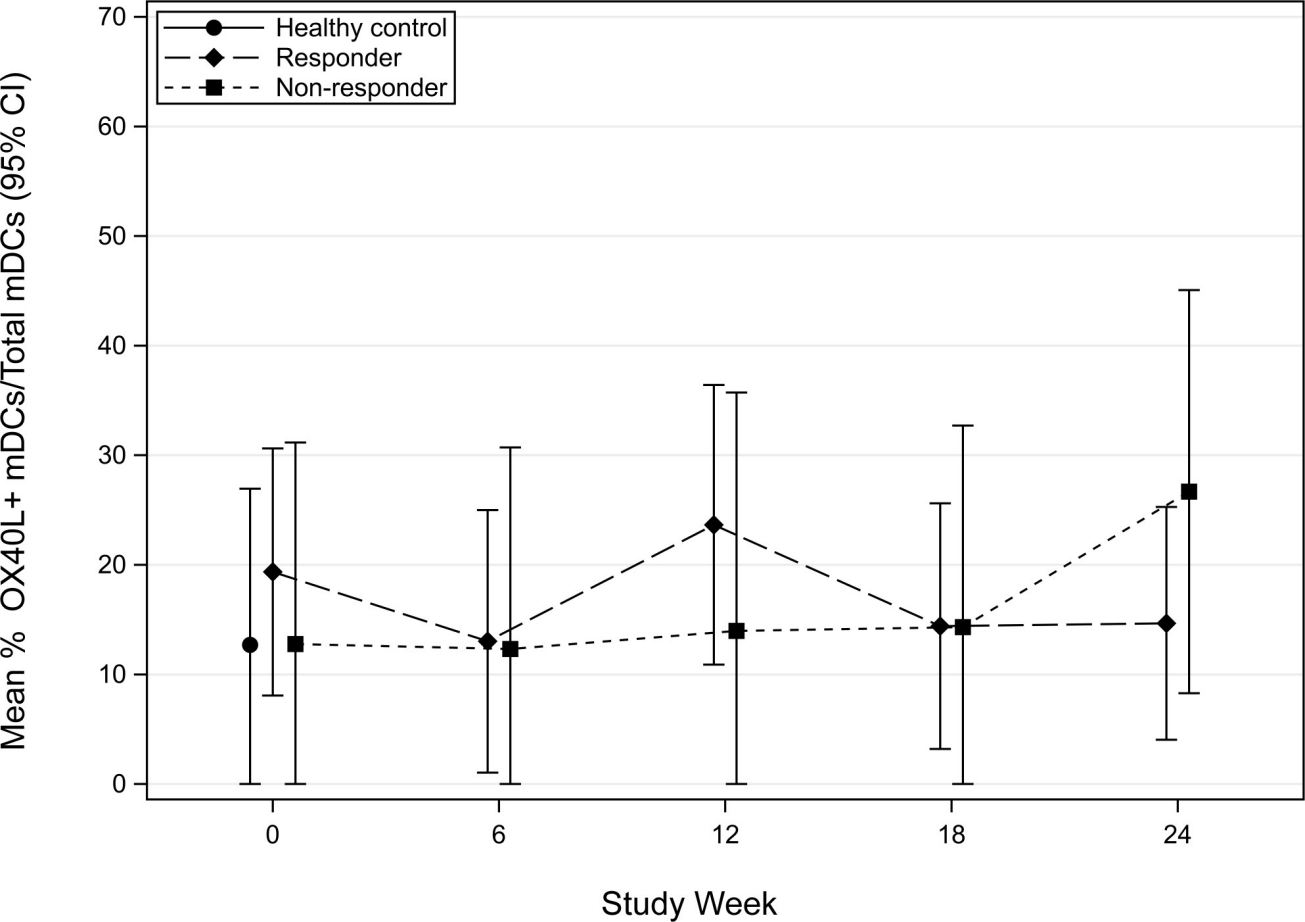
Mean percent frequency (95% CI) of OX40L myeloid dendritic cells (mDCs) following CPE stimulation in the 1^st^ 24-weeks in POIT responders and non-responders.

OX40L (CD252) is a surface glycoprotein found on professional antigen-presenting cells (APCs), such as mDCs. It is a member of the TNFR/TNF superfamily that is important in directing T-helper type 2 (Th2) cell polarization [7]. To investigate the early effects of POIT on OX40L, we compared the mean frequency of OX40L expression on mDCs in responders and non-responders. A significant transient increase in OX40L expression on mDCs was observed in non-responders compared to responders at 18-weeks of therapy (p = 0.001) (Fig 5). Additionally, a significant increase in OX40L expression was also noted to occur in non-responders between 6-weeks and 18-weeks of POIT (p = 0.011) (Fig 5). No significant findings were seen in OX40L expressing mDCs following CPE stimulation (Fig 6).

No significant differences were observed between baseline healthy controls compared to the responders and non-responders for TNFa and OX40L. Additionally, no significant differences were seen in the production of IL-4, IL-6 or IL-10 from mDCs (S1–S6 Figs), no differences were seen in production of IL-4, IL-6, IL-10, and TNFa from pDCs (S7–S14 Figs) and no significant differences were observed in total mDCs in PBMCs or total pDCs in PBMCs between responders, non-responders and healthy controls (S15–S18 Figs).

Suppression of Th2-driven allergic inflammation and type-I hypersensitivity has been associated with successful desensitization to allergens. Specifically this has been observed by an increase in IL-10 producing CD4+ T-cells and reduced Th2 cytokines during POIT [8]. Limited studies have explored early changes in upstream innate mediators of the Th2 response. Our study is novel in that we observed pediatric POIT patients, who achieve POIT desensitization without adverse events, have an overall reduced TNFa production from mDCs and begin to demonstrate early findings of transiently reduced OX40L expression on mDCs during the first 6-months of POIT build-up.

The overall increased frequency of TNFa-producing mDCs in non-responders may contribute to a pro-inflammatory state that allows for persistent sensitization and allergy. Increased TNFa production has been associated with either promotion of inflammation through the activation of TNFR1 or maintenance of immune homeostasis and tolerance through activation of TNFR2 [5, 6]. In our open-label pilot POIT study, we observed a significant increase in TNFa-producing mDCs at 18-weeks of therapy and an overall average increase in TNFa-producing mDCs in non-responders compared to responders. Thus, the upregulation of TNFa in non-responders compared to responders may suggest an additional mechanism for perpetuation of maladaptive allergic mechanisms by signaling through TNFR1. Additional studies with a larger cohort are required to examine if dysregulation of TNF receptors further contributes to this potential mechanism.

Signaling through OX40L can mediate Th2 differentiation and break T-cell tolerance by binding to OX40 on the surface of T-cells [9]. In this study, preliminary findings show that OX40L is increased in non-responders at week-18, suggesting that there is an impairment in mitigating the inflammatory allergic response with POIT. Additional studies to examine OX40L expression are needed in a larger cohort of POIT subjects to determine if this mechanism can explain the type-2 immunity observed in non-responders. A limitation in the analysis and interpretation of the TNFa and OX40L data is the gaps in sample timepoint collection for each patient.

## Conclusion

POIT responders and non-responders were expected to differ in their DC response to CPE, and that DC responses to LPS would be similar in these two cohorts. However, our findings demonstrated DC differences occurring in certain DC populations following LPS stimulation as well as some populations following CPE stimulation between POIT responders and non-responders. Dendritic cells from POIT non-responders demonstrated that their universal ability to respond to different stimuli was hindered broadly and this was not limited to just peanut antigen stimulation, which was the driver of their peanut allergy. Using LPS as a control allowed us to assess perturbations in the non-type 2 allergic response between peanut allergic and non-peanut allergic subjects. If CPE was the only stimulant assessed, then it still would not be understood if the DC effects seen were just peanut specific or due to a broader context. By testing LPS and CPE, we are able to determine a broader inflammatory signature of dendritic cells in the context of non-specific antigens and assess the susceptibility and persistence for Type 2 inflammation.

POIT may promote a shift away from Th2 or Type 2 inflammation and allow for development of adaptive Tregs thus inducing desensitization [8]. Our findings provide evidence that POIT responders show reduced frequency of TNF-producing mDCs compared to non-responders during the first 6-months of POIT build-up and early transient findings of increased frequency of OX40L in non-responders compared to responders. We demonstrate the influence of POIT on DCs to promote desensitization and the promise of potential non-invasive predictors of successful POIT. However, due to the limited nature of the sample collected for each patient, we must exercise caution in overinterpreting the results. To fully understand the role of these TNFa-producing mDCs further studies will address missing timepoints as well as increase patient numbers in both the responder, non-responder, and healthy controls. In conclusion, our findings suggest that a reduced pro-inflammatory DC phenotype could potentially serve as a predictor of early outcome and success of POIT desensitization.

## Supporting information

S1 Fig Mean percentage of IL-4+ myeloid dendritic cells (mDCs) in total mDCs following LPS stimulation. Changes in frequency of IL-4 expressing mDCs following LPS stimulation between responders (diamond) and non-responders (square) of peanut oral immunotherapy during the first 24-weeks of therapy. Healthy controls (circle) were not treated and only assessed at baseline.
(TIF)

S2 Fig Mean percentage of IL-4+ myeloid dendritic cells (mDCs) in total mDCs following CPE stimulation. Changes in frequency of IL-4 expressing mDCs following CPE stimulation between responders (diamond) and non-responders (square) of peanut oral immunotherapy during the first 24-weeks of therapy. Healthy controls (circle) were not treated and only assessed at baseline.
(TIF)

S3 Fig Mean percentage of IL-6+ myeloid dendritic cells (mDCs) in total mDCs following LPS stimulation. Changes in frequency of IL-6 expressing mDCs following LPS stimulation between responders (diamond) and non-responders (square) of peanut oral immunotherapy during the first 24-weeks of therapy. Healthy controls (circle) were not treated and only assessed at baseline.
(TIF)

S4 Fig Mean percentage of IL-6+ myeloid dendritic cells (mDCs) in total mDCs following CPE stimulation. Changes in frequency of IL-6 expressing mDCs following CPE stimulation between responders (diamond) and non-responders (square) of peanut oral immunotherapy during the first 24-weeks of therapy. Healthy controls (circle) were not treated and only assessed at baseline.
(TIF)

S5 Fig Mean percentage of IL-10+ myeloid dendritic cells (mDCs) in total mDCs following LPS stimulation. Changes in frequency of IL-10 expressing mDCs following LPS stimulation between responders (diamond) and non-responders (square) of peanut oral immunotherapy during the first 24-weeks of therapy. Healthy controls (circle) were not treated and only assessed at baseline.
(TIF)

S6 Fig Mean percentage of IL-10+ myeloid dendritic cells (mDCs) in total mDCs following CPE stimulation. Changes in frequency of IL-10 expressing mDCs following CPE stimulation between responders (diamond) and non-responders (square) of peanut oral immunotherapy during the first 24-weeks of therapy. Healthy controls (circle) were not treated and only assessed at baseline.
(TIF)

S7 Fig Mean percentage of IL-4+ plasmacytoid dendritic cells (pDCs) in total pDCs following LPS stimulation. Changes in frequency of IL-4 expressing pDCs following LPS stimulation between responders (diamond) and non-responders (square) of peanut oral immunotherapy during the first 24-weeks of therapy. Healthy controls (circle) were not treated and only assessed at baseline.
(TIF)

S8 Fig Mean percentage of IL-4+ plasmacytoid dendritic cells (pDCs) in total pDCs following CPE stimulation. Changes in frequency of IL-4 expressing pDCs following CPE stimulation between responders (diamond) and non-responders (square) of peanut oral immunotherapy during the first 24-weeks of therapy. Healthy controls (circle) were not treated and only assessed at baseline.
(TIF)

S9 Fig Mean percentage of IL-6+ plasmacytoid dendritic cells (pDCs) in total pDCs following LPS stimulation. Changes in frequency of I6-4 expressing pDCs following LPS stimulation between responders (diamond) and non-responders (square) of peanut oral immunotherapy during the first 24-weeks of therapy. Healthy controls (circle) were not treated and only assessed at baseline.
(TIF)

S10 Fig Mean percentage of IL-6+ plasmacytoid dendritic cells (pDCs) in total pDCs following CPE stimulation. Changes in frequency of IL-6 expressing pDCs following CPE stimulation between responders (diamond) and non-responders (square) of peanut oral immunotherapy during the first 24-weeks of therapy. Healthy controls (circle) were not treated and only assessed at baseline.
(TIF)

S11 Fig Mean percentage of IL-10+ plasmacytoid dendritic cells (pDCs) in total pDCs following LPS stimulation. Changes in frequency of IL-10 expressing pDCs following LPS stimulation between responders (diamond) and non-responders (square) of peanut oral immunotherapy during the first 24-weeks of therapy. Healthy controls (circle) were not treated and only assessed at baseline.
(TIF)

S12 Fig Mean percentage of IL-10+ plasmacytoid dendritic cells (pDCs) in total pDCs following CPE stimulation. Changes in frequency of IL-10 expressing pDCs following CPE stimulation between responders (diamond) and non-responders (square) of peanut oral immunotherapy during the first 24-weeks of therapy. Healthy controls (circle) were not treated and only assessed at baseline.
(TIF)

S13 Fig Mean percentage of TNFa+ plasmacytoid dendritic cells (pDCs) in total pDCs following LPS stimulation. Changes in frequency of TNFa expressing pDCs following LPS stimulation between responders (diamond) and non-responders (square) of peanut oral immunotherapy during the first 24-weeks of therapy. Healthy controls (circle) were not treated and only assessed at baseline.
(TIF)

S14 Fig Mean percentage of TNFa+ plasmacytoid dendritic cells (pDCs) in total pDCs following CPE stimulation. Changes in frequency of TNFa expressing pDCs following CPE stimulation between responders (diamond) and non-responders (square) of peanut oral immunotherapy during the first 24-weeks of therapy. Healthy controls (circle) were not treated and only assessed at baseline.
(TIF)

S15 Fig Mean percentage of total myeloid dendritic cells (mDCs) in total PBMCs following LPS stimulation. Changes in frequency of total mDCs following LPS stimulation between responders (diamond) and non-responders (square) of peanut oral immunotherapy during the first 24-weeks of therapy. Healthy controls (circle) were not treated and only assessed at baseline.
(TIF)

S16 Fig Mean percentage of total myeloid dendritic cells (mDCs) in total PBMCs following CPE stimulation. Changes in frequency of total mDCs following CPE stimulation between responders (diamond) and non-responders (square) of peanut oral immunotherapy during the first 24-weeks of therapy. Healthy controls (circle) were not treated and only assessed at baseline.
(TIF)

S17 Fig Mean percentage of total plasmacytoid dendritic cells (pDCs) in total PBMCs following LPS stimulation. Changes in frequency of total pDCs following LPS stimulation between responders (diamond) and non-responders (square) of peanut oral immunotherapy during the first 24-weeks of therapy. Healthy controls (circle) were not treated and only assessed at baseline.
(TIF)

S18 Fig Mean percentage of total plasmacytoid dendritic cells (pDCs) in total PBMCs following CPE stimulation. Changes in frequency of total pDCs following CPE stimulation between responders (diamond) and non-responders (square) of peanut oral immunotherapy during the first 24-weeks of therapy. Healthy controls (circle) were not treated and only assessed at baseline.
(TIF)

S19 Fig Scattered plot illustrating mean percent frequency (95% CI) of TNFa-producing myeloid dendritic cells (mDCs) in POIT responders and non-responders in the first 24-weeks. (A) Mean percent frequency of TNFa-production mDCs following LPS stimulation; (B) Mean percent frequency of TNFa-production mDCs following CPE stimulation.
(PNG)

S20 Fig Scattered plot illustrating mean percent frequency (95% CI) of OX40L myeloid dendritic cells (mDCs) in the 1^st^ 24-weeks in POIT responders and non-responders. (A) Mean percent frequency (95% CI) of OX40L mDCs following LPS stimulation, (B) Mean percent frequency (95% CI) of OX40L mDCs following CPE stimulation.
(PNG)

S21 Fig Scattered plot illustrating mean percentage of IL-4+ myeloid dendritic cells (mDCs) in total mDCs following LPS stimulation. Changes in frequency of IL-4 expressing mDCs following LPS stimulation between responders (diamond) and non-responders (square) of peanut oral immunotherapy during the first 24-weeks of therapy. Healthy controls (circle) were not treated and only assessed at baseline.
(PNG)

S22 Fig Scattered plot illustrating mean percentage of IL-4+ myeloid dendritic cells (mDCs) in total mDCs following CPE stimulation. Changes in frequency of IL-4 expressing mDCs following CPE stimulation between responders (diamond) and non-responders (square) of peanut oral immunotherapy during the first 24-weeks of therapy. Healthy controls (circle) were not treated and only assessed at baseline.
(PNG)

S23 Fig Scattered plot illustrating mean percentage of IL-6+ myeloid dendritic cells (mDCs) in total mDCs following LPS stimulation. Changes in frequency of IL-6 expressing mDCs following LPS stimulation between responders (diamond) and non-responders (square) of peanut oral immunotherapy during the first 24-weeks of therapy. Healthy controls (circle) were not treated and only assessed at baseline.
(PNG)

S24 Fig Scattered plot illustrating mean percentage of IL-6+ myeloid dendritic cells (mDCs) in total mDCs following CPE stimulation. Changes in frequency of IL-6 expressing mDCs following CPE stimulation between responders (diamond) and non-responders (square) of peanut oral immunotherapy during the first 24-weeks of therapy. Healthy controls (circle) were not treated and only assessed at baseline.
(PNG)

S25 Fig Scattered plot illustrating mean percentage of IL-10+ myeloid dendritic cells (mDCs) in total mDCs following LPS stimulation. Changes in frequency of IL-10 expressing mDCs following LPS stimulation between responders (diamond) and non-responders (square) of peanut oral immunotherapy during the first 24-weeks of therapy. Healthy controls (circle) were not treated and only assessed at baseline.
(PNG)

S26 Fig Scattered plot illustrating mean percentage of IL-10+ myeloid dendritic cells (mDCs) in total mDCs following CPE stimulation. Changes in frequency of IL-10 expressing mDCs following CPE stimulation between responders (diamond) and non-responders (square) of peanut oral immunotherapy during the first 24-weeks of therapy. Healthy controls (circle) were not treated and only assessed at baseline.
(PNG)

S27 Fig Scattered plot illustrating mean percentage of IL-4+ plasmacytoid dendritic cells (pDCs) in total pDCs following LPS stimulation. Changes in frequency of IL-4 expressing pDCs following LPS stimulation between responders (diamond) and non-responders (square) of peanut oral immunotherapy during the first 24-weeks of therapy. Healthy controls (circle) were not treated and only assessed at baseline.
(PNG)

S28 Fig Scattered plot illustrating mean percentage of IL-4+ plasmacytoid dendritic cells (pDCs) in total pDCs following CPE stimulation. Changes in frequency of IL-4 expressing pDCs following CPE stimulation between responders (diamond) and non-responders (square) of peanut oral immunotherapy during the first 24-weeks of therapy. Healthy controls (circle) were not treated and only assessed at baseline.
(PNG)

S29 Fig Scattered plot illustrating mean percentage of IL-6+ plasmacytoid dendritic cells (pDCs) in total pDCs following LPS stimulation. Changes in frequency of I6-4 expressing pDCs following LPS stimulation between responders (diamond) and non-responders (square) of peanut oral immunotherapy during the first 24-weeks of therapy. Healthy controls (circle) were not treated and only assessed at baseline.
(PNG)

S30 Fig Scattered plot illustrating mean percentage of IL-6+ plasmacytoid dendritic cells (pDCs) in total pDCs following CPE stimulation. Changes in frequency of IL-6 expressing pDCs following CPE stimulation between responders (diamond) and non-responders (square) of peanut oral immunotherapy during the first 24-weeks of therapy. Healthy controls (circle) were not treated and only assessed at baseline.
(PNG)

S31 Fig Scattered plot illustrating mean percentage of IL-10+ plasmacytoid dendritic cells (pDCs) in total pDCs following LPS stimulation. Changes in frequency of IL-10 expressing pDCs following LPS stimulation between responders (diamond) and non-responders (square) of peanut oral immunotherapy during the first 24-weeks of therapy. Healthy controls (circle) were not treated and only assessed at baseline.
(PNG)

S32 Fig Scattered plot illustrating mean percentage of IL-10+ plasmacytoid dendritic cells (pDCs) in total pDCs following CPE stimulation. Changes in frequency of IL-10 expressing pDCs following CPE stimulation between responders (diamond) and non-responders (square) of peanut oral immunotherapy during the first 24-weeks of therapy. Healthy controls (circle) were not treated and only assessed at baseline.
(PNG)

S33 Fig Scattered plot illustrating mean percentage of TNFa+ plasmacytoid dendritic cells (pDCs) in total pDCs following LPS stimulation. Changes in frequency of TNFa expressing pDCs following LPS stimulation between responders (diamond) and non-responders (square) of peanut oral immunotherapy during the first 24-weeks of therapy. Healthy controls (circle) were not treated and only assessed at baseline.
(PNG)

S34 Fig Scattered plot illustrating mean percentage of TNFa+ plasmacytoid dendritic cells (pDCs) in total pDCs following CPE stimulation. Changes in frequency of TNFa expressing pDCs following CPE stimulation between responders (diamond) and non-responders (square) of peanut oral immunotherapy during the first 24-weeks of therapy. Healthy controls (circle) were not treated and only assessed at baseline.
(PNG)

S35 Fig Scattered plot illustrating mean percentage of total myeloid dendritic cells (mDCs) in total PBMCs following LPS stimulation. Changes in frequency of total mDCs following LPS stimulation between responders (diamond) and non-responders (square) of peanut oral immunotherapy during the first 24-weeks of therapy. Healthy controls (circle) were not treated and only assessed at baseline.
(PNG)

S36 Fig Scattered plot illustrating mean percentage of total myeloid dendritic cells (mDCs) in total PBMCs following CPE stimulation. Changes in frequency of total mDCs following CPE stimulation between responders (diamond) and non-responders (square) of peanut oral immunotherapy during the first 24-weeks of therapy. Healthy controls (circle) were not treated and only assessed at baseline.
(PNG)

S37 Fig Scattered plot illustrating mean percentage of total plasmacytoid dendritic cells (pDCs) in total PBMCs following LPS stimulation. Changes in frequency of total pDCs following LPS stimulation between responders (diamond) and non-responders (square) of peanut oral immunotherapy during the first 24-weeks of therapy. Healthy controls (circle) were not treated and only assessed at baseline.
(PNG)

S38 Fig Scattered plot illustrating mean percentage of total plasmacytoid dendritic cells (pDCs) in total PBMCs following CPE stimulation. Changes in frequency of total pDCs following CPE stimulation between responders (diamond) and non-responders (square) of peanut oral immunotherapy during the first 24-weeks of therapy. Healthy controls (circle) were not treated and only assessed at baseline.
(PNG)

S1 File CSV file for mean population frequencies. (CSV)

S2 File DC summary statistics. (RTF)
