# Supplementary figures and images for: Reduced pro-inflammatory dendritic cell phenotypes are a potential indicator of successful peanut oral immunotherapy
Source: PLoS One. 2022 May 26;17(5):e0264674. doi: 10.1371/journal.pone.0264674 (PMC9135258; doi:10.1371/journal.pone.0264674)

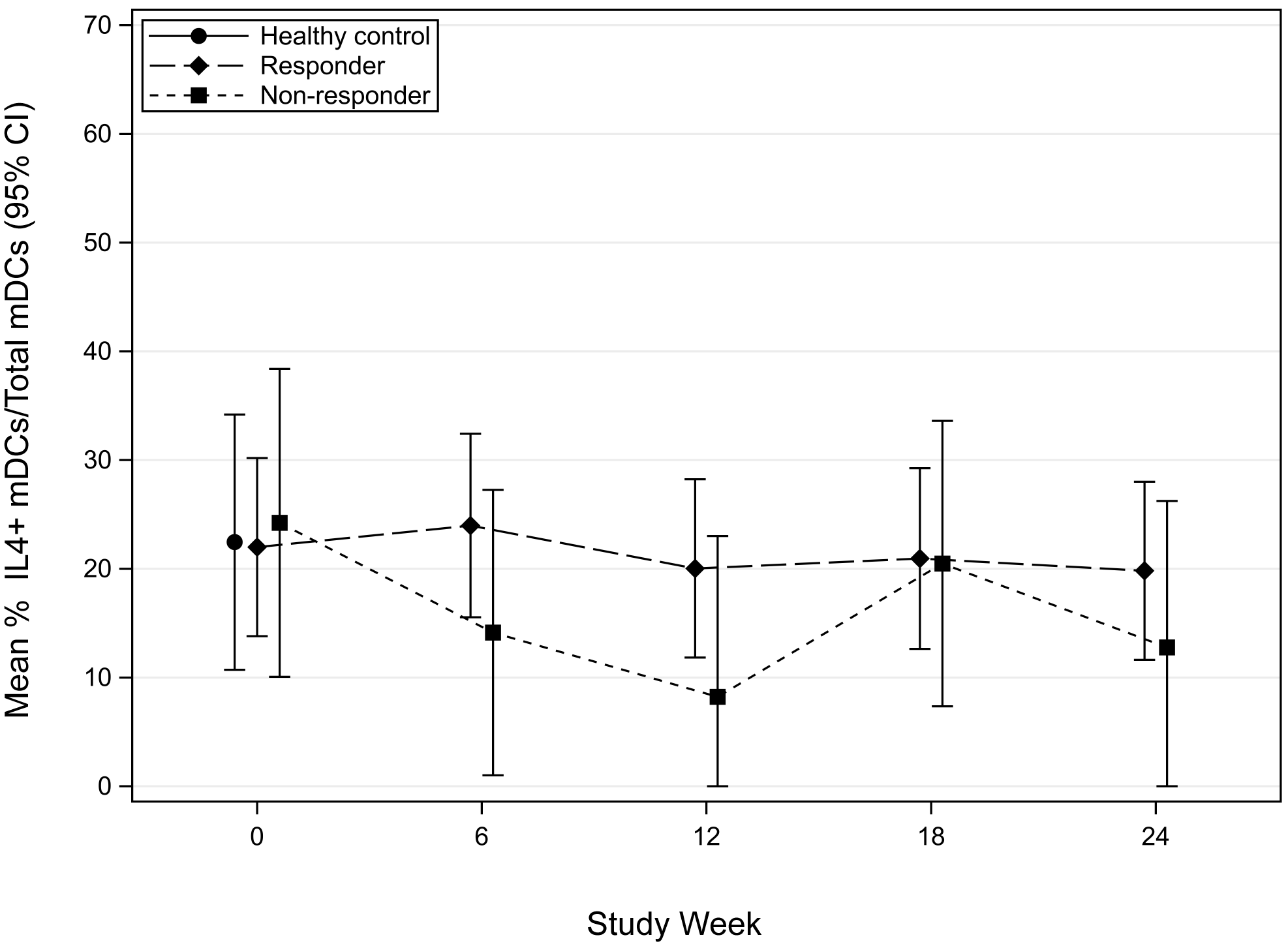

Supplement: S1 Fig — Changes in frequency of IL-4 expressing mDCs following LPS stimulation between responders (diamond) and non-responders (square) of peanut oral immunotherapy during the first 24-weeks of therapy. Healthy controls (circle) were not treated and only assessed at baseline. (TIF) [file pone.0264674.s001.tif]

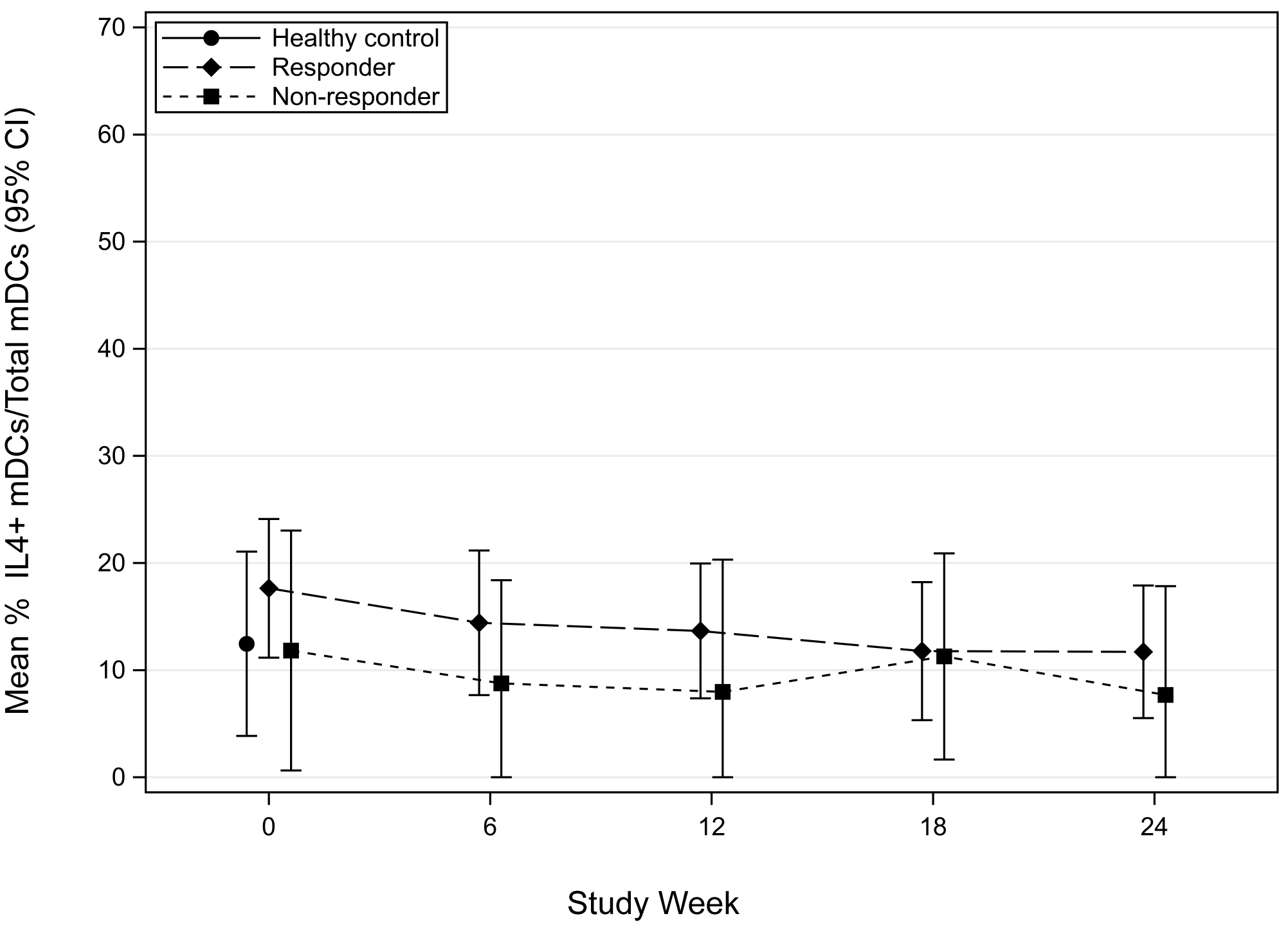

Supplement: S2 Fig — Changes in frequency of IL-4 expressing mDCs following CPE stimulation between responders (diamond) and non-responders (square) of peanut oral immunotherapy during the first 24-weeks of therapy. Healthy controls (circle) were not treated and only assessed at baseline. (TIF) [file pone.0264674.s002.tif]

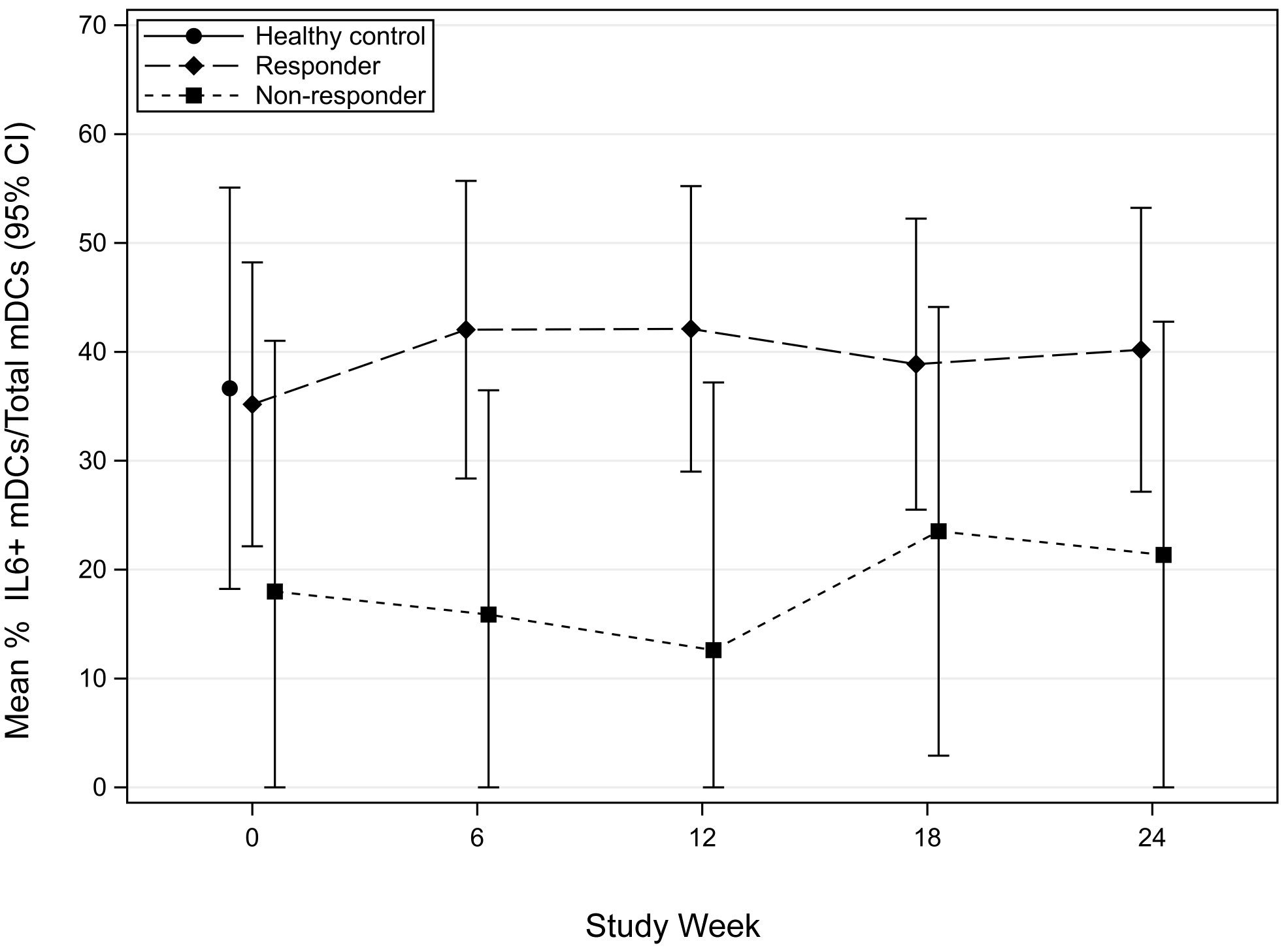

Supplement: S3 Fig — Changes in frequency of IL-6 expressing mDCs following LPS stimulation between responders (diamond) and non-responders (square) of peanut oral immunotherapy during the first 24-weeks of therapy. Healthy controls (circle) were not treated and only assessed at baseline. (TIF) [file pone.0264674.s003.tif]

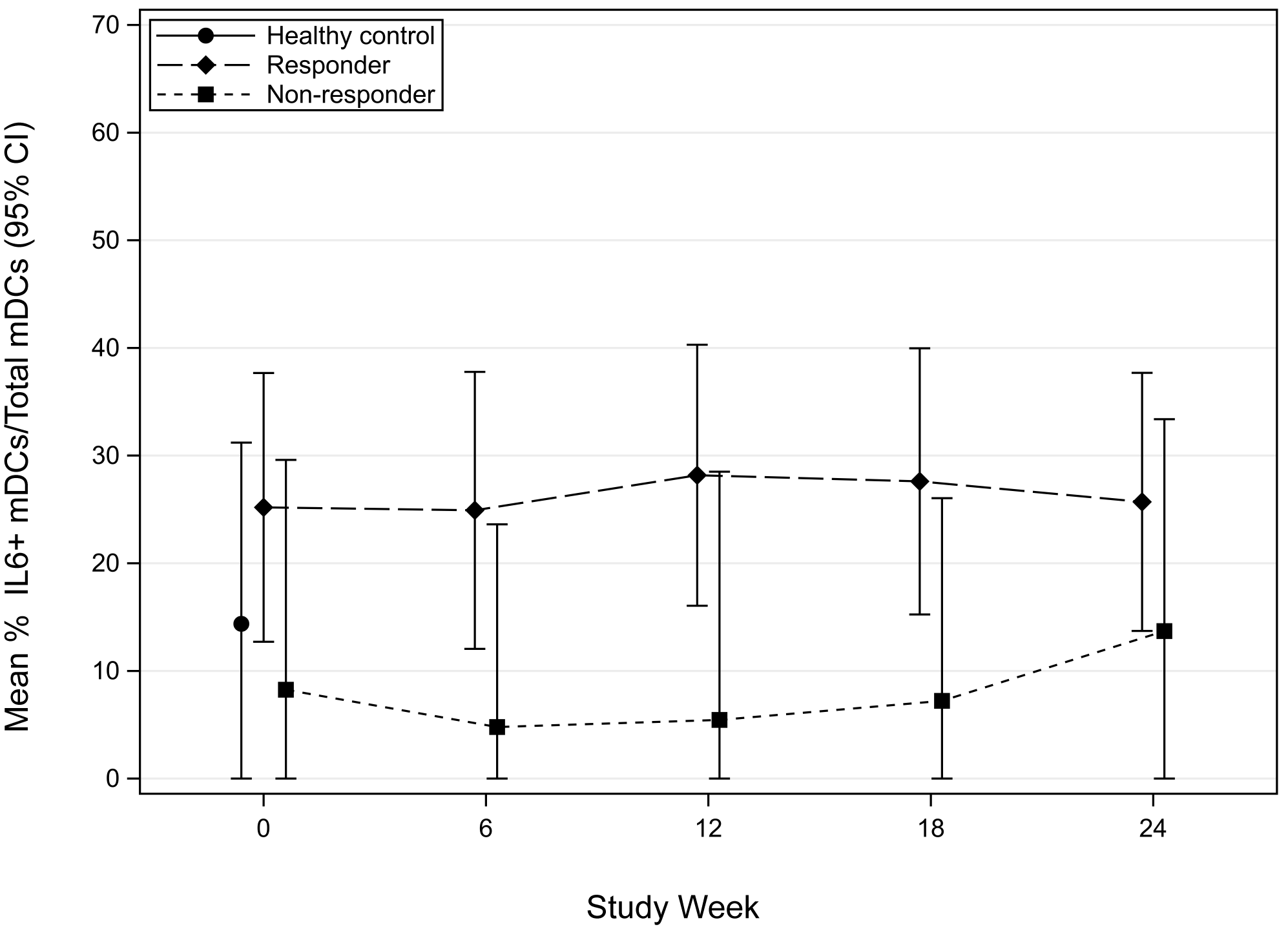

Supplement: S4 Fig — Changes in frequency of IL-6 expressing mDCs following CPE stimulation between responders (diamond) and non-responders (square) of peanut oral immunotherapy during the first 24-weeks of therapy. Healthy controls (circle) were not treated and only assessed at baseline. (TIF) [file pone.0264674.s004.tif]

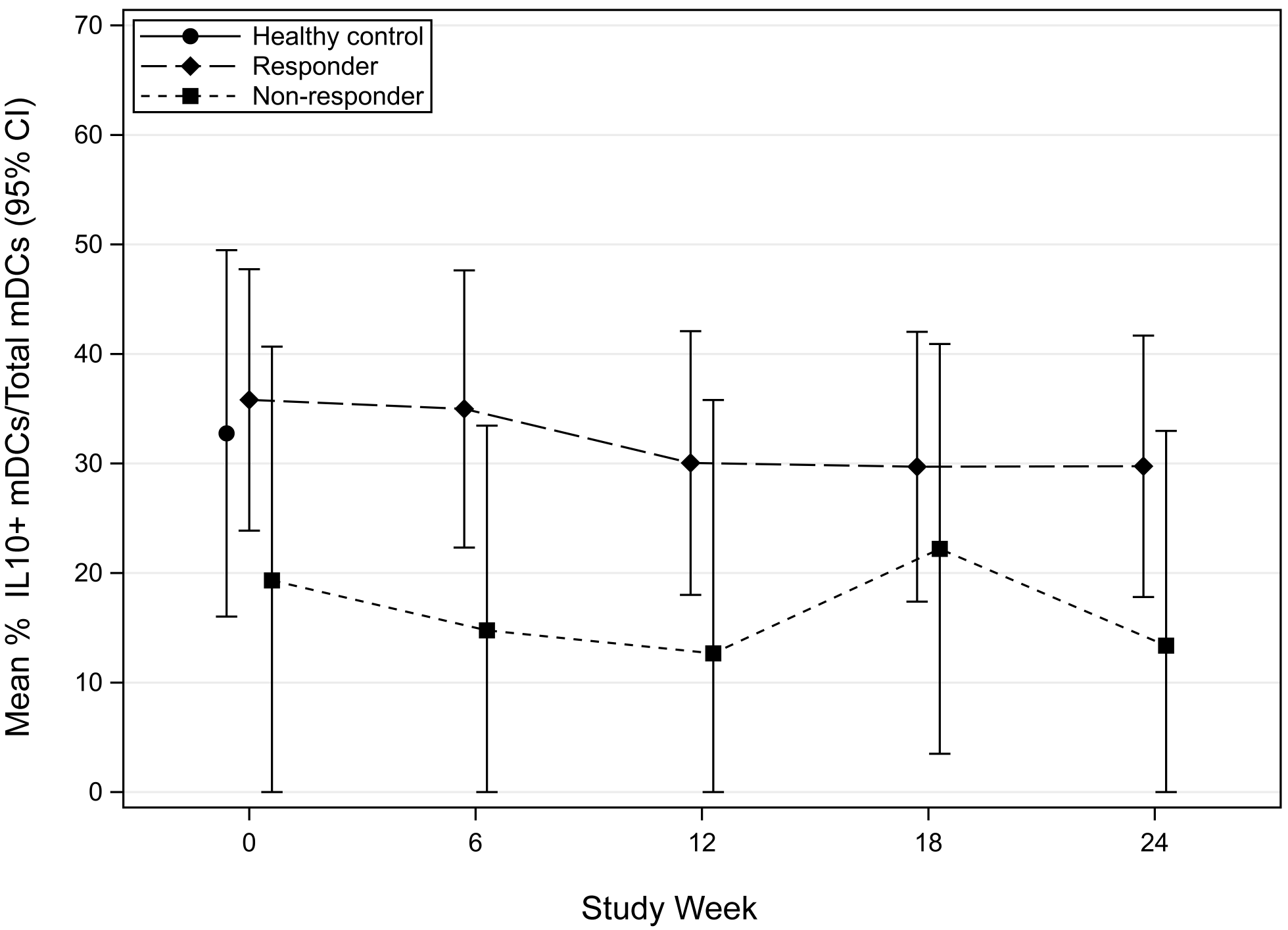

Supplement: S5 Fig — Changes in frequency of IL-10 expressing mDCs following LPS stimulation between responders (diamond) and non-responders (square) of peanut oral immunotherapy during the first 24-weeks of therapy. Healthy controls (circle) were not treated and only assessed at baseline. (TIF) [file pone.0264674.s005.tif]

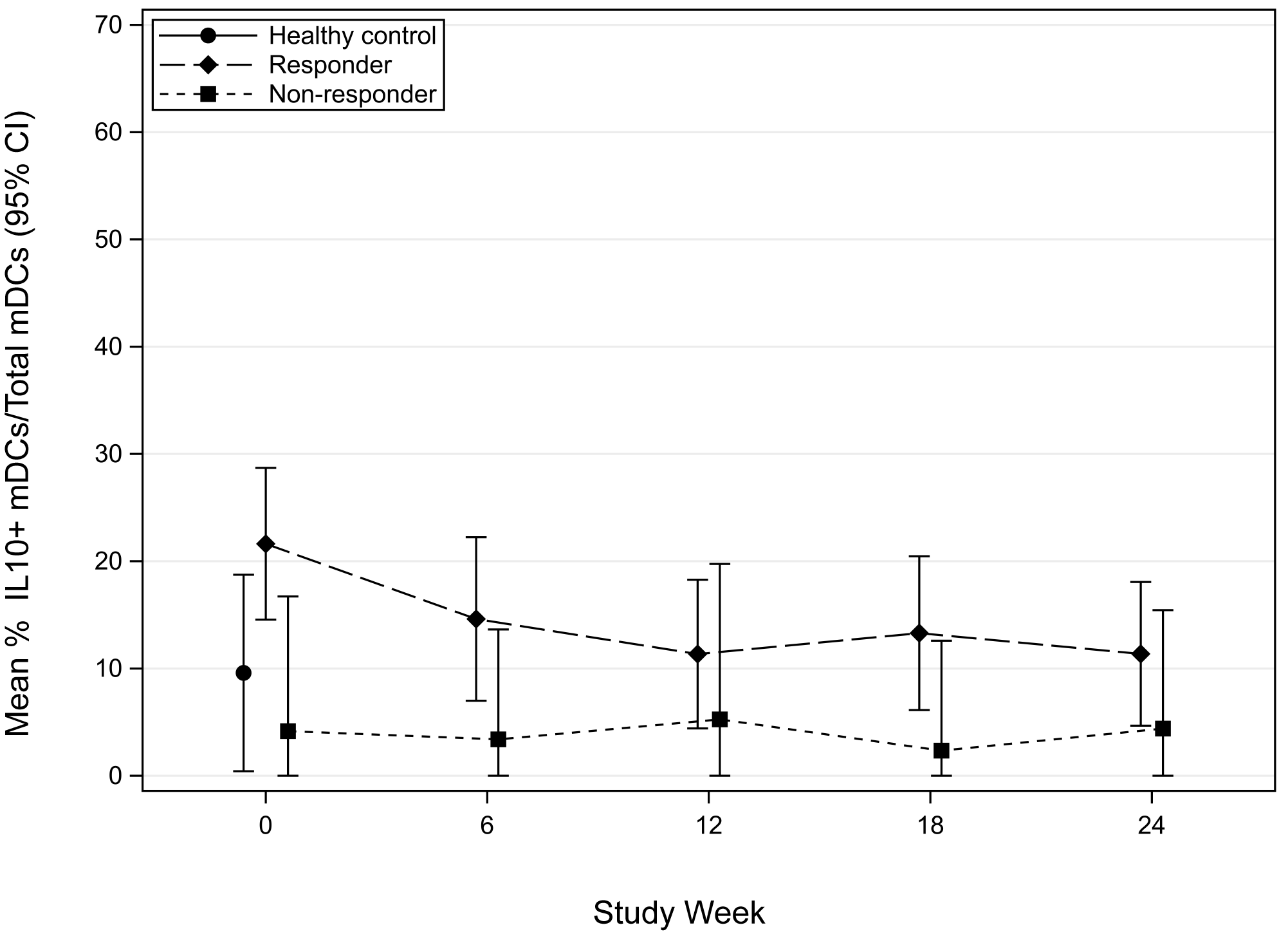

Supplement: S6 Fig — Changes in frequency of IL-10 expressing mDCs following CPE stimulation between responders (diamond) and non-responders (square) of peanut oral immunotherapy during the first 24-weeks of therapy. Healthy controls (circle) were not treated and only assessed at baseline. (TIF) [file pone.0264674.s006.tif]

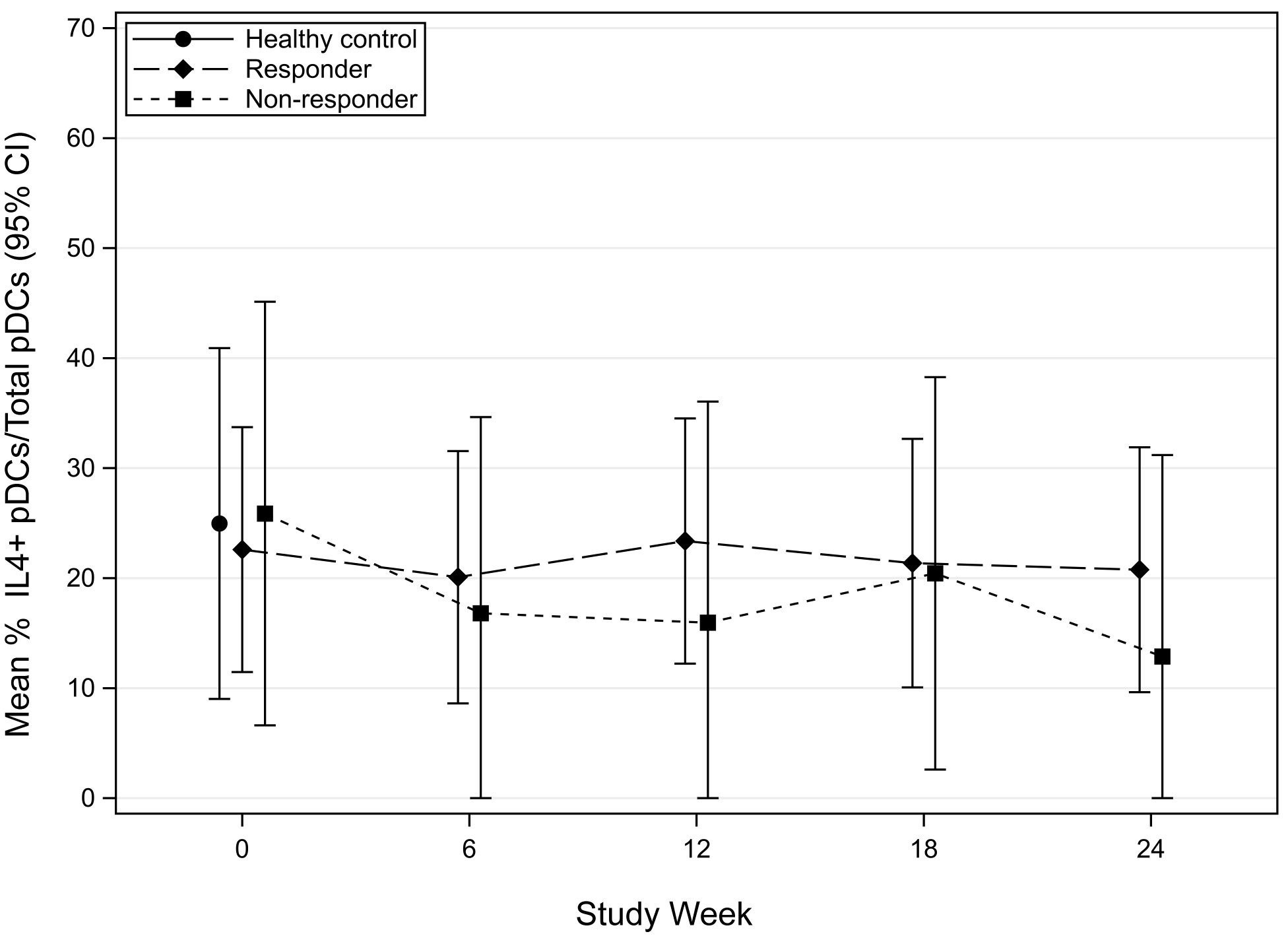

Supplement: S7 Fig — Changes in frequency of IL-4 expressing pDCs following LPS stimulation between responders (diamond) and non-responders (square) of peanut oral immunotherapy during the first 24-weeks of therapy. Healthy controls (circle) were not treated and only assessed at baseline. (TIF) [file pone.0264674.s007.tif]

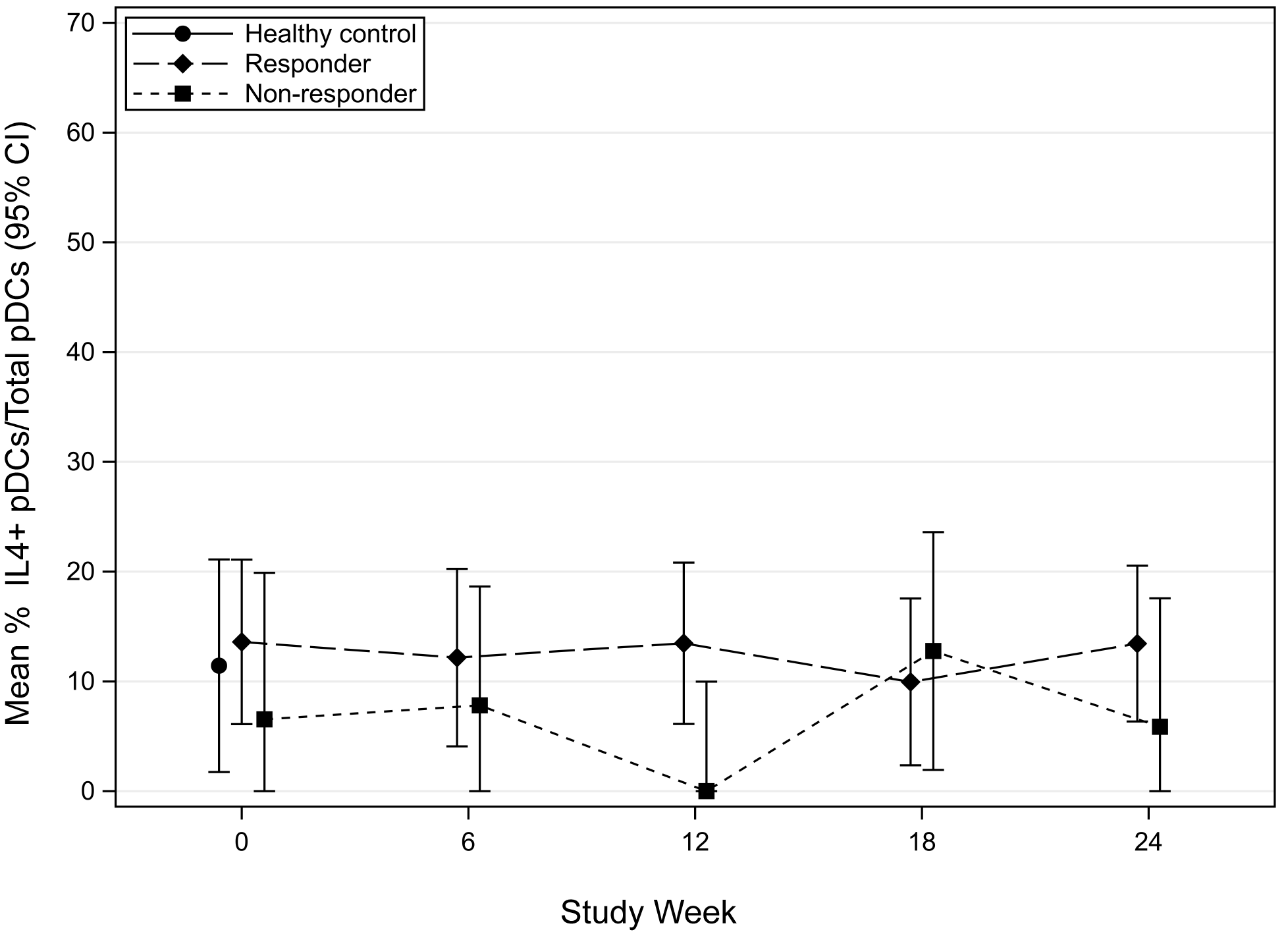

Supplement: S8 Fig — Changes in frequency of IL-4 expressing pDCs following CPE stimulation between responders (diamond) and non-responders (square) of peanut oral immunotherapy during the first 24-weeks of therapy. Healthy controls (circle) were not treated and only assessed at baseline. (TIF) [file pone.0264674.s008.tif]

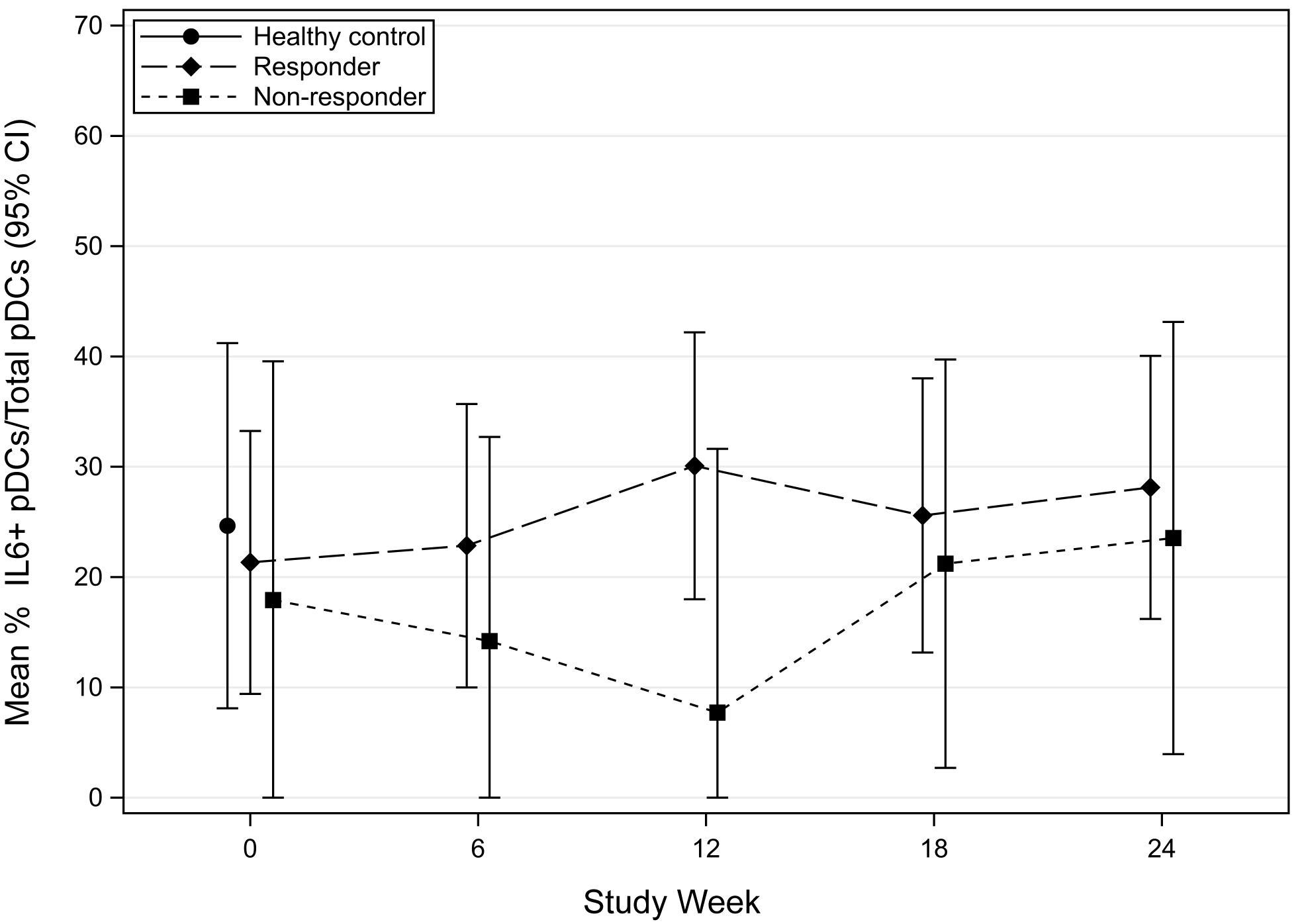

Supplement: S9 Fig — Changes in frequency of I6-4 expressing pDCs following LPS stimulation between responders (diamond) and non-responders (square) of peanut oral immunotherapy during the first 24-weeks of therapy. Healthy controls (circle) were not treated and only assessed at baseline. (TIF) [file pone.0264674.s009.tif]

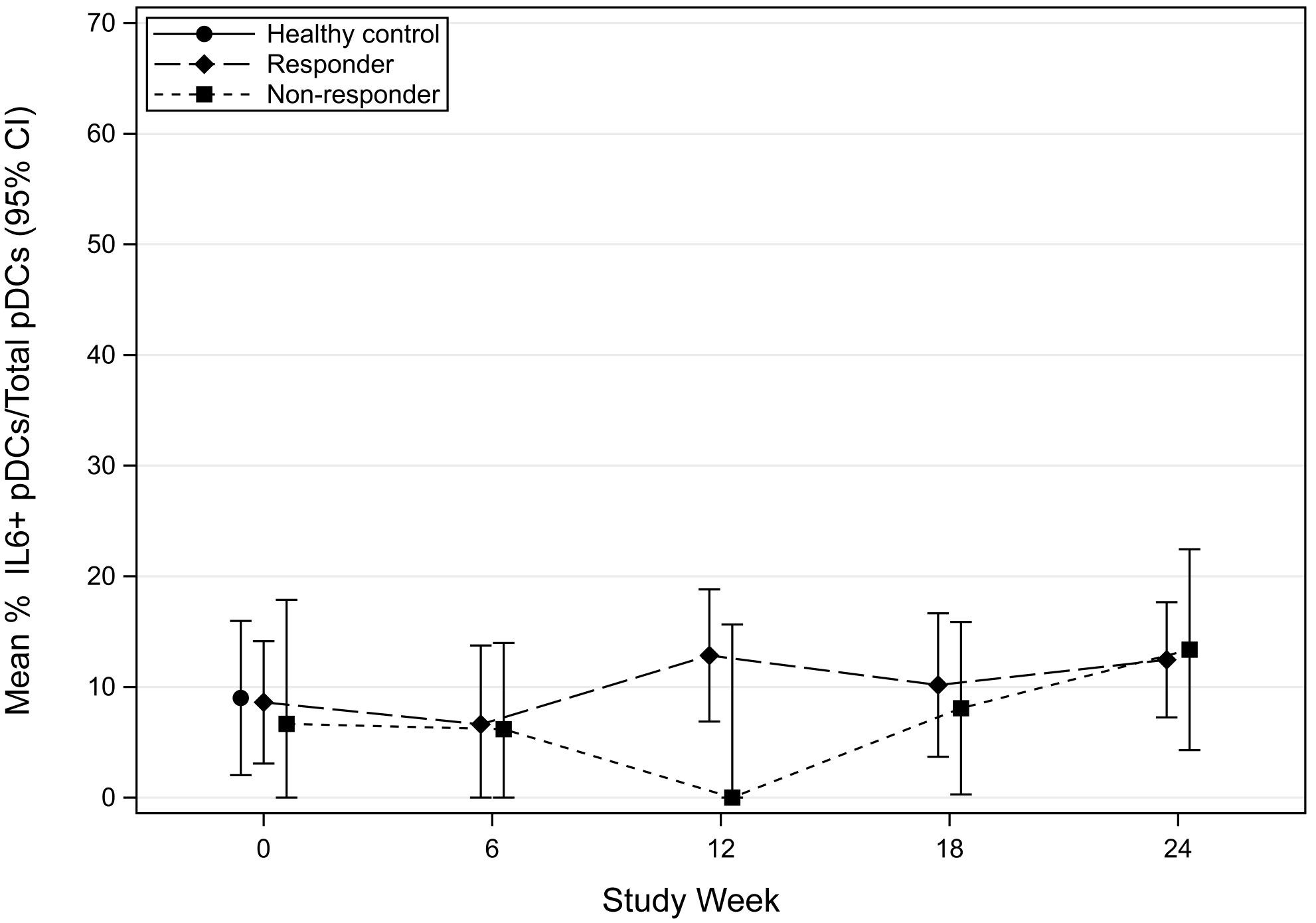

Supplement: S10 Fig — Changes in frequency of IL-6 expressing pDCs following CPE stimulation between responders (diamond) and non-responders (square) of peanut oral immunotherapy during the first 24-weeks of therapy. Healthy controls (circle) were not treated and only assessed at baseline. (TIF) [file pone.0264674.s010.tif]

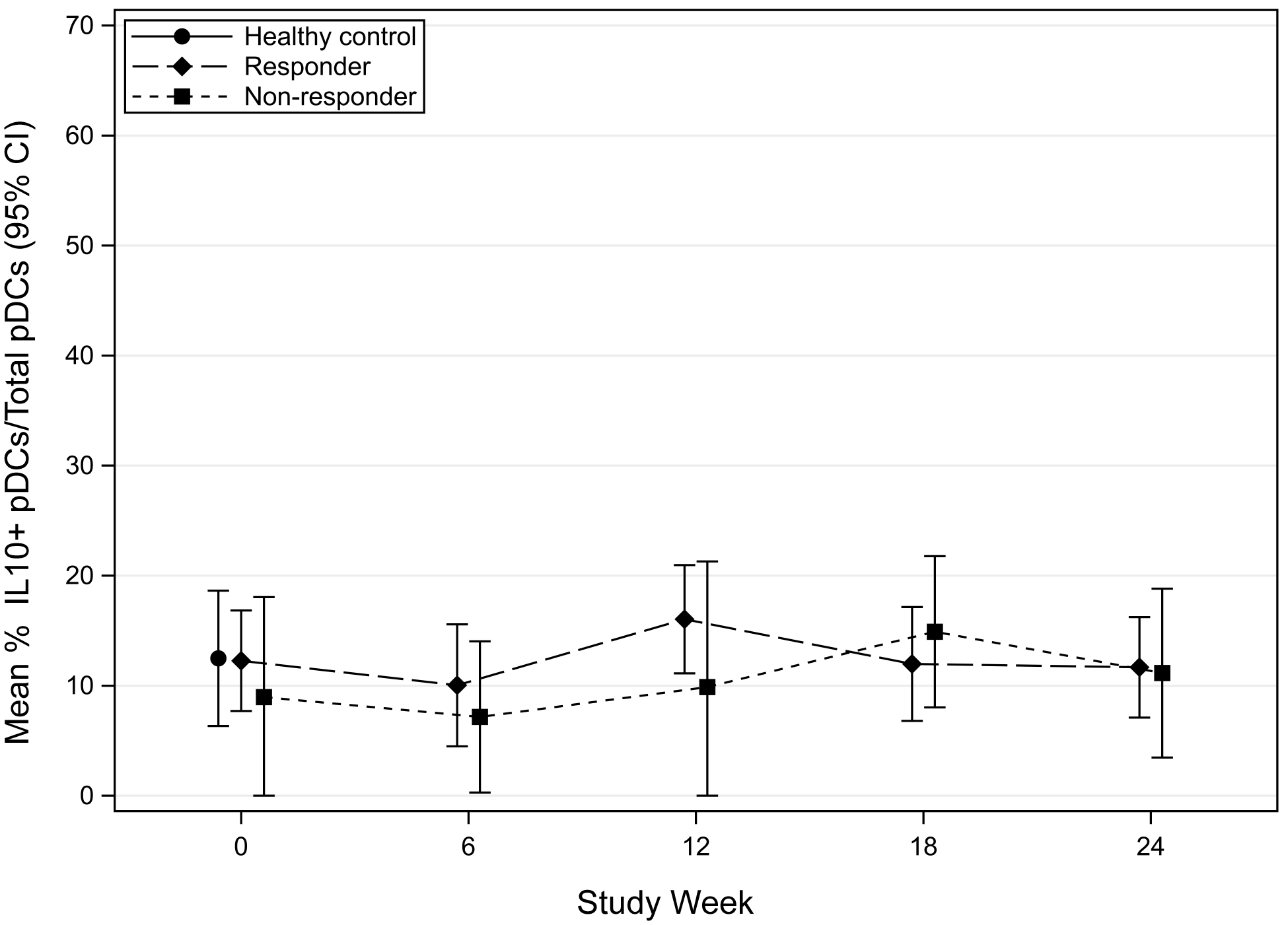

Supplement: S11 Fig — Changes in frequency of IL-10 expressing pDCs following LPS stimulation between responders (diamond) and non-responders (square) of peanut oral immunotherapy during the first 24-weeks of therapy. Healthy controls (circle) were not treated and only assessed at baseline. (TIF) [file pone.0264674.s011.tif]

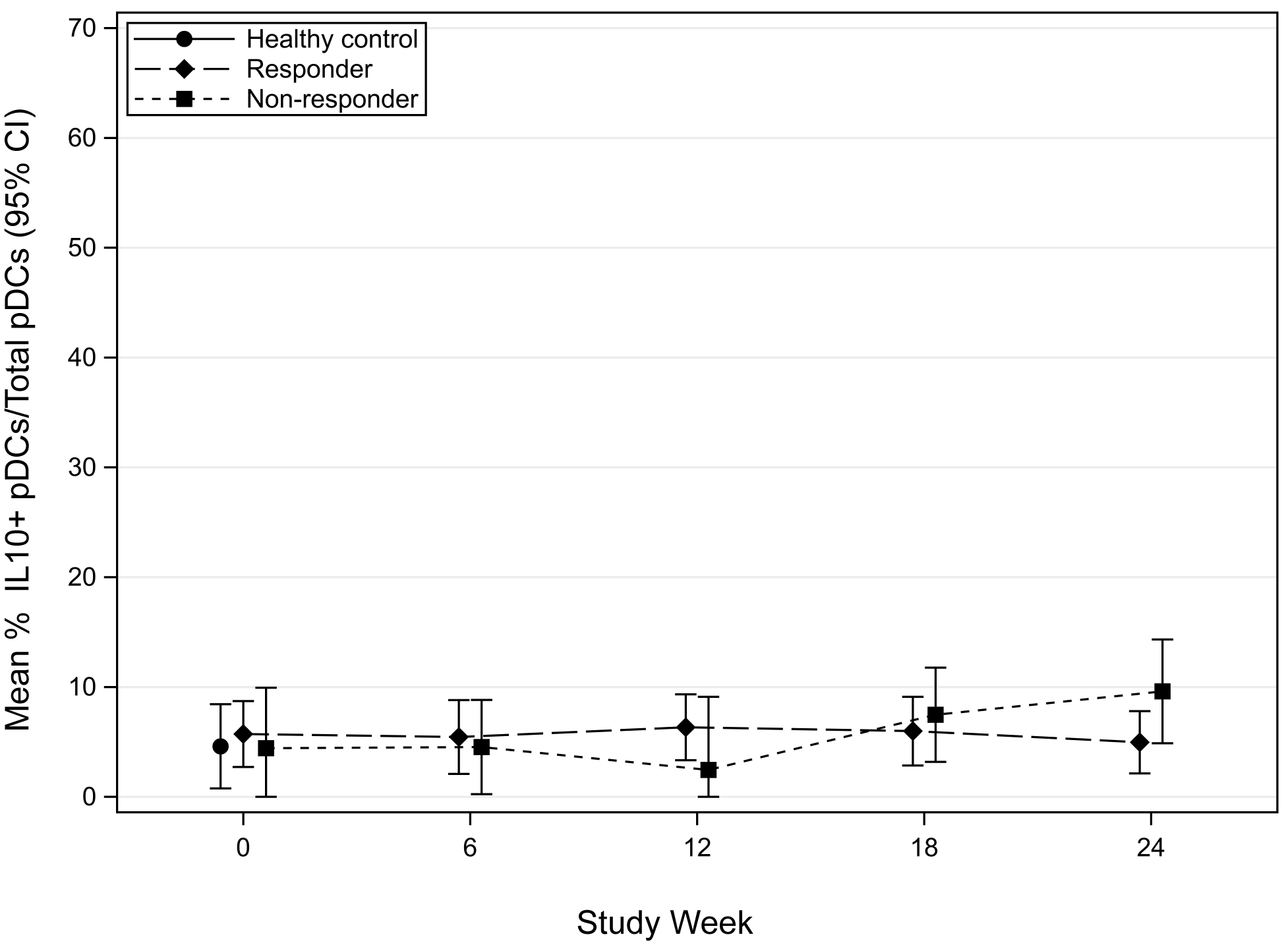

Supplement: S12 Fig — Changes in frequency of IL-10 expressing pDCs following CPE stimulation between responders (diamond) and non-responders (square) of peanut oral immunotherapy during the first 24-weeks of therapy. Healthy controls (circle) were not treated and only assessed at baseline. (TIF) [file pone.0264674.s012.tif]

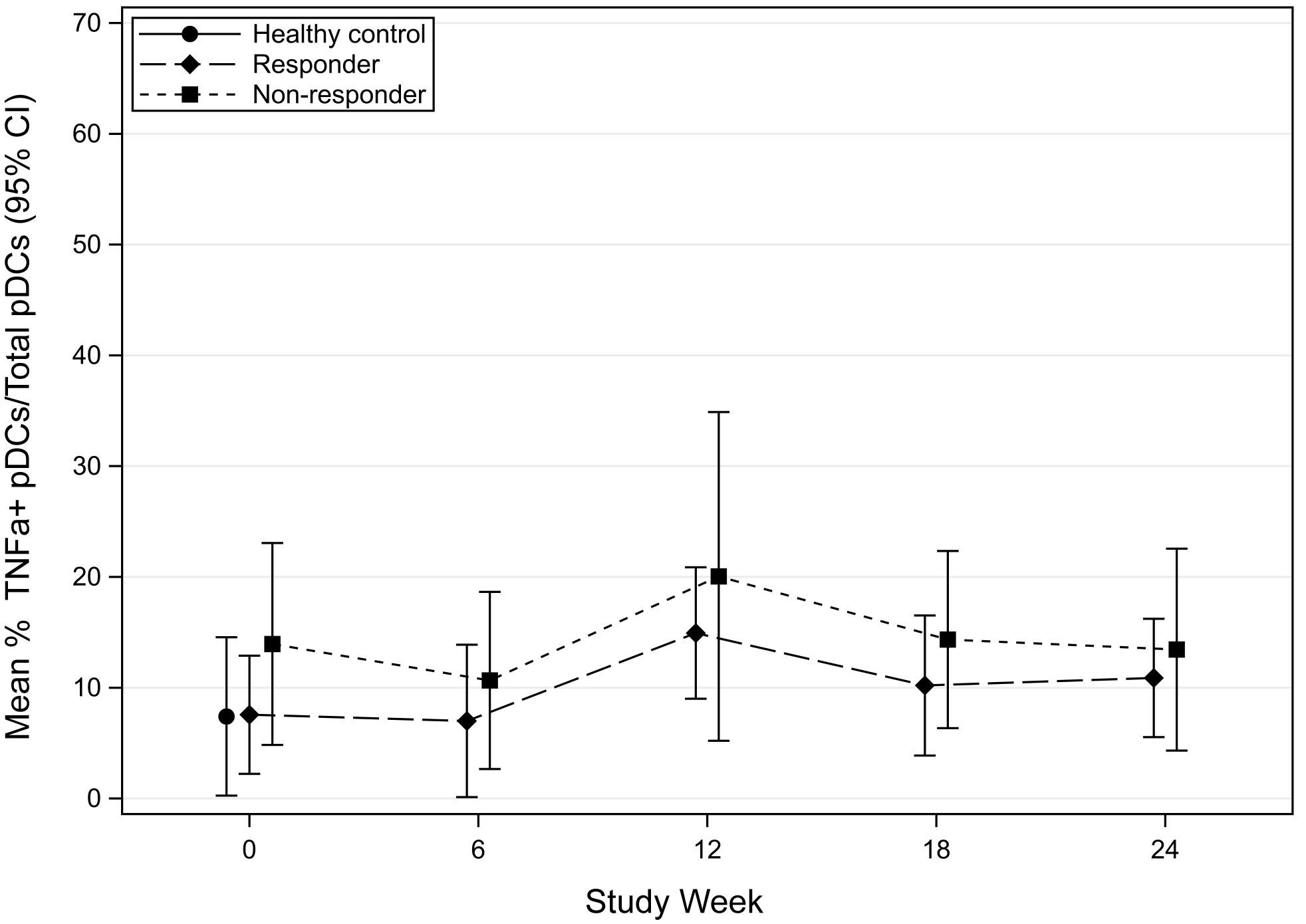

Supplement: S13 Fig — Changes in frequency of TNFa expressing pDCs following LPS stimulation between responders (diamond) and non-responders (square) of peanut oral immunotherapy during the first 24-weeks of therapy. Healthy controls (circle) were not treated and only assessed at baseline. (TIF) [file pone.0264674.s013.tif]

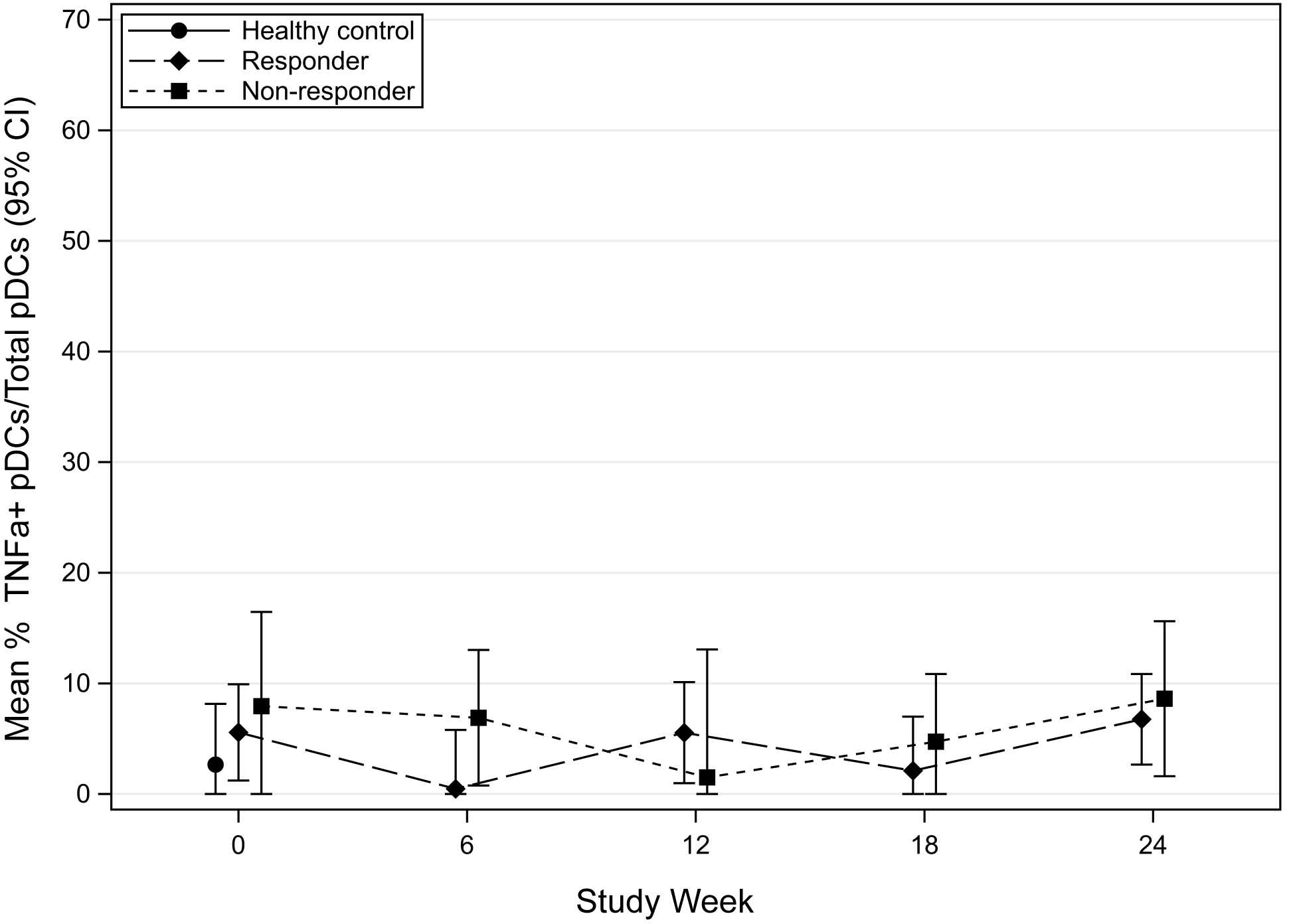

Supplement: S14 Fig — Changes in frequency of TNFa expressing pDCs following CPE stimulation between responders (diamond) and non-responders (square) of peanut oral immunotherapy during the first 24-weeks of therapy. Healthy controls (circle) were not treated and only assessed at baseline. (TIF) [file pone.0264674.s014.tif]

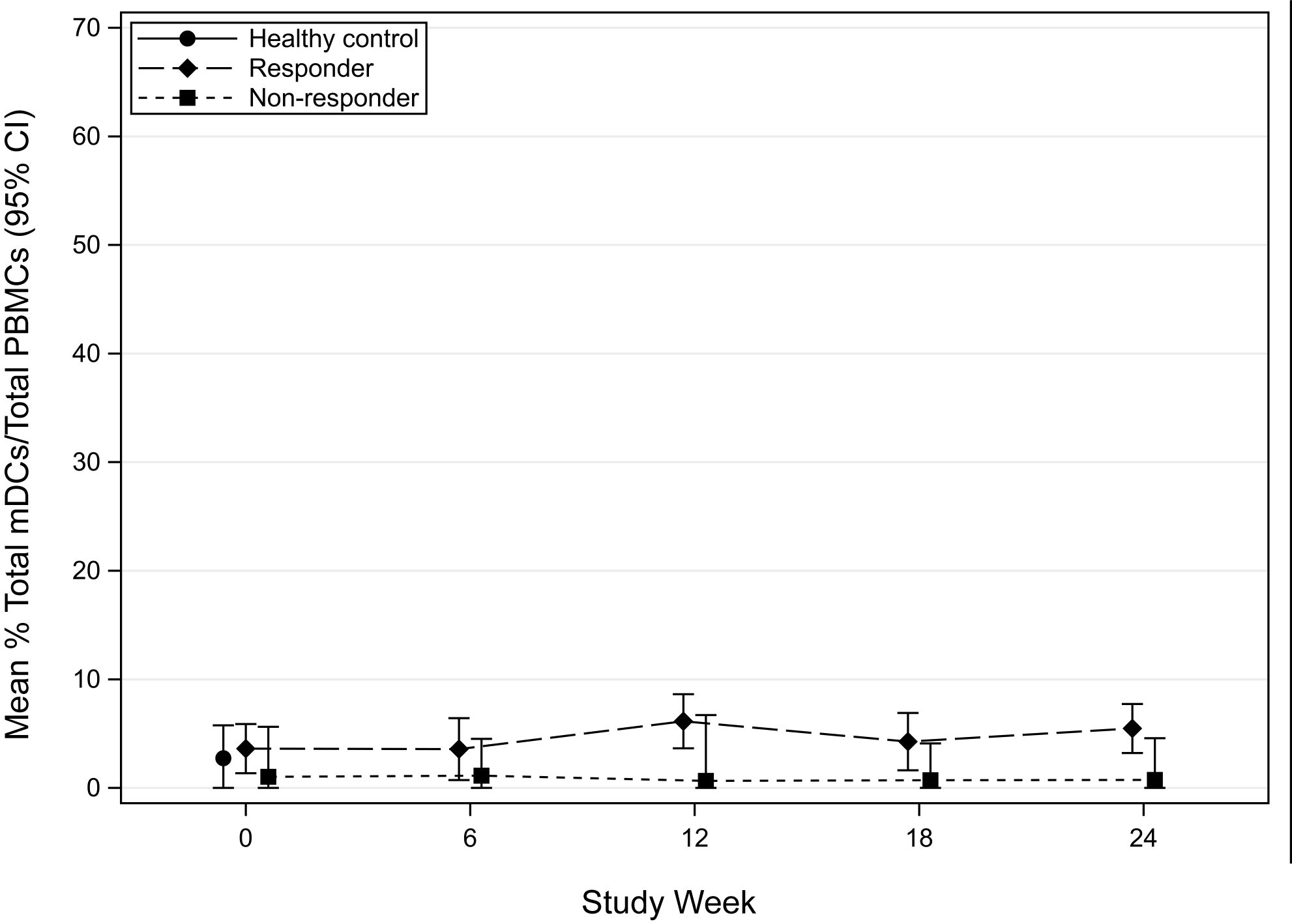

Supplement: S15 Fig — Changes in frequency of total mDCs following LPS stimulation between responders (diamond) and non-responders (square) of peanut oral immunotherapy during the first 24-weeks of therapy. Healthy controls (circle) were not treated and only assessed at baseline. (TIF) [file pone.0264674.s015.tif]

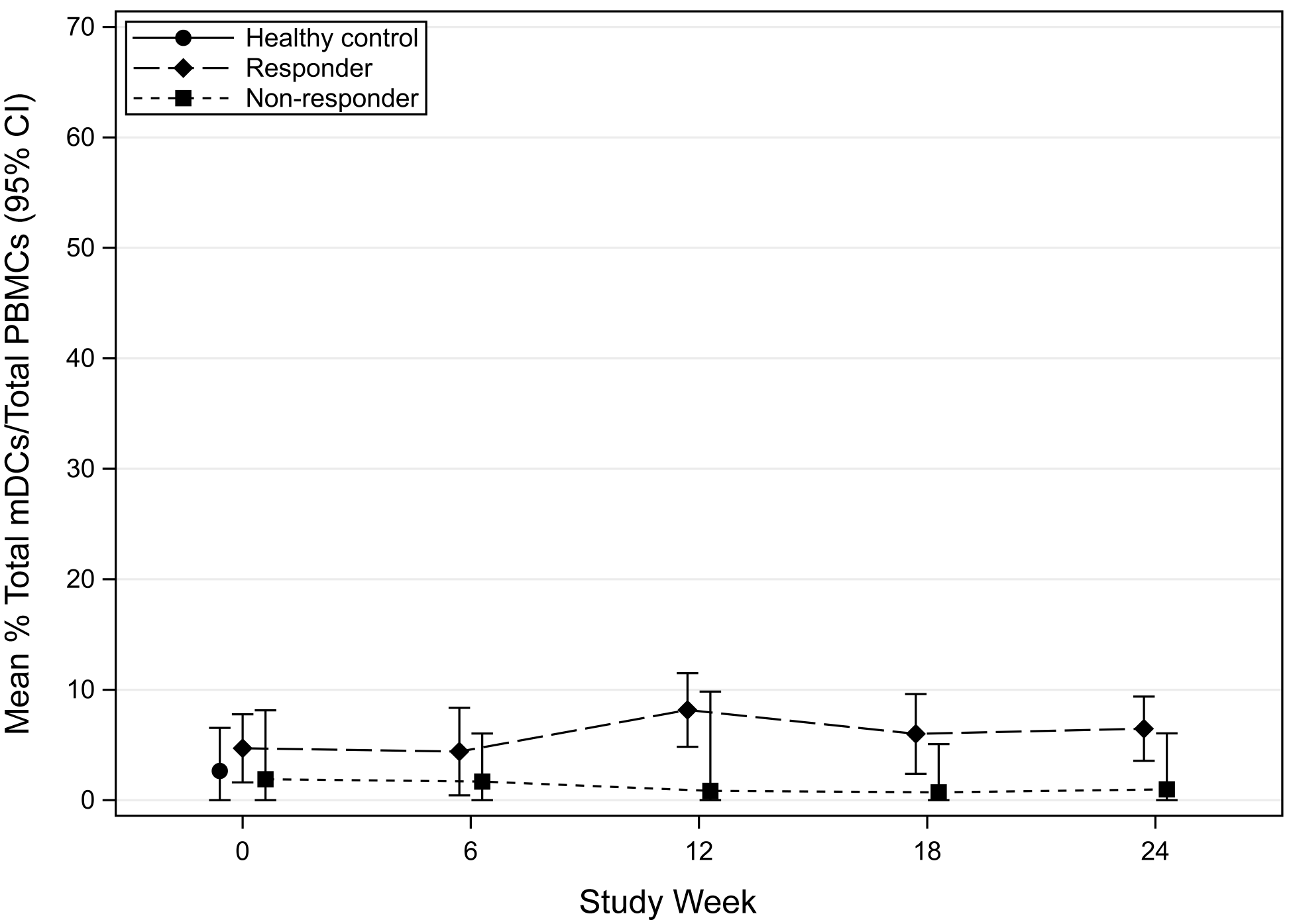

Supplement: S16 Fig — Changes in frequency of total mDCs following CPE stimulation between responders (diamond) and non-responders (square) of peanut oral immunotherapy during the first 24-weeks of therapy. Healthy controls (circle) were not treated and only assessed at baseline. (TIF) [file pone.0264674.s016.tif]

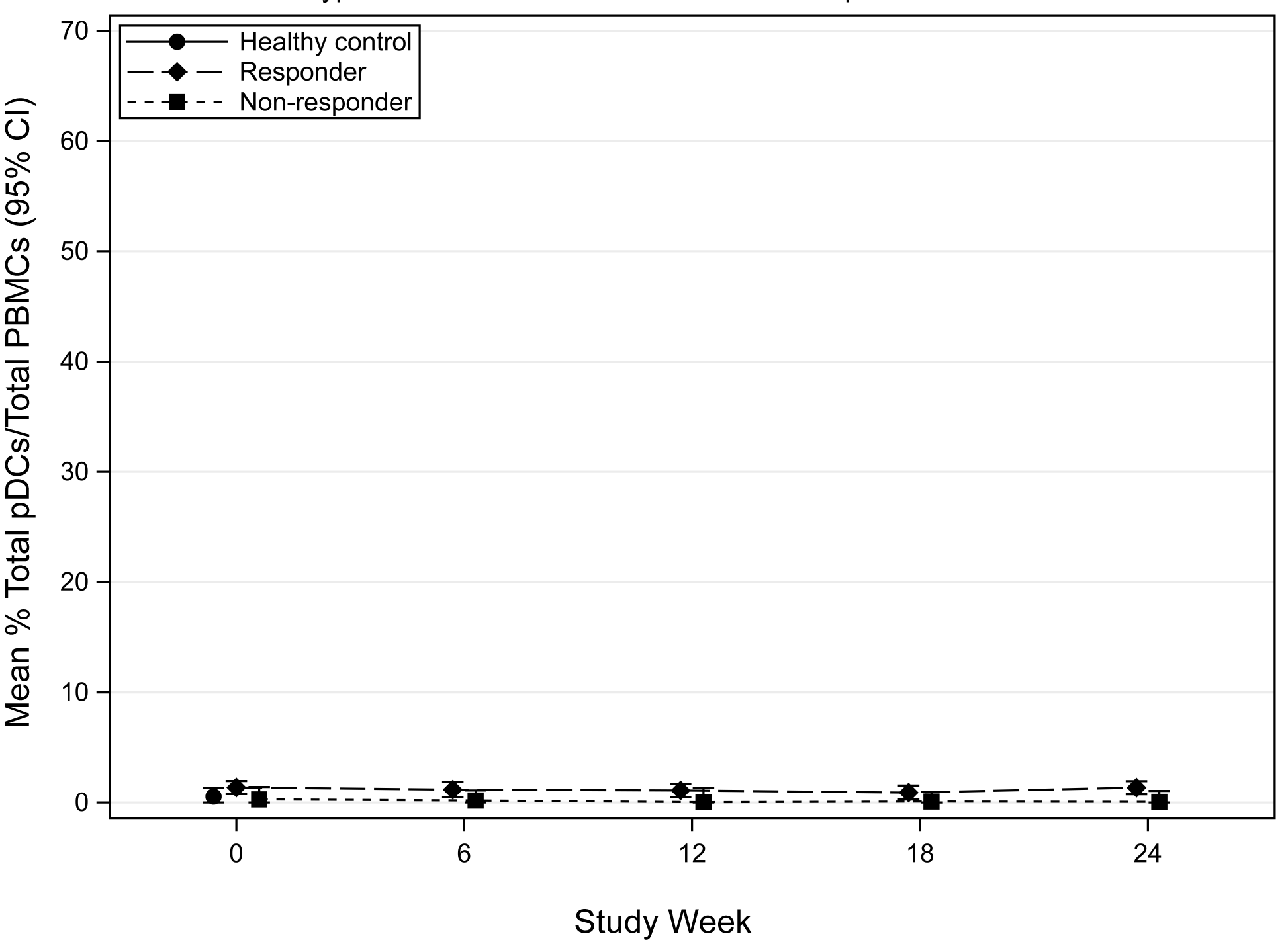

Supplement: S17 Fig — Changes in frequency of total pDCs following LPS stimulation between responders (diamond) and non-responders (square) of peanut oral immunotherapy during the first 24-weeks of therapy. Healthy controls (circle) were not treated and only assessed at baseline. (TIF) [file pone.0264674.s017.tif]

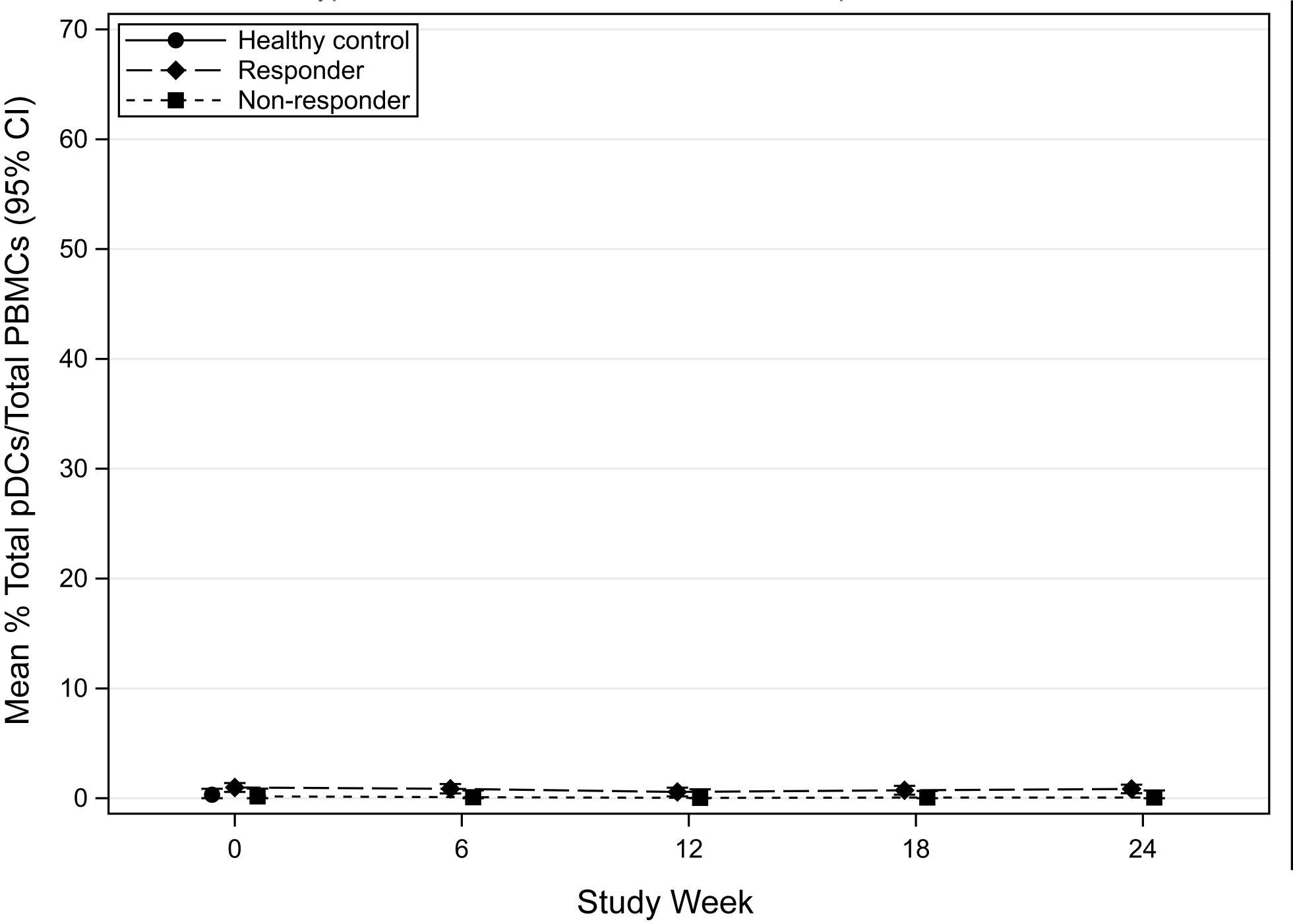

Supplement: S18 Fig — Changes in frequency of total pDCs following CPE stimulation between responders (diamond) and non-responders (square) of peanut oral immunotherapy during the first 24-weeks of therapy. Healthy controls (circle) were not treated and only assessed at baseline. (TIF) [file pone.0264674.s018.tif]

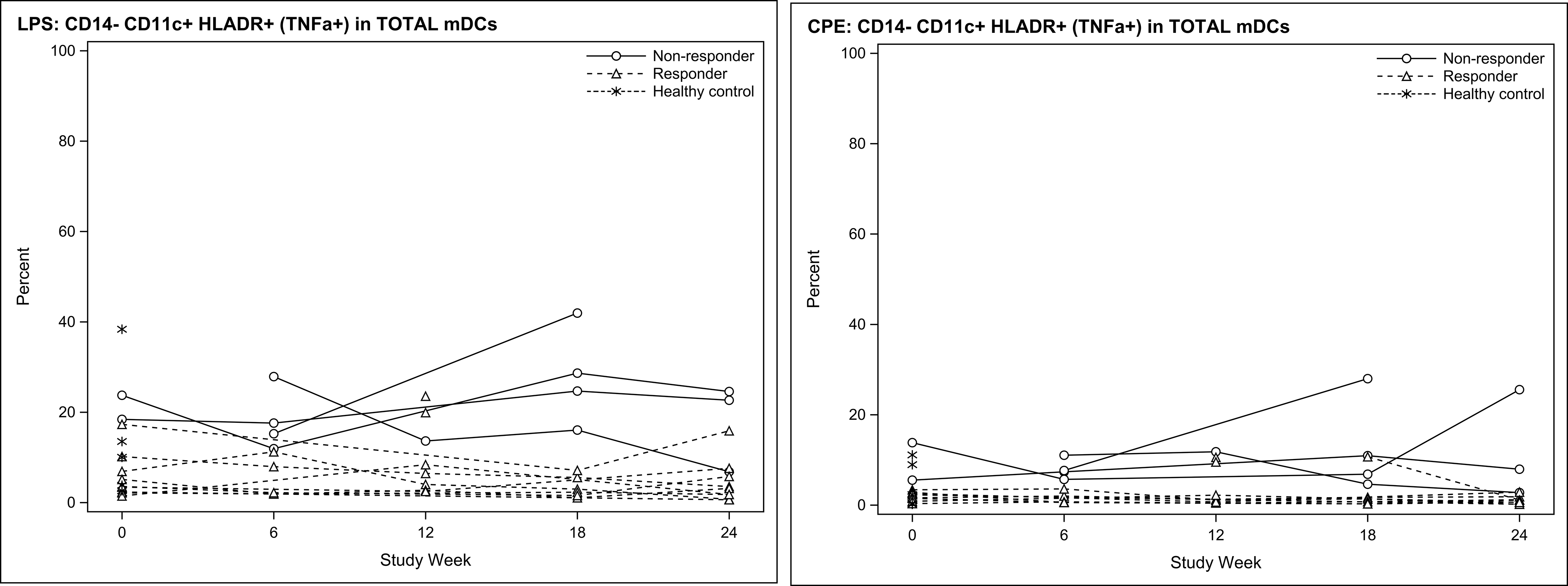

Supplement: S19 Fig — (A) Mean percent frequency of TNFa-production mDCs following LPS stimulation; (B) Mean percent frequency of TNFa-production mDCs following CPE stimulation. (PNG) [file pone.0264674.s019.png]

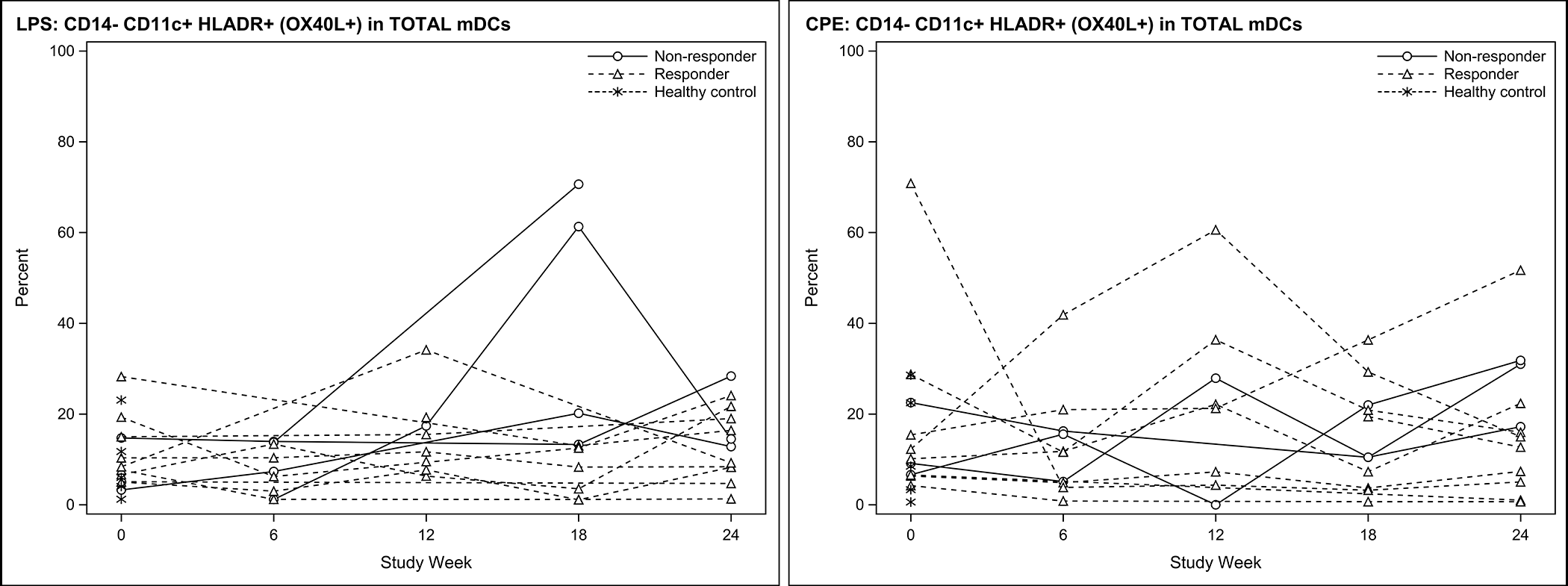

Supplement: S20 Fig — (A) Mean percent frequency (95% CI) of OX40L mDCs following LPS stimulation, (B) Mean percent frequency (95% CI) of OX40L mDCs following CPE stimulation. (PNG) [file pone.0264674.s020.png]

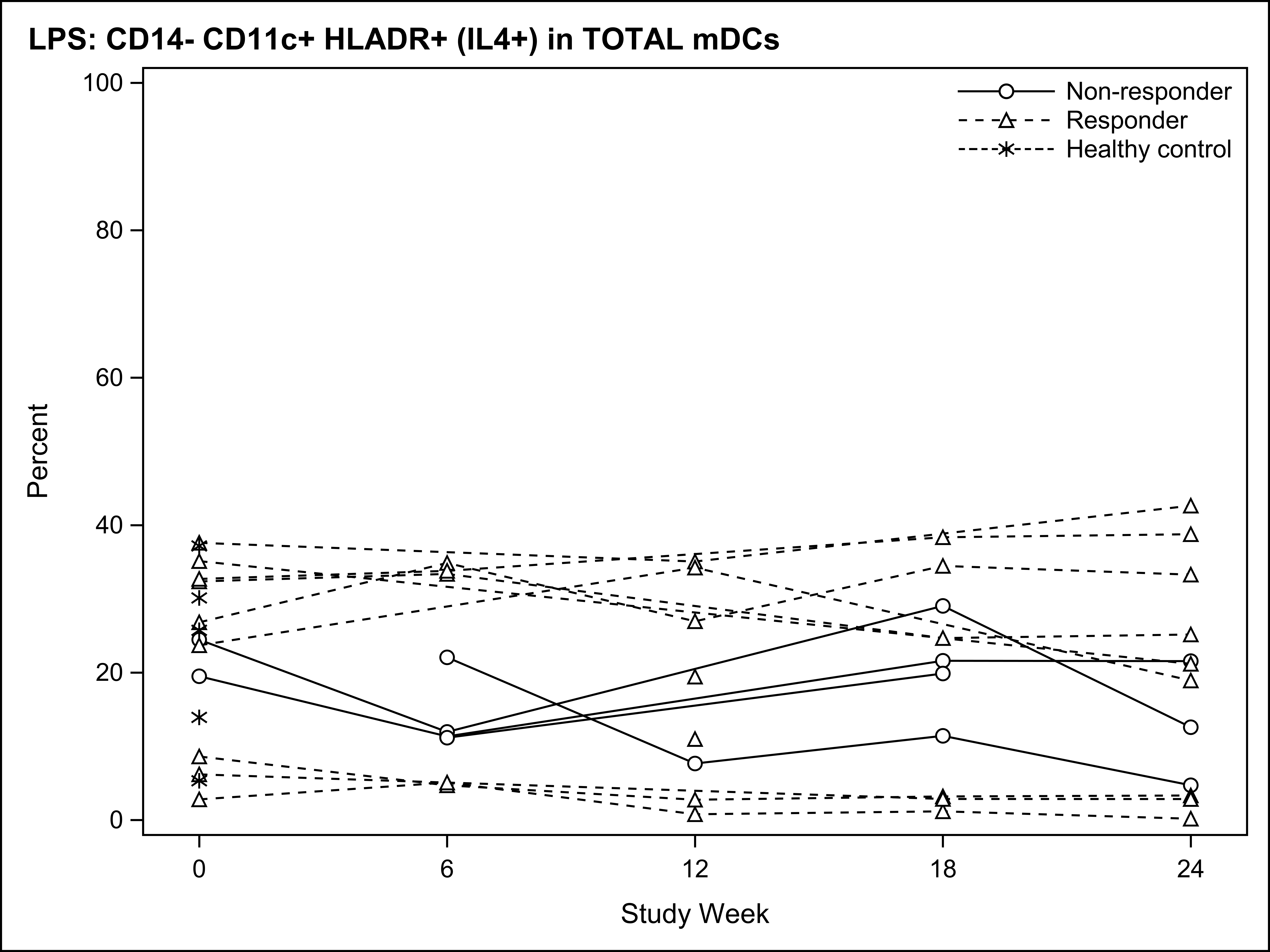

Supplement: S21 Fig — Changes in frequency of IL-4 expressing mDCs following LPS stimulation between responders (diamond) and non-responders (square) of peanut oral immunotherapy during the first 24-weeks of therapy. Healthy controls (circle) were not treated and only assessed at baseline. (PNG) [file pone.0264674.s021.png]

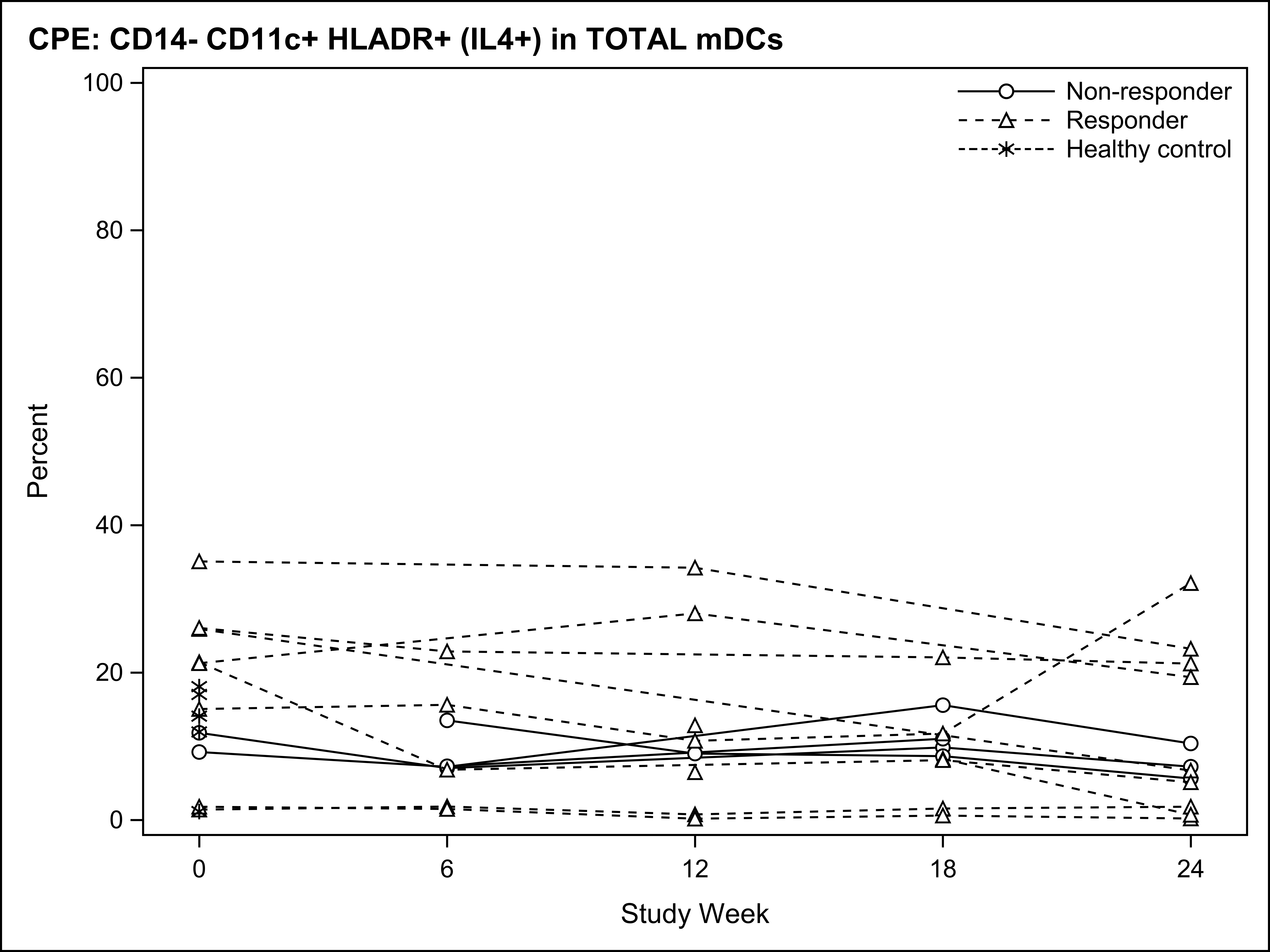

Supplement: S22 Fig — Changes in frequency of IL-4 expressing mDCs following CPE stimulation between responders (diamond) and non-responders (square) of peanut oral immunotherapy during the first 24-weeks of therapy. Healthy controls (circle) were not treated and only assessed at baseline. (PNG) [file pone.0264674.s022.png]

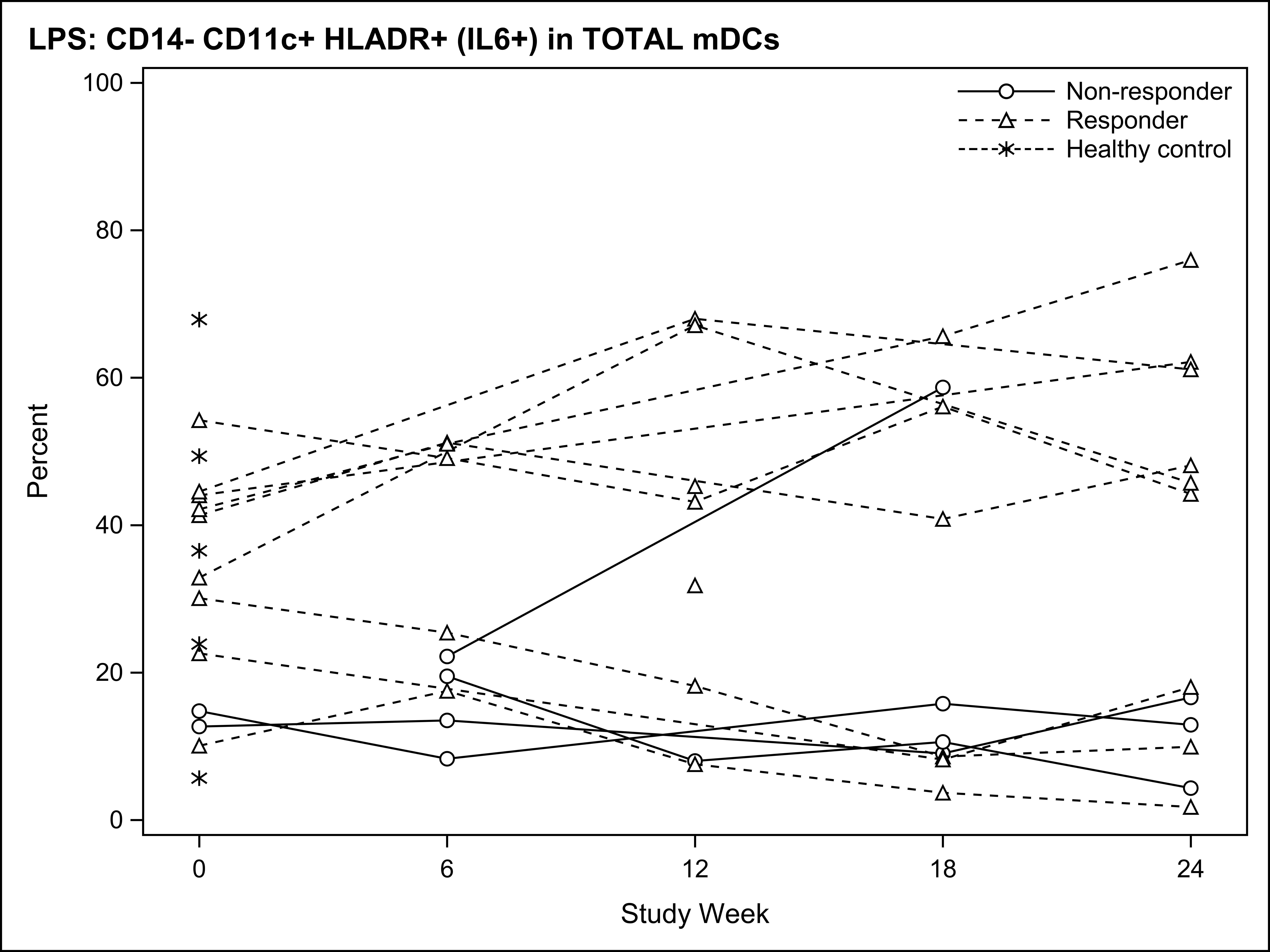

Supplement: S23 Fig — Changes in frequency of IL-6 expressing mDCs following LPS stimulation between responders (diamond) and non-responders (square) of peanut oral immunotherapy during the first 24-weeks of therapy. Healthy controls (circle) were not treated and only assessed at baseline. (PNG) [file pone.0264674.s023.png]

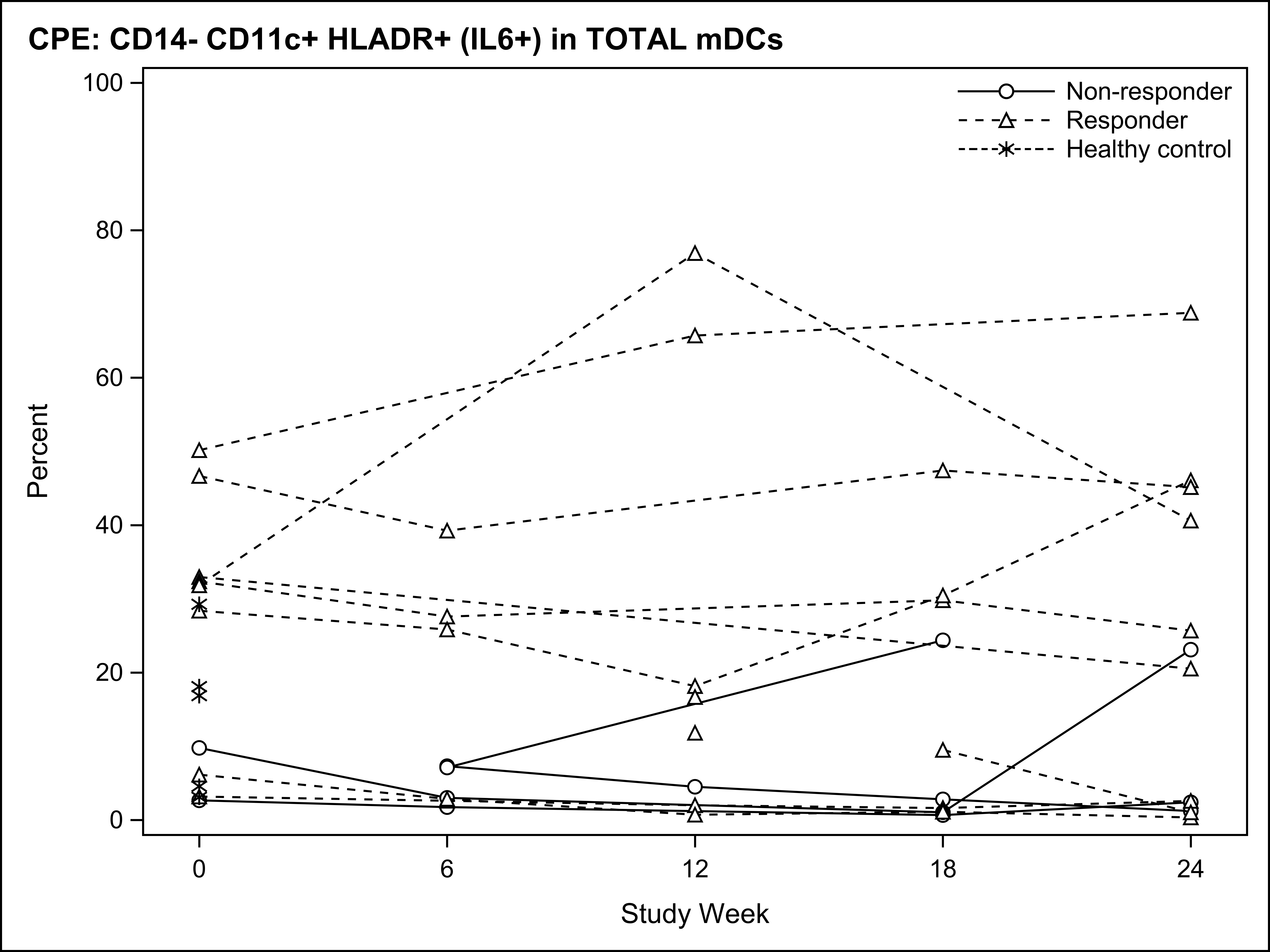

Supplement: S24 Fig — Changes in frequency of IL-6 expressing mDCs following CPE stimulation between responders (diamond) and non-responders (square) of peanut oral immunotherapy during the first 24-weeks of therapy. Healthy controls (circle) were not treated and only assessed at baseline. (PNG) [file pone.0264674.s024.png]

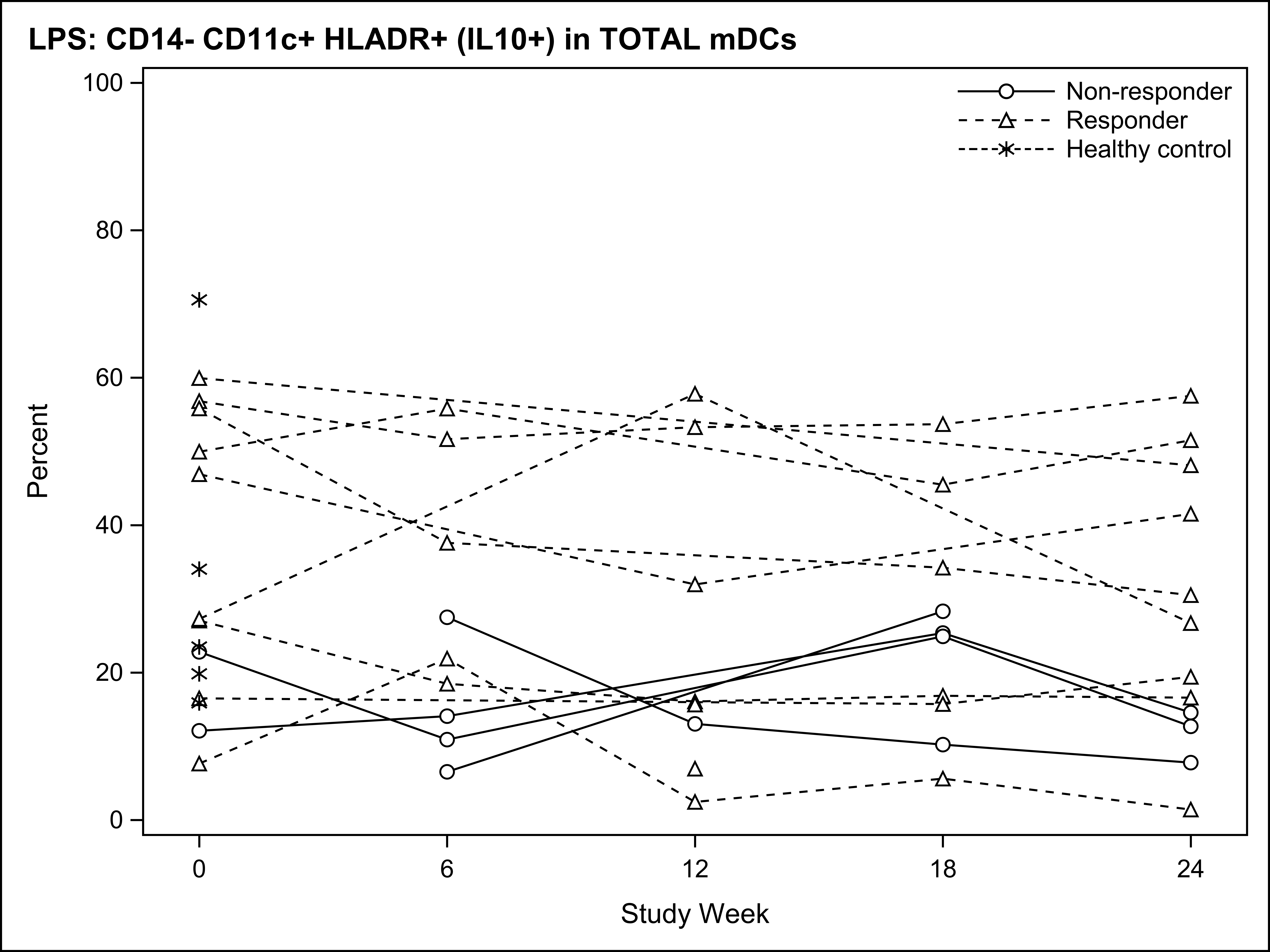

Supplement: S25 Fig — Changes in frequency of IL-10 expressing mDCs following LPS stimulation between responders (diamond) and non-responders (square) of peanut oral immunotherapy during the first 24-weeks of therapy. Healthy controls (circle) were not treated and only assessed at baseline. (PNG) [file pone.0264674.s025.png]

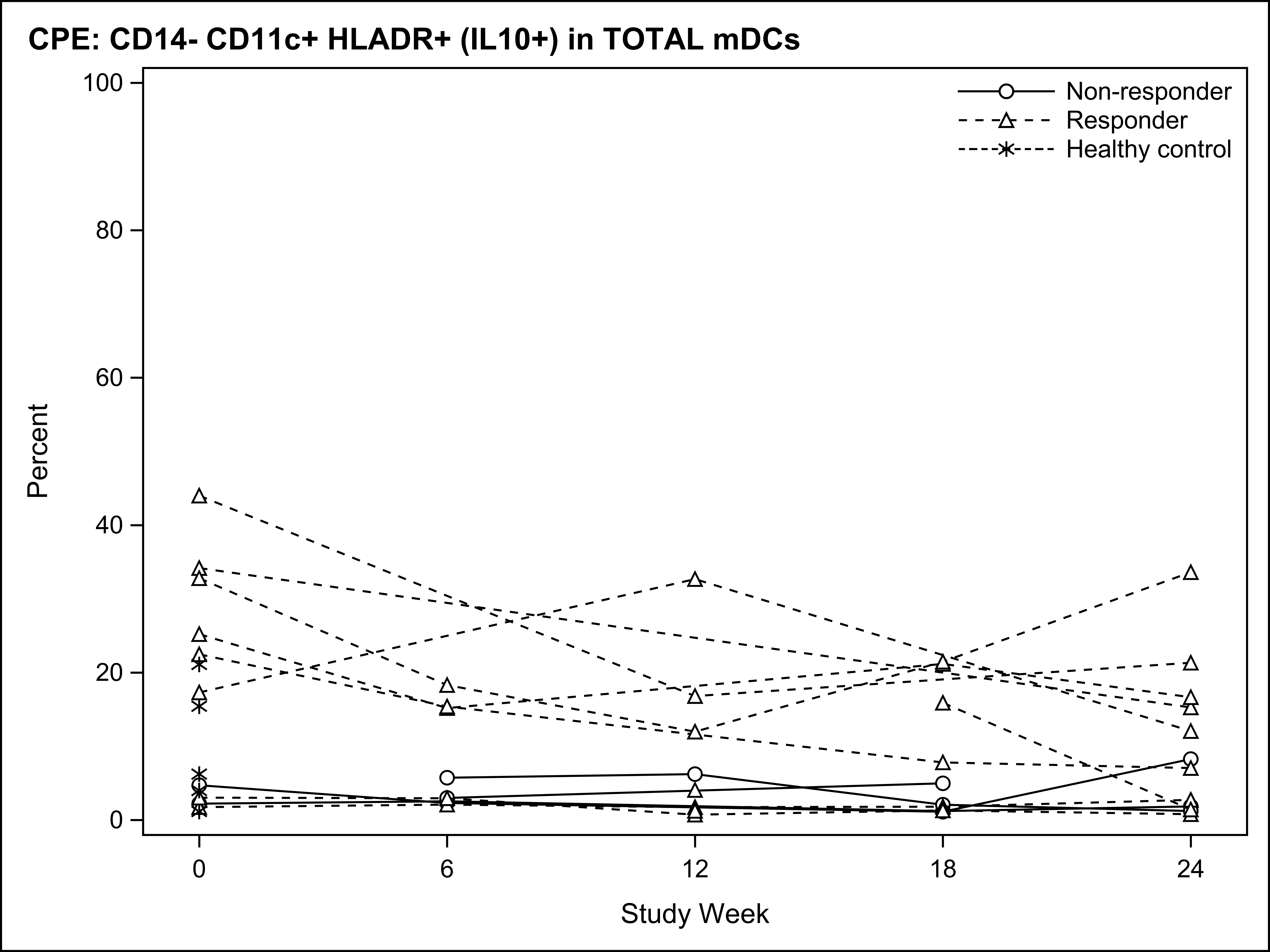

Supplement: S26 Fig — Changes in frequency of IL-10 expressing mDCs following CPE stimulation between responders (diamond) and non-responders (square) of peanut oral immunotherapy during the first 24-weeks of therapy. Healthy controls (circle) were not treated and only assessed at baseline. (PNG) [file pone.0264674.s026.png]

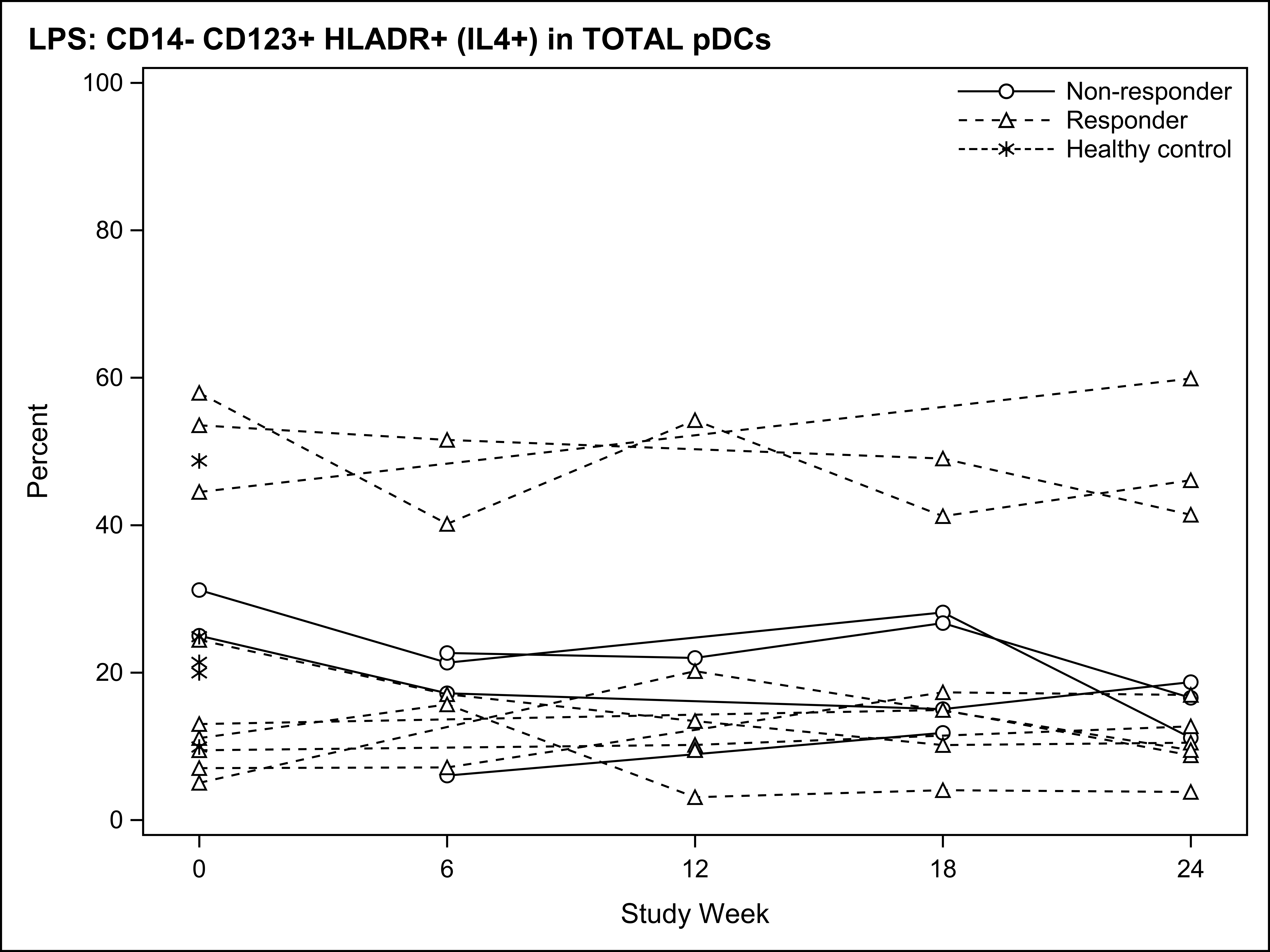

Supplement: S27 Fig — Changes in frequency of IL-4 expressing pDCs following LPS stimulation between responders (diamond) and non-responders (square) of peanut oral immunotherapy during the first 24-weeks of therapy. Healthy controls (circle) were not treated and only assessed at baseline. (PNG) [file pone.0264674.s027.png]

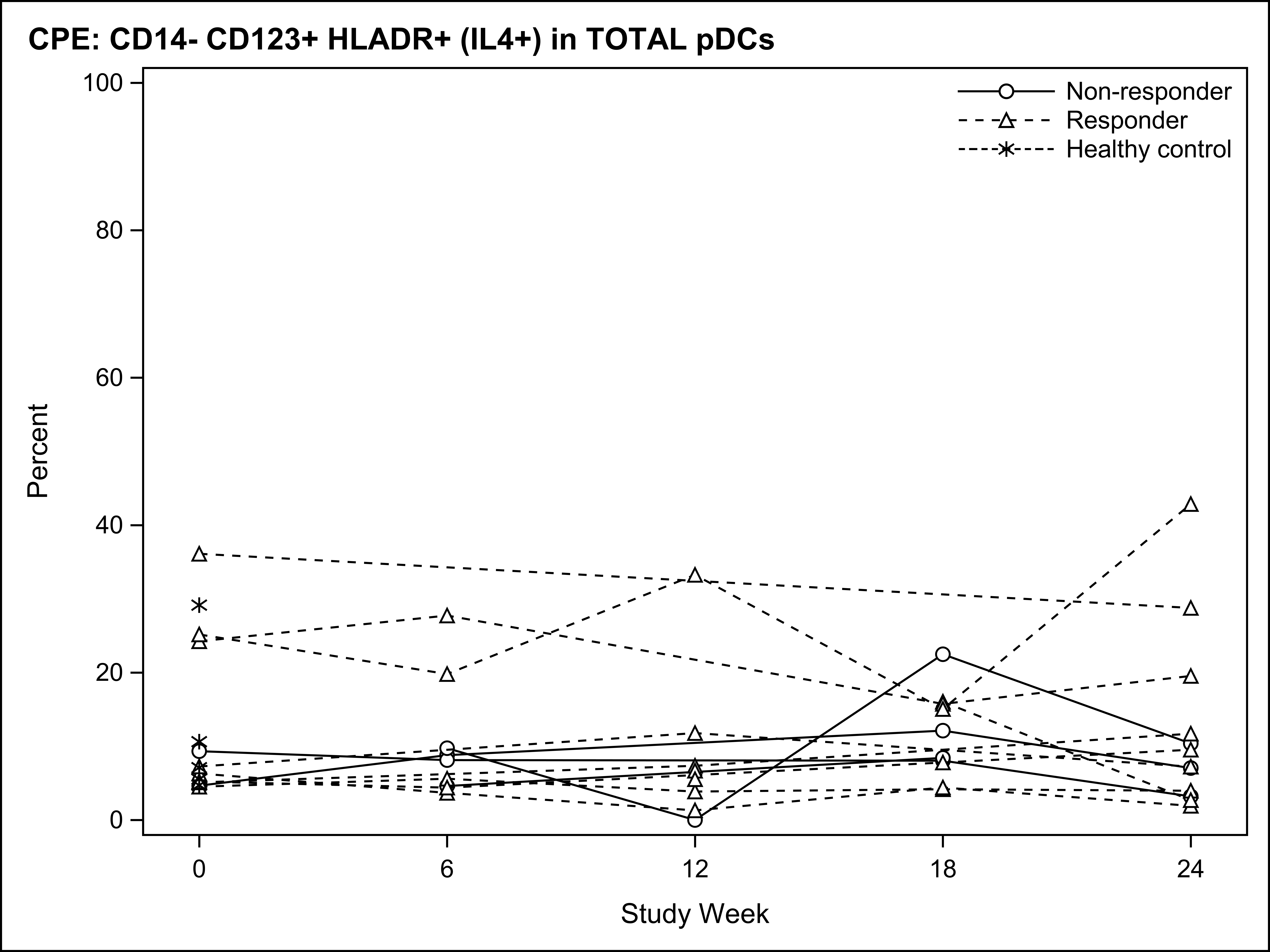

Supplement: S28 Fig — Changes in frequency of IL-4 expressing pDCs following CPE stimulation between responders (diamond) and non-responders (square) of peanut oral immunotherapy during the first 24-weeks of therapy. Healthy controls (circle) were not treated and only assessed at baseline. (PNG) [file pone.0264674.s028.png]

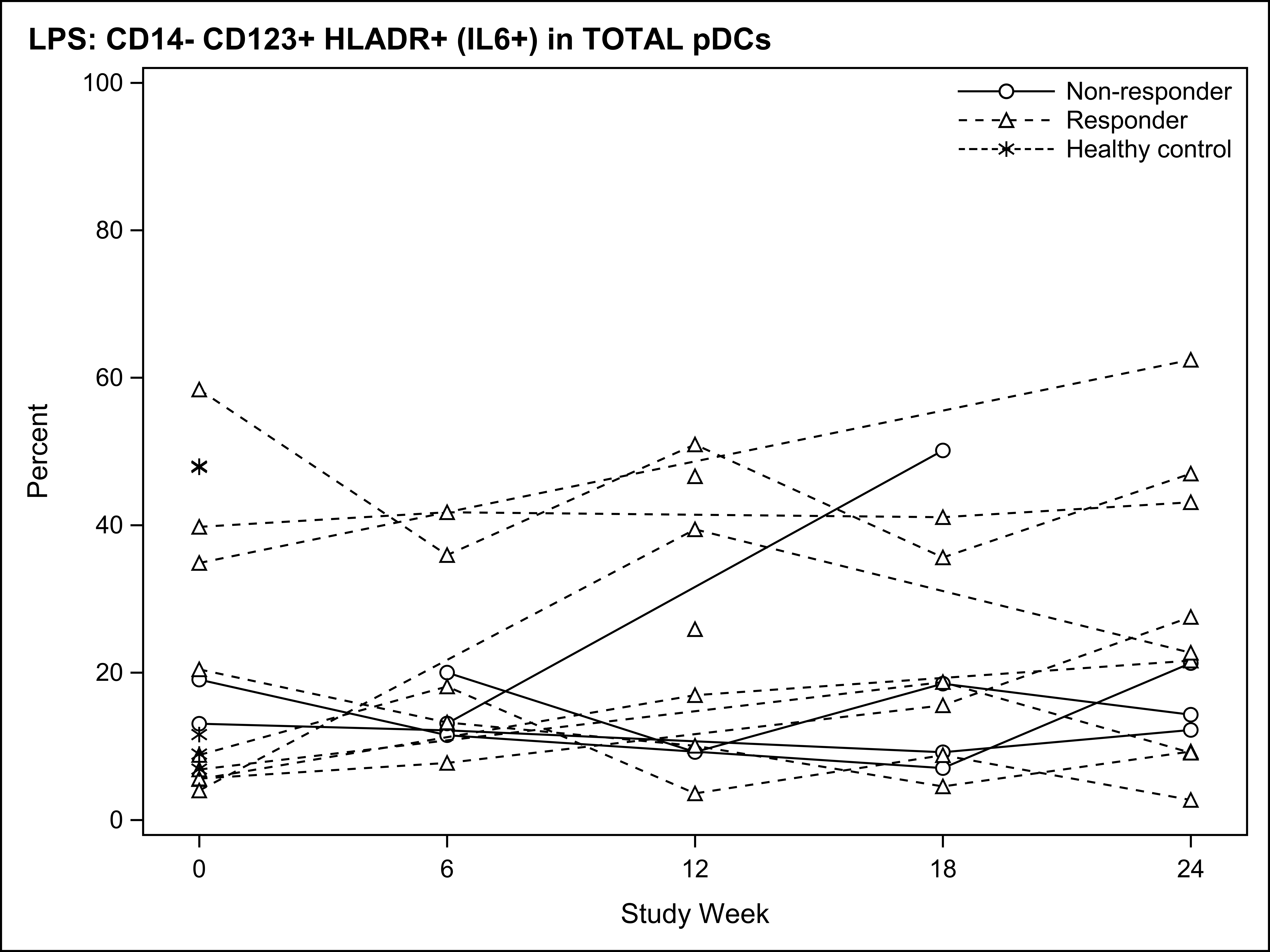

Supplement: S29 Fig — Changes in frequency of I6-4 expressing pDCs following LPS stimulation between responders (diamond) and non-responders (square) of peanut oral immunotherapy during the first 24-weeks of therapy. Healthy controls (circle) were not treated and only assessed at baseline. (PNG) [file pone.0264674.s029.png]

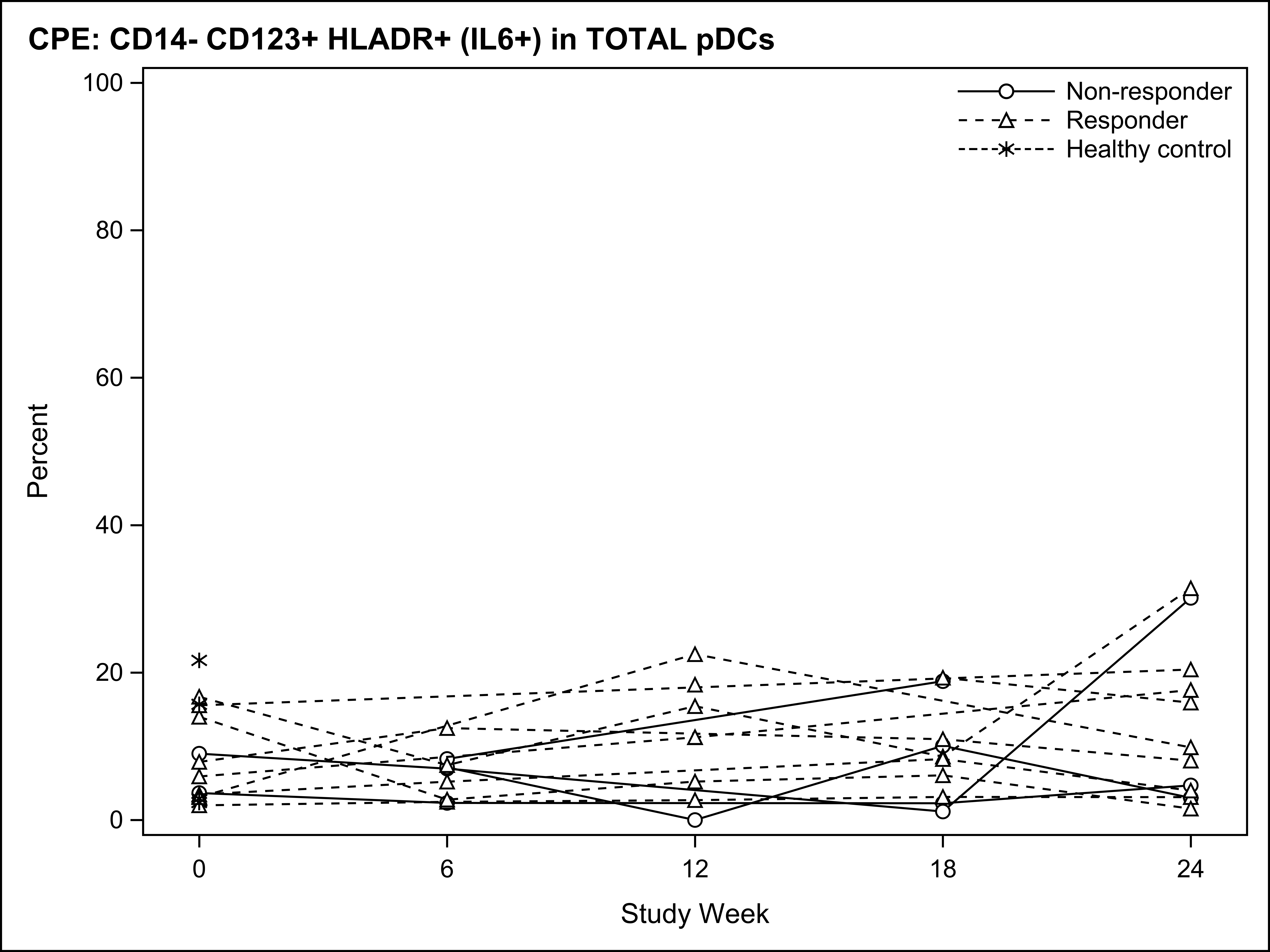

Supplement: S30 Fig — Changes in frequency of IL-6 expressing pDCs following CPE stimulation between responders (diamond) and non-responders (square) of peanut oral immunotherapy during the first 24-weeks of therapy. Healthy controls (circle) were not treated and only assessed at baseline. (PNG) [file pone.0264674.s030.png]

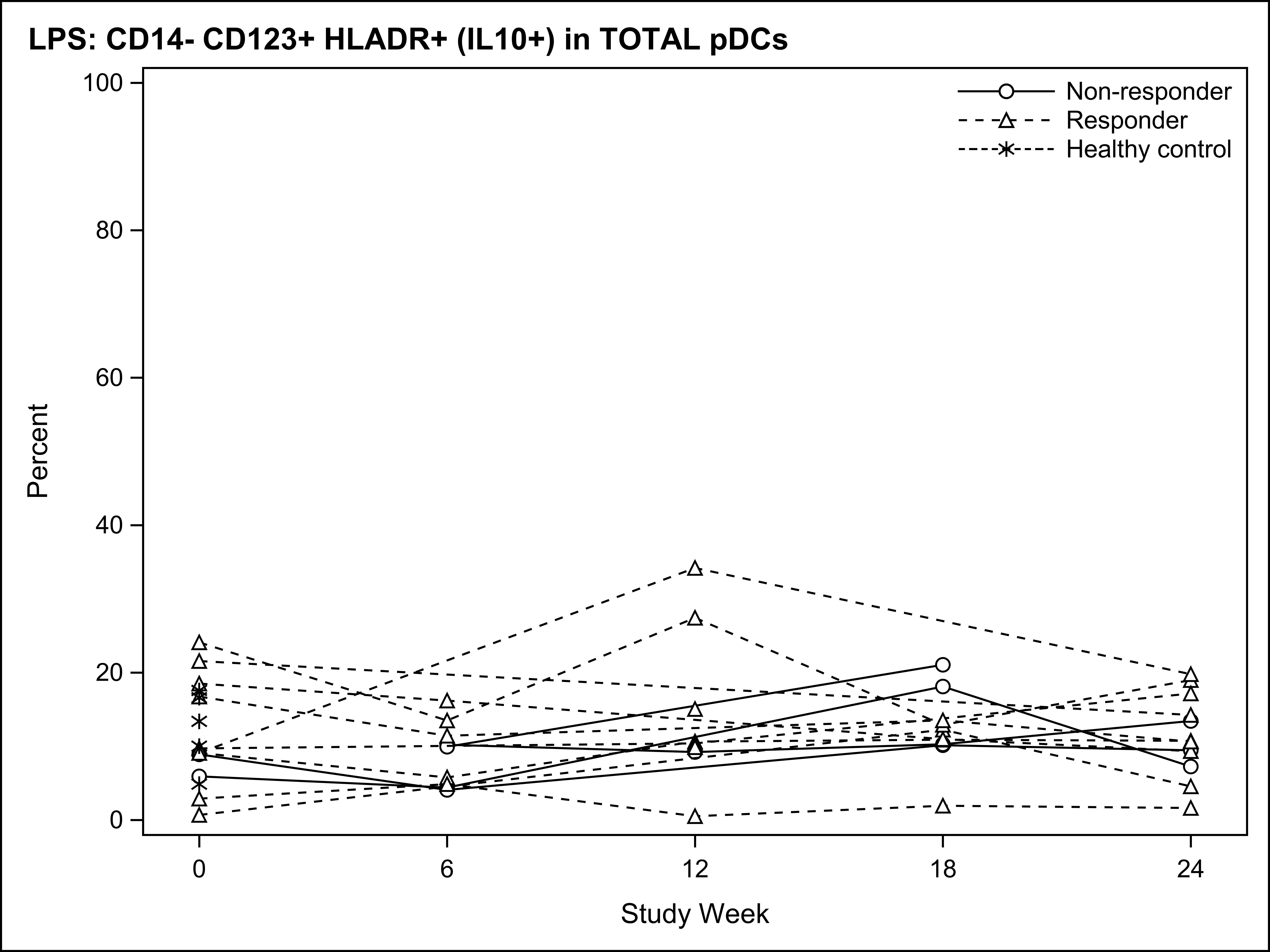

Supplement: S31 Fig — Changes in frequency of IL-10 expressing pDCs following LPS stimulation between responders (diamond) and non-responders (square) of peanut oral immunotherapy during the first 24-weeks of therapy. Healthy controls (circle) were not treated and only assessed at baseline. (PNG) [file pone.0264674.s031.png]

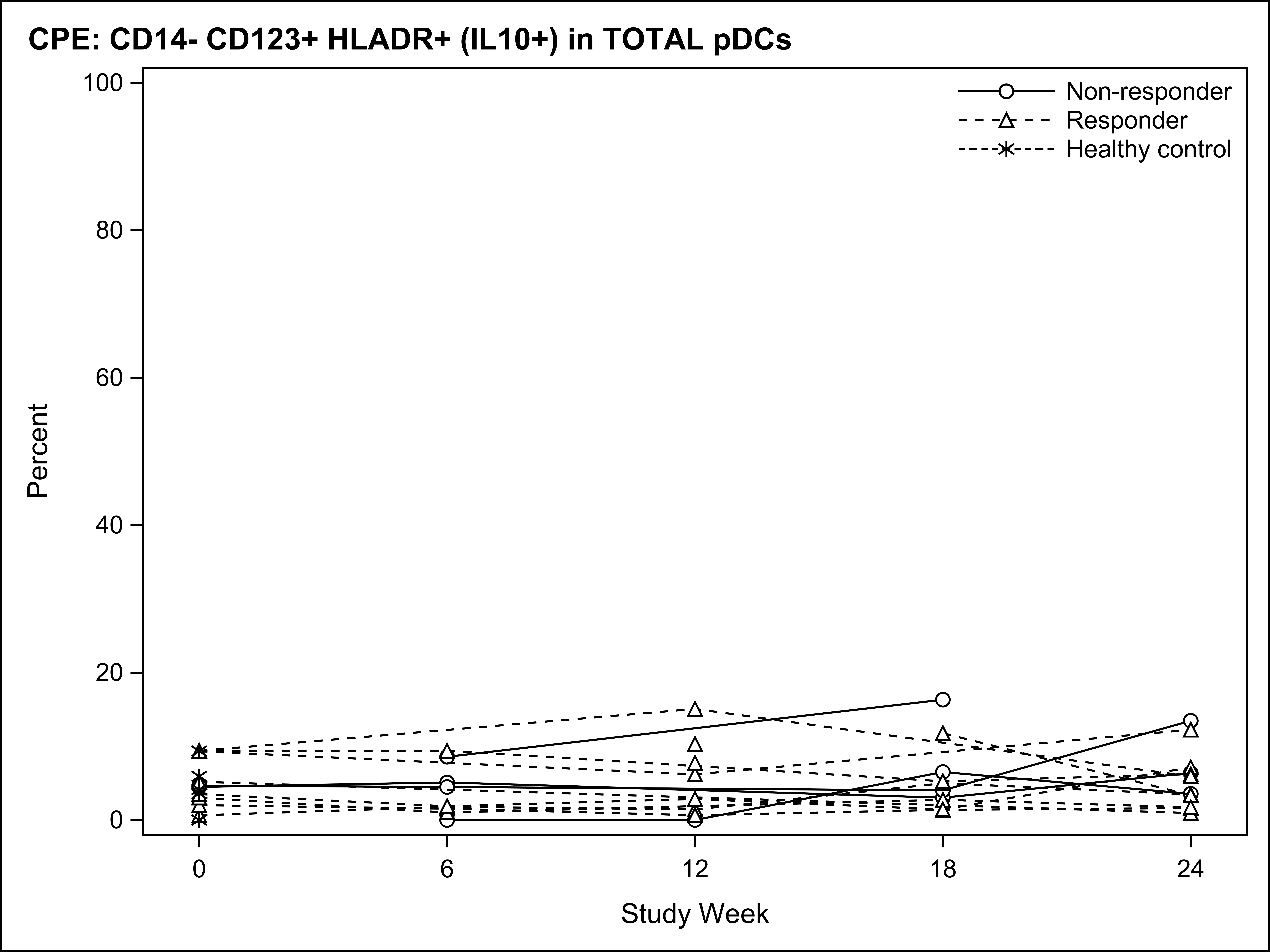

Supplement: S32 Fig — Changes in frequency of IL-10 expressing pDCs following CPE stimulation between responders (diamond) and non-responders (square) of peanut oral immunotherapy during the first 24-weeks of therapy. Healthy controls (circle) were not treated and only assessed at baseline. (PNG) [file pone.0264674.s032.png]

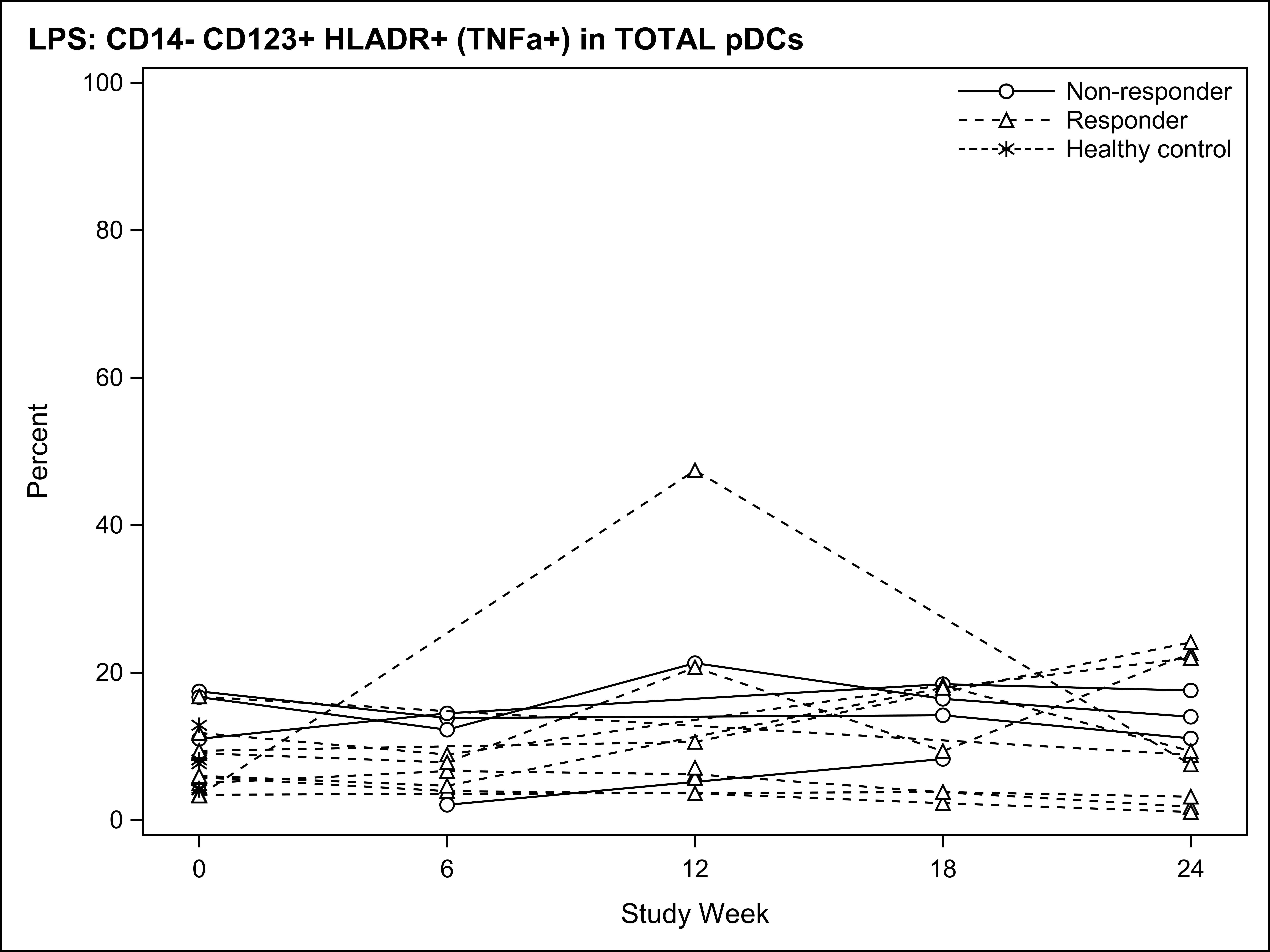

Supplement: S33 Fig — Changes in frequency of TNFa expressing pDCs following LPS stimulation between responders (diamond) and non-responders (square) of peanut oral immunotherapy during the first 24-weeks of therapy. Healthy controls (circle) were not treated and only assessed at baseline. (PNG) [file pone.0264674.s033.png]

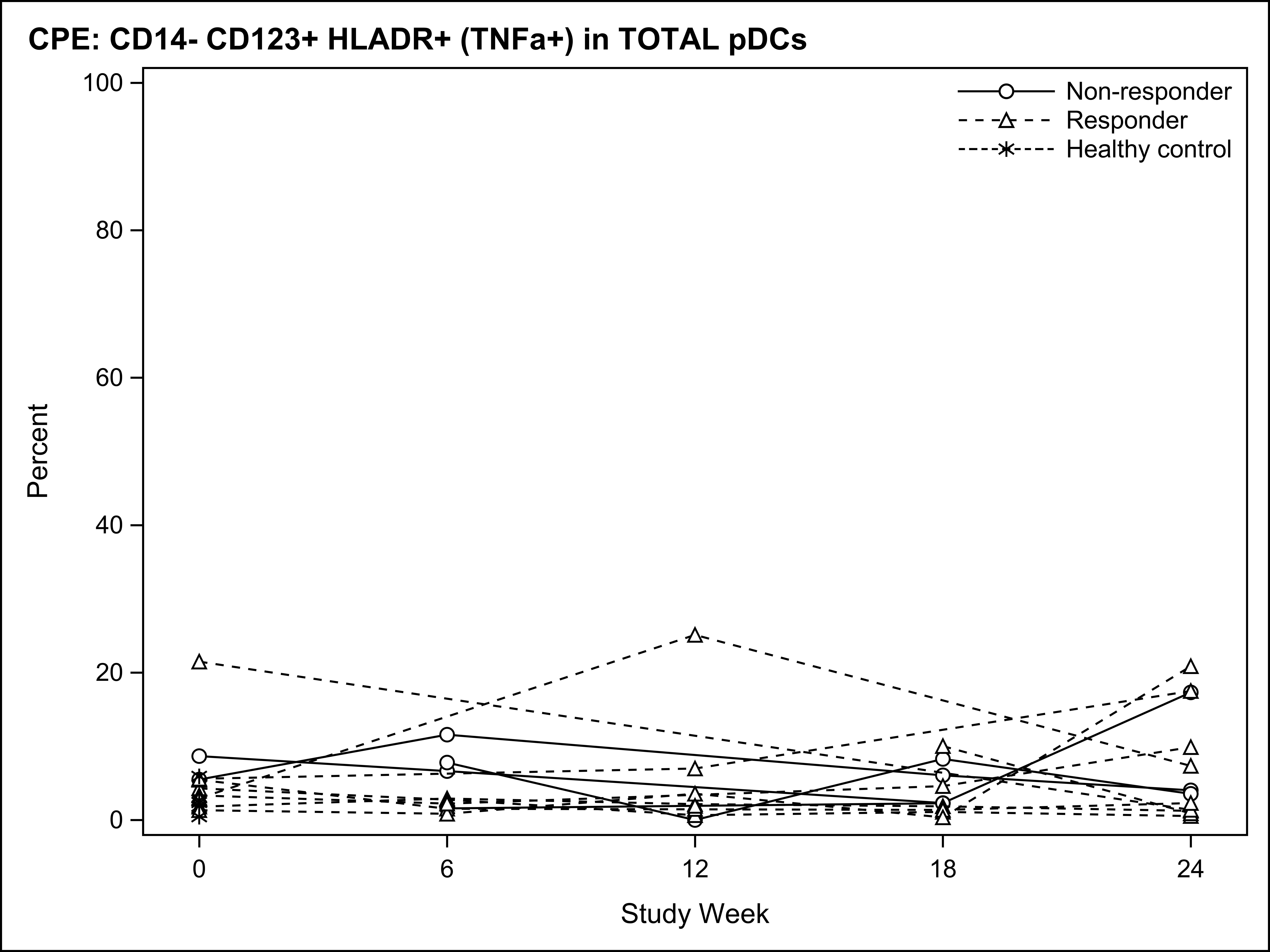

Supplement: S34 Fig — Changes in frequency of TNFa expressing pDCs following CPE stimulation between responders (diamond) and non-responders (square) of peanut oral immunotherapy during the first 24-weeks of therapy. Healthy controls (circle) were not treated and only assessed at baseline. (PNG) [file pone.0264674.s034.png]

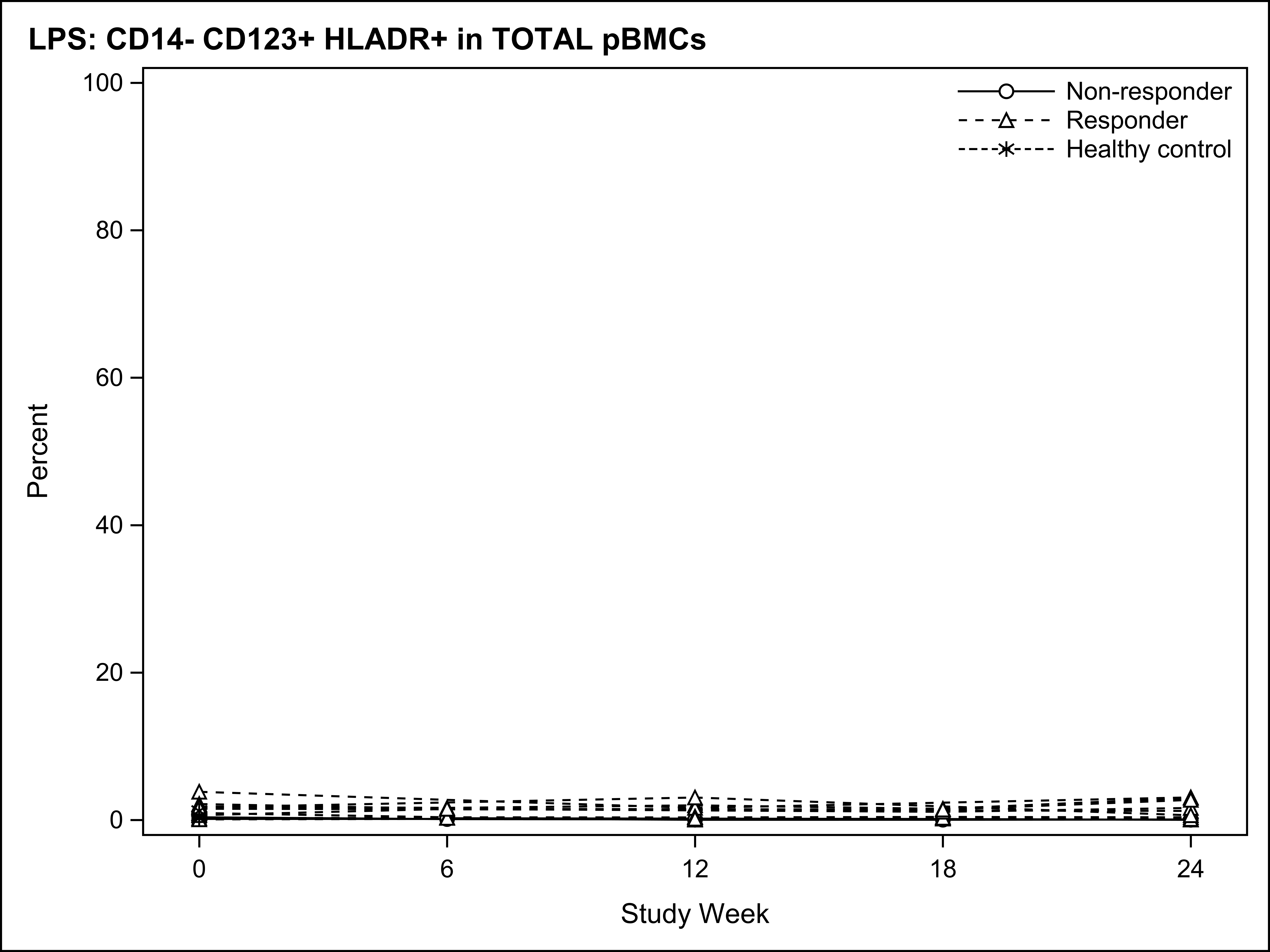

Supplement: S35 Fig — Changes in frequency of total mDCs following LPS stimulation between responders (diamond) and non-responders (square) of peanut oral immunotherapy during the first 24-weeks of therapy. Healthy controls (circle) were not treated and only assessed at baseline. (PNG) [file pone.0264674.s035.png]

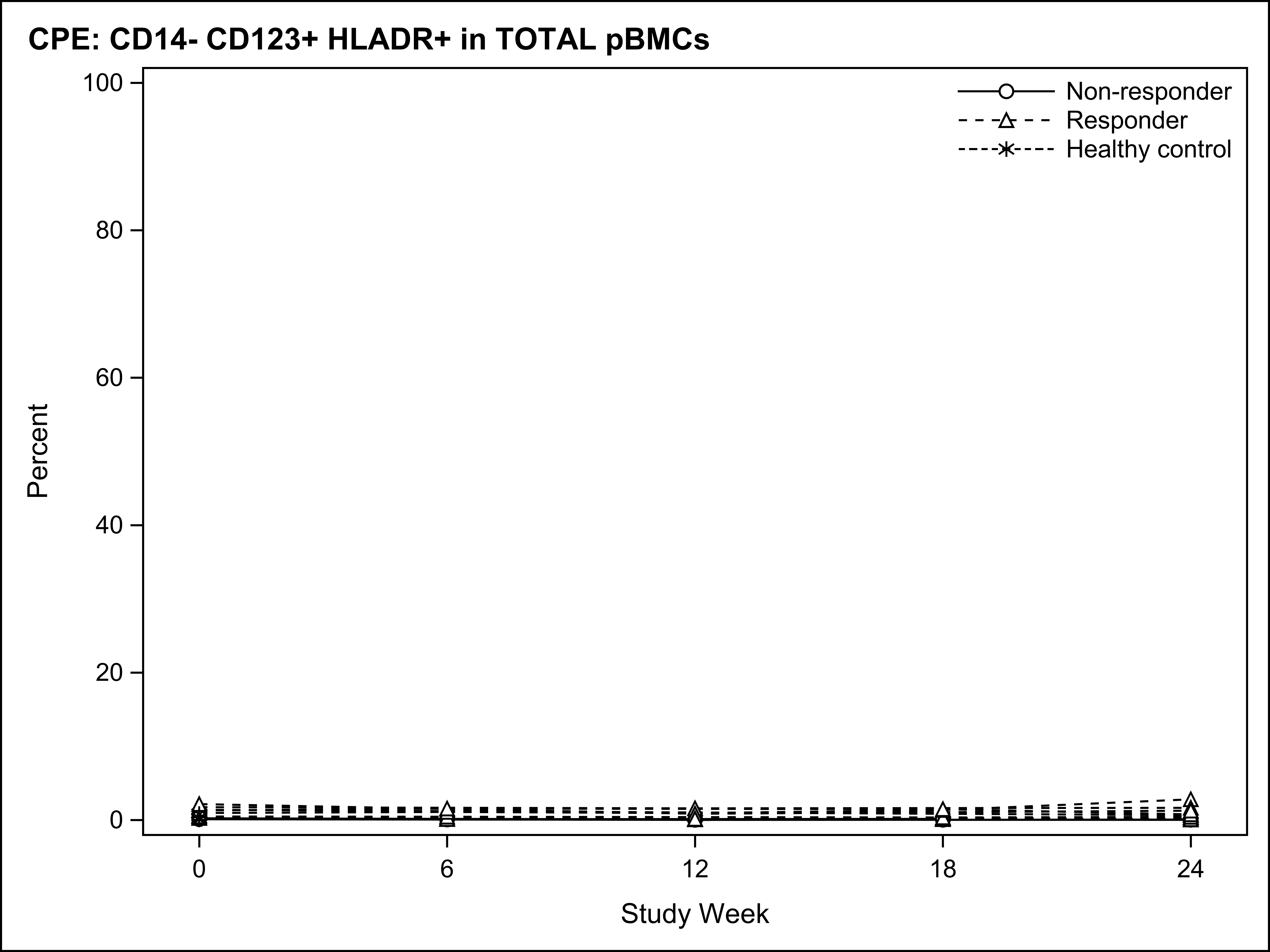

Supplement: S36 Fig — Changes in frequency of total mDCs following CPE stimulation between responders (diamond) and non-responders (square) of peanut oral immunotherapy during the first 24-weeks of therapy. Healthy controls (circle) were not treated and only assessed at baseline. (PNG) [file pone.0264674.s036.png]

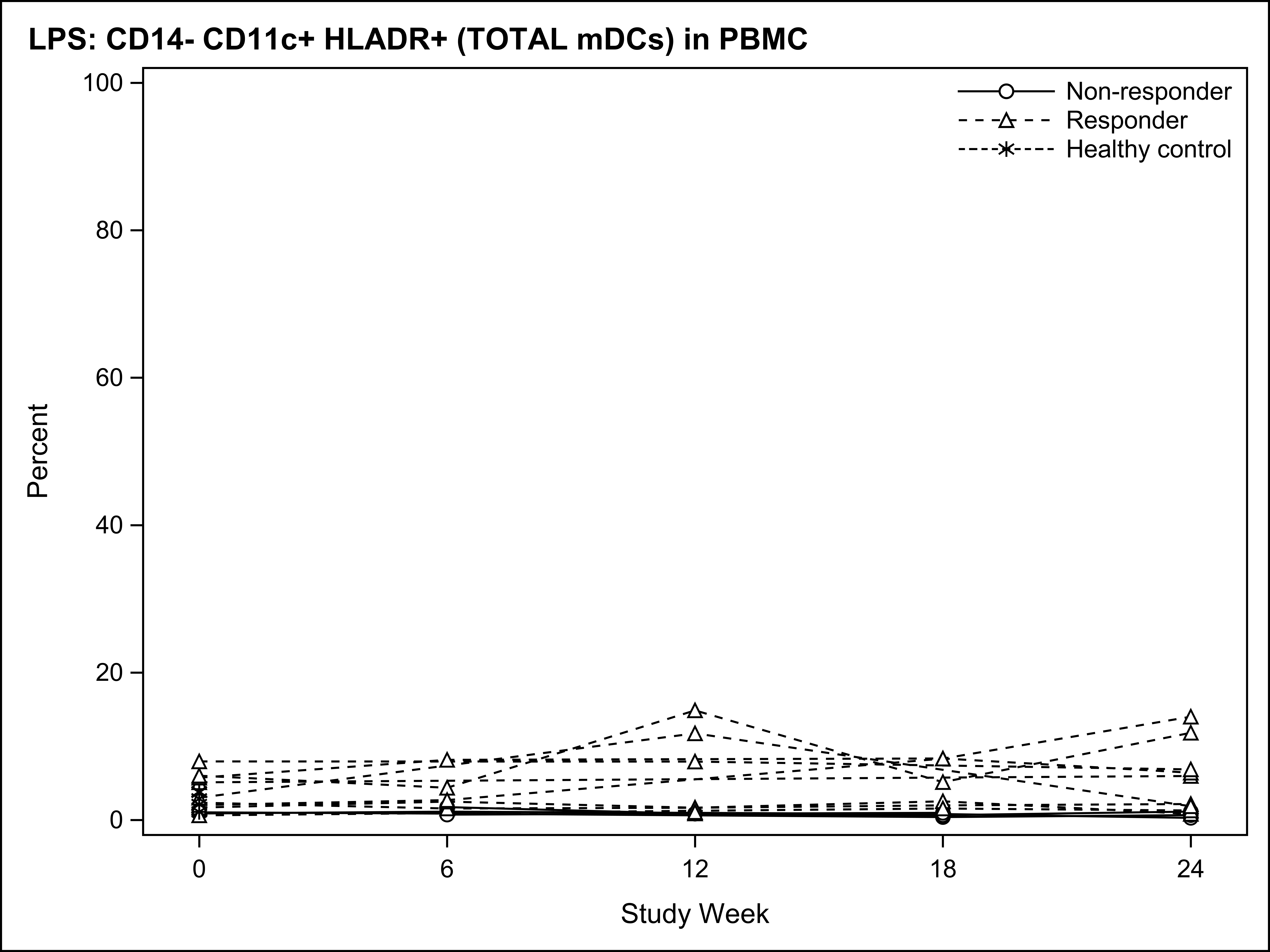

Supplement: S37 Fig — Changes in frequency of total pDCs following LPS stimulation between responders (diamond) and non-responders (square) of peanut oral immunotherapy during the first 24-weeks of therapy. Healthy controls (circle) were not treated and only assessed at baseline. (PNG) [file pone.0264674.s037.png]

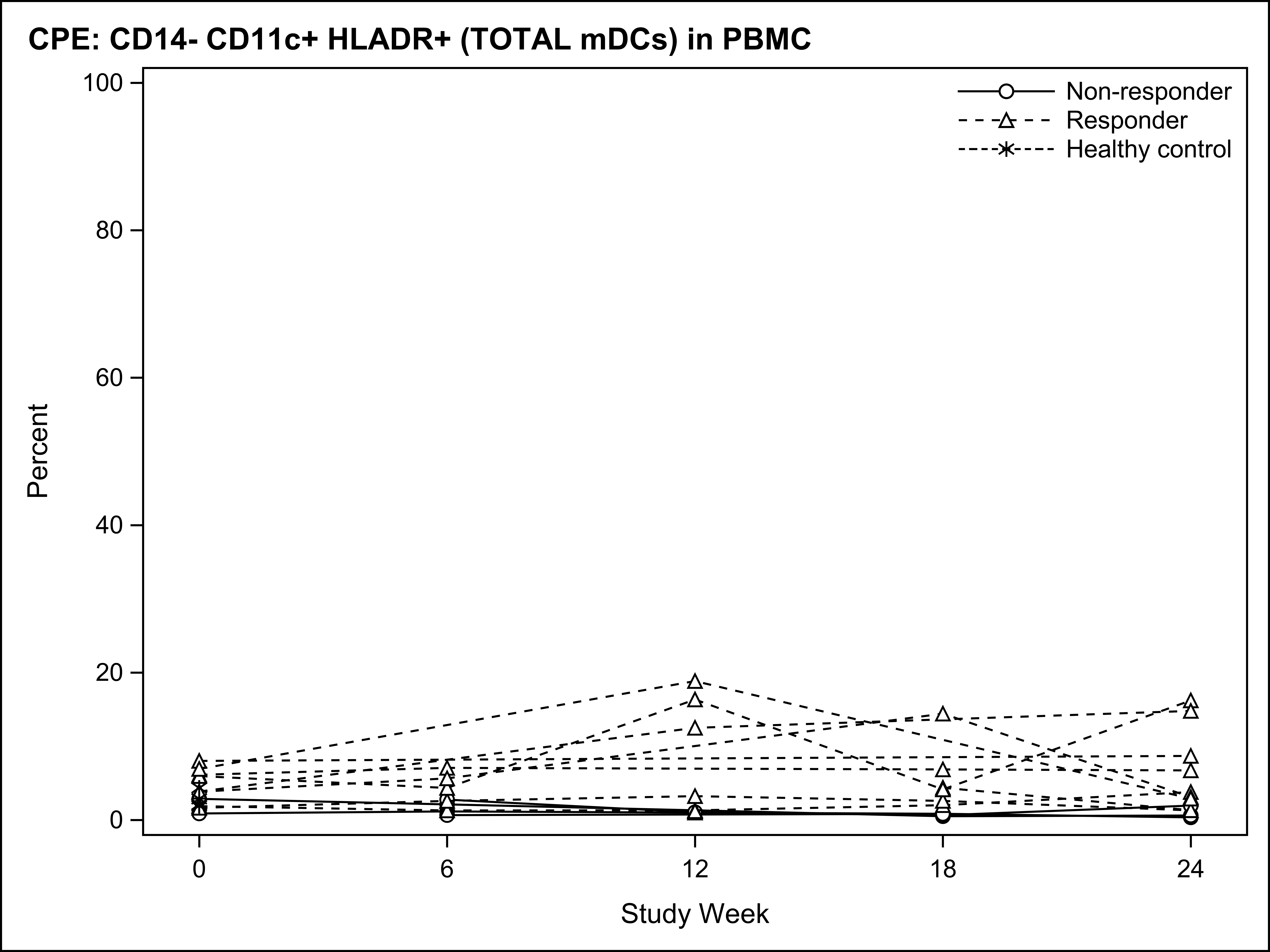

Supplement: S38 Fig — Changes in frequency of total pDCs following CPE stimulation between responders (diamond) and non-responders (square) of peanut oral immunotherapy during the first 24-weeks of therapy. Healthy controls (circle) were not treated and only assessed at baseline. (PNG) [file pone.0264674.s038.png]
