# Supplementary material for: Reduced pro-inflammatory dendritic cell phenotypes are a potential indicator of successful peanut oral immunotherapy
Source: PLoS One. 2022 May 26;17(5):e0264674. doi: 10.1371/journal.pone.0264674 (PMC9135258; doi:10.1371/journal.pone.0264674)
Supplement: S2 File — (RTF) [file pone.0264674.s040.rtf]

	response	
	N	Mean	StdDev	
Cell Type	Stimulant	StudyWeek	Responder Status	2	6.09	3.23	
CD14+ Monocytes in PBMC	CPE	0	Non-responder				
			Responder	8	16.14	9.11	
			Healthy control	5	14.48	11.39	
		6	Non-responder	4	6.56	3.37	
			Responder	5	19.61	15.07	
		12	Non-responder	1	1.63	.	
			Responder	7	18.04	15.19	
		18	Non-responder	4	3.14	1.24	
			Responder	7	23.17	8.27	
		24	Non-responder	3	2.98	1.08	
			Responder	9	21.50	11.21	
	LPS	0	Non-responder	2	3.02	0.79	
			Responder	9	9.32	5.20	
			Healthy control	5	8.22	8.37	
		6	Non-responder	4	3.66	1.90	
			Responder	5	8.01	3.83	
		12	Non-responder	1	1.25	.	
			Responder	7	11.71	15.99	
		18	Non-responder	4	2.98	2.02	
			Responder	6	13.82	7.46	
		24	Non-responder	3	3.37	3.10	
			Responder	9	9.90	6.11	
	NIL	0	Non-responder	2	7.18	3.60	
			Responder	9	10.71	8.80	
			Healthy control	5	18.84	31.95	
		6	Non-responder	4	6.97	3.27	
			Responder	5	19.03	15.75	
		12	Non-responder	1	2.73	.	
			Responder	7	11.61	14.45	
		18	Non-responder	4	3.53	1.14	
			Responder	6	17.56	10.16	
		24	Non-responder	3	3.49	1.54	
			Responder	9	15.67	12.79	
CD14+ CD11c+ HLADR+ Monocytes in PBMC	CPE	0	Non-responder	2	1.53	1.55	
			Responder	8	8.27	5.18	
			Healthy control	5	8.40	7.69	
		6	Non-responder	4	1.48	1.22	
			Responder	5	11.21	11.88	
		12	Non-responder	1	0.11	.	
			Responder	7	7.55	5.69	
		18	Non-responder	4	0.55	0.13	
			Responder	6	13.13	7.51	
		24	Non-responder	3	0.30	0.06	
			Responder	9	11.18	8.34	
	LPS	0	Non-responder	2	0.83	0.54	
			Responder	9	5.09	3.19	
			Healthy control	5	5.79	6.81	
		6	Non-responder	4	1.56	1.80	
			Responder	5	4.44	2.24	
		12	Non-responder	1	0.13	.	
			Responder	7	5.22	6.07	
		18	Non-responder	4	0.66	0.57	
			Responder	6	7.72	5.57	
		24	Non-responder	3	0.50	0.40	
			Responder	9	5.18	4.23	
	NIL	0	Non-responder	2	2.47	2.12	
			Responder	9	6.84	3.71	
			Healthy control	5	14.70	26.99	
		6	Non-responder	4	1.61	1.37	
			Responder	5	12.74	12.08	
		12	Non-responder	1	0.44	.	
			Responder	7	7.03	5.05	
		18	Non-responder	4	0.73	0.26	
			Responder	6	9.80	4.74	
		24	Non-responder	3	0.61	0.35	
			Responder	9	9.29	7.20	
CD14+ CD11c+ HLADR+ Monocytes in Total CD14 Monocytes	CPE	0	Non-responder	2	21.40	14.09	
			Responder	8	53.14	16.52	
			Healthy control	5	53.20	15.77	
		6	Non-responder	4	20.45	8.75	
			Responder	5	50.67	18.07	
		12	Non-responder	1	6.80	.	
			Responder	7	39.97	21.85	
		18	Non-responder	4	19.20	7.71	
			Responder	6	50.39	28.69	
		24	Non-responder	3	11.26	4.99	
			Responder	9	45.00	20.40	
	LPS	0	Non-responder	2	30.74	25.94	
			Responder	9	58.55	22.76	
			Healthy control	5	64.18	9.64	
		6	Non-responder	4	34.71	23.04	
			Responder	5	57.05	22.07	
		12	Non-responder	1	10.07	.	
			Responder	7	46.00	15.53	
		18	Non-responder	4	20.85	13.09	
			Responder	6	59.83	28.76	
		24	Non-responder	3	15.89	1.93	
			Responder	9	51.64	24.34	
	NIL	0	Non-responder	2	30.84	14.11	
			Responder	9	53.61	17.20	
			Healthy control	5	53.44	18.88	
		6	Non-responder	4	20.59	10.83	
			Responder	5	54.99	17.96	
		12	Non-responder	1	16.22	.	
			Responder	7	39.96	20.30	
		18	Non-responder	4	21.11	6.83	
			Responder	6	50.31	24.26	
		24	Non-responder	3	17.27	3.58	
			Responder	9	41.20	19.51	
CD14+ CD11c+ HLADR+ (CD80+) in PBMC	CPE	0	Non-responder	2	0.24	0.28	
			Responder	8	0.92	0.88	
			Healthy control	5	0.57	0.45	
		6	Non-responder	4	0.12	0.08	
			Responder	5	0.65	0.31	
		12	Non-responder	1	0.02	.	
			Responder	7	0.46	0.47	
		18	Non-responder	4	0.05	0.02	
			Responder	6	1.83	2.54	
		24	Non-responder	3	0.04	0.02	
			Responder	9	1.17	1.68	
	LPS	0	Non-responder	2	0.18	0.13	
			Responder	9	1.10	1.29	
			Healthy control	5	1.01	1.37	
		6	Non-responder	4	0.25	0.25	
			Responder	5	1.17	0.84	
		12	Non-responder	1	0.02	.	
			Responder	7	0.66	0.78	
		18	Non-responder	4	0.11	0.09	
			Responder	6	1.61	0.94	
		24	Non-responder	3	0.09	0.06	
			Responder	9	1.51	2.24	
	NIL	0	Non-responder	2	0.45	0.53	
			Responder	9	0.76	0.89	
			Healthy control	5	1.20	2.03	
		6	Non-responder	4	0.14	0.12	
			Responder	5	0.89	0.92	
		12	Non-responder	1	0.03	.	
			Responder	7	0.47	0.55	
		18	Non-responder	4	0.07	0.04	
			Responder	6	1.35	1.35	
		24	Non-responder	3	0.09	0.08	
			Responder	9	0.74	0.88	
CD14+ CD11c+ HLADR+ (CD80+) in TOTAL CD14 Monocytes	CPE	0	Non-responder	2	12.99	5.08	
			Responder	8	9.58	3.86	
			Healthy control	5	8.14	3.76	
		6	Non-responder	4	8.40	3.08	
			Responder	5	8.59	4.79	
		12	Non-responder	1	16.33	.	
			Responder	7	7.75	4.86	
		18	Non-responder	4	8.81	3.15	
			Responder	6	11.33	10.32	
		24	Non-responder	3	11.18	3.24	
			Responder	9	8.82	8.07	
	LPS	0	Non-responder	2	20.35	2.59	
			Responder	9	17.55	12.97	
			Healthy control	5	14.41	7.60	
		6	Non-responder	4	16.99	5.99	
			Responder	5	23.04	13.29	
		12	Non-responder	1	12.13	.	
			Responder	7	14.68	13.72	
		18	Non-responder	4	18.38	4.80	
			Responder	6	23.49	15.84	
		24	Non-responder	3	17.69	2.10	
			Responder	9	21.27	20.30	
	NIL	0	Non-responder	2	14.05	9.29	
			Responder	9	9.25	7.07	
			Healthy control	5	10.36	4.05	
		6	Non-responder	4	9.00	3.84	
			Responder	5	8.91	6.57	
		12	Non-responder	1	7.08	.	
			Responder	7	8.83	7.22	
		18	Non-responder	4	9.72	4.85	
			Responder	6	12.83	12.66	
		24	Non-responder	3	13.08	6.74	
			Responder	9	6.94	3.50	
CD14+ CD11c+ HLADR+ (CD83+) in PBMC	CPE	0	Non-responder	2	0.63	0.71	
			Responder	8	3.77	3.69	
			Healthy control	5	2.15	3.09	
		6	Non-responder	4	0.41	0.43	
			Responder	5	4.92	6.74	
		12	Non-responder	1	0.03	.	
			Responder	7	2.62	2.73	
		18	Non-responder	4	0.14	0.11	
			Responder	6	4.75	3.97	
		24	Non-responder	3	0.13	0.04	
			Responder	9	4.44	4.61	
	LPS	0	Non-responder	2	0.50	0.28	
			Responder	9	3.60	2.91	
			Healthy control	5	2.96	3.66	
		6	Non-responder	4	0.69	0.78	
			Responder	5	3.26	1.86	
		12	Non-responder	1	0.06	.	
			Responder	7	2.63	2.95	
		18	Non-responder	4	0.41	0.47	
			Responder	6	4.96	3.32	
		24	Non-responder	3	0.36	0.29	
			Responder	9	3.66	3.58	
	NIL	0	Non-responder	2	1.24	1.24	
			Responder	9	2.95	2.27	
			Healthy control	5	2.03	3.53	
		6	Non-responder	4	0.56	0.59	
			Responder	5	5.95	6.91	
		12	Non-responder	1	0.24	.	
			Responder	7	2.58	2.25	
		18	Non-responder	4	0.28	0.20	
			Responder	6	3.93	2.41	
		24	Non-responder	3	0.38	0.26	
			Responder	9	4.13	4.12	
CD14+ CD11c+ HLADR+ (CD83+) in TOTAL CD14 Monocytes	CPE	0	Non-responder	2	36.24	9.42	
			Responder	8	38.25	18.02	
			Healthy control	5	21.66	13.99	
		6	Non-responder	4	21.68	15.26	
			Responder	5	35.95	20.72	
		12	Non-responder	1	30.61	.	
			Responder	7	27.18	26.56	
		18	Non-responder	4	26.07	21.53	
			Responder	6	36.16	17.89	
		24	Non-responder	3	43.34	8.36	
			Responder	9	34.07	20.59	
	LPS	0	Non-responder	2	63.43	7.45	
			Responder	9	64.38	22.58	
			Healthy control	5	45.14	26.01	
		6	Non-responder	4	39.58	28.38	
			Responder	5	66.47	22.99	
		12	Non-responder	1	51.33	.	
			Responder	7	41.87	31.87	
		18	Non-responder	4	51.10	35.24	
			Responder	6	65.09	15.70	
		24	Non-responder	3	72.50	13.11	
			Responder	9	65.47	17.57	
	NIL	0	Non-responder	2	45.50	11.25	
			Responder	9	40.65	15.69	
			Healthy control	5	19.87	13.39	
		6	Non-responder	4	26.23	17.89	
			Responder	5	41.65	17.32	
		12	Non-responder	1	53.46	.	
			Responder	7	29.26	23.10	
		18	Non-responder	4	34.76	25.03	
			Responder	6	39.86	11.68	
		24	Non-responder	3	59.08	6.96	
			Responder	9	39.47	16.11	
CD14+ CD11c+ HLADR+ (IL4+) in PBMC	CPE	0	Non-responder	2	0.39	0.45	
			Responder	8	3.28	3.86	
			Healthy control	5	1.68	2.94	
		6	Non-responder	4	0.27	0.30	
			Responder	5	2.58	3.83	
		12	Non-responder	1	0.04	.	
			Responder	7	1.39	2.35	
		18	Non-responder	4	0.17	0.11	
			Responder	6	2.63	2.83	
		24	Non-responder	3	0.10	0.04	
			Responder	9	2.52	3.94	
	LPS	0	Non-responder	2	0.28	0.11	
			Responder	9	2.19	2.96	
			Healthy control	5	1.13	1.65	
		6	Non-responder	4	0.12	0.10	
			Responder	5	1.85	1.95	
		12	Non-responder	1	0.03	.	
			Responder	7	0.90	1.60	
		18	Non-responder	4	0.23	0.30	
			Responder	6	1.46	1.49	
		24	Non-responder	3	0.13	0.08	
			Responder	9	1.70	3.04	
	NIL	0	Non-responder	2	1.02	1.05	
			Responder	9	2.18	2.78	
			Healthy control	5	0.57	0.46	
		6	Non-responder	4	0.40	0.33	
			Responder	5	2.15	2.62	
		12	Non-responder	1	0.30	.	
			Responder	7	1.19	1.80	
		18	Non-responder	4	0.24	0.16	
			Responder	6	2.05	2.20	
		24	Non-responder	3	0.14	0.09	
			Responder	9	2.06	3.17	
CD14+ CD11c+ HLADR+ (IL4+) in TOTAL CD14 Monocytes	CPE	0	Non-responder	2	21.34	7.92	
			Responder	8	34.48	24.13	
			Healthy control	5	19.21	16.92	
		6	Non-responder	4	13.97	9.27	
			Responder	5	22.94	20.87	
		12	Non-responder	1	38.78	.	
			Responder	7	14.66	22.50	
		18	Non-responder	4	30.79	20.32	
			Responder	6	21.42	16.72	
		24	Non-responder	3	32.09	14.40	
			Responder	9	21.04	21.42	
	LPS	0	Non-responder	2	38.00	11.73	
			Responder	9	39.93	34.19	
			Healthy control	5	27.11	30.50	
		6	Non-responder	4	10.60	9.96	
			Responder	5	41.90	34.03	
		12	Non-responder	1	27.41	.	
			Responder	7	19.74	30.72	
		18	Non-responder	4	27.00	20.45	
			Responder	6	34.54	34.64	
		24	Non-responder	3	28.88	14.25	
			Responder	9	30.23	31.41	
	NIL	0	Non-responder	2	36.81	10.89	
			Responder	9	30.40	25.68	
			Healthy control	5	15.63	13.17	
		6	Non-responder	4	24.04	17.86	
			Responder	5	22.38	19.02	
		12	Non-responder	1	66.71	.	
			Responder	7	15.08	22.80	
		18	Non-responder	4	29.96	19.52	
			Responder	6	21.86	17.46	
		24	Non-responder	3	21.71	7.34	
			Responder	9	16.10	17.23	
CD14+ CD11c+ HLADR+ (IL6+) in PBMC	CPE	0	Non-responder	2	0.23	0.26	
			Responder	8	1.36	1.23	
			Healthy control	5	1.13	1.74	
		6	Non-responder	4	0.12	0.10	
			Responder	5	1.78	1.31	
		12	Non-responder	1	0.01	.	
			Responder	7	0.77	0.96	
		18	Non-responder	4	0.04	0.04	
			Responder	6	3.55	4.12	
		24	Non-responder	3	0.03	0.01	
			Responder	9	2.28	2.87	
	LPS	0	Non-responder	2	0.36	0.25	
			Responder	9	3.04	3.21	
			Healthy control	5	3.12	5.16	
		6	Non-responder	4	0.41	0.43	
			Responder	5	2.69	2.14	
		12	Non-responder	1	0.05	.	
			Responder	7	2.36	3.53	
		18	Non-responder	4	0.27	0.38	
			Responder	6	5.13	4.87	
		24	Non-responder	3	0.21	0.14	
			Responder	9	3.00	3.90	
	NIL	0	Non-responder	2	0.95	1.04	
			Responder	9	2.38	2.43	
			Healthy control	5	3.99	8.61	
		6	Non-responder	4	0.45	0.47	
			Responder	5	3.50	3.16	
		12	Non-responder	1	0.18	.	
			Responder	7	1.22	1.73	
		18	Non-responder	4	0.11	0.08	
			Responder	6	3.80	2.76	
		24	Non-responder	3	0.20	0.17	
			Responder	9	2.25	2.50	
CD14+ CD11c+ HLADR+ (IL6+) in TOTAL CD14 Monocytes	CPE	0	Non-responder	3	8.25	7.85	
			Responder	8	13.36	11.85	
			Healthy control	5	8.00	12.11	
		6	Non-responder	4	6.85	4.11	
			Responder	5	17.16	13.66	
		12	Non-responder	1	6.12	.	
			Responder	7	7.96	9.81	
		18	Non-responder	4	6.97	8.94	
			Responder	6	21.26	17.88	
		24	Non-responder	3	11.53	5.05	
			Responder	9	13.48	14.62	
	LPS	0	Non-responder	2	43.53	1.30	
			Responder	9	47.58	35.06	
			Healthy control	5	31.51	26.59	
		6	Non-responder	4	26.34	13.77	
			Responder	5	50.77	35.86	
		12	Non-responder	1	38.21	.	
			Responder	7	34.99	31.79	
		18	Non-responder	4	32.72	19.58	
			Responder	6	52.67	31.26	
		24	Non-responder	3	45.18	9.70	
			Responder	9	41.91	31.91	
	NIL	0	Non-responder	2	32.64	14.08	
			Responder	9	25.07	22.24	
			Healthy control	5	10.09	11.98	
		6	Non-responder	4	21.71	13.90	
			Responder	7	20.08	22.85	
		12	Non-responder	1	41.17	.	
			Responder	7	13.51	15.97	
		18	Non-responder	4	14.30	13.18	
			Responder	6	34.78	23.48	
		24	Non-responder	3	29.23	9.72	
			Responder	9	19.00	19.60	
CD14+ CD11c+ HLADR+ (IL10+) in PBMC	CPE	0	Non-responder	2	0.16	0.16	
			Responder	8	2.82	3.23	
			Healthy control	5	1.83	3.24	
		6	Non-responder	4	0.23	0.19	
			Responder	5	2.32	3.74	
		12	Non-responder	1	0.01	.	
			Responder	7	1.03	1.39	
		18	Non-responder	4	0.05	0.04	
			Responder	6	1.95	2.52	
		24	Non-responder	3	0.03	0.02	
			Responder	9	2.12	2.97	
	LPS	0	Non-responder	2	0.52	0.37	
			Responder	9	3.20	2.67	
			Healthy control	5	2.51	1.87	
		6	Non-responder	4	0.87	1.04	
			Responder	5	2.75	1.55	
		12	Non-responder	1	0.06	.	
			Responder	7	1.57	1.52	
		18	Non-responder	4	0.45	0.48	
			Responder	6	2.92	1.17	
		24	Non-responder	3	0.28	0.25	
			Responder	9	2.73	2.89	
	NIL	0	Non-responder	2	0.78	0.79	
			Responder	9	2.08	2.39	
			Healthy control	5	0.72	0.62	
		6	Non-responder	4	0.66	0.67	
			Responder	5	1.85	2.34	
		12	Non-responder	1	0.21	.	
			Responder	7	1.47	1.96	
		18	Non-responder	4	0.12	0.05	
			Responder	6	1.59	2.20	
		24	Non-responder	3	0.15	0.13	
			Responder	9	1.85	2.44	
CD14+ CD11c+ HLADR+ (IL10+) in TOTAL CD14 Monocytes	CPE	0	Non-responder	2	10.34	0.13	
			Responder	8	33.93	24.97	
			Healthy control	5	16.86	15.38	
		6	Non-responder	4	16.37	3.82	
			Responder	5	17.30	14.73	
		12	Non-responder	1	12.24	.	
			Responder	7	13.79	12.77	
		18	Non-responder	4	10.79	9.78	
			Responder	6	14.44	13.71	
		24	Non-responder	3	8.24	4.41	
			Responder	9	16.24	17.89	
	LPS	0	Non-responder	2	61.56	4.71	
			Responder	9	62.32	24.46	
			Healthy control	5	56.96	22.29	
		6	Non-responder	4	50.61	15.96	
			Responder	5	62.96	15.42	
		12	Non-responder	1	45.35	.	
			Responder	7	43.62	25.08	
		18	Non-responder	4	57.51	22.48	
			Responder	6	51.34	26.35	
		24	Non-responder	3	53.06	10.84	
			Responder	9	52.17	25.44	
	NIL	0	Non-responder	2	28.13	7.96	
			Responder	9	33.43	25.64	
			Healthy control	5	14.58	9.83	
		6	Non-responder	4	34.43	11.64	
			Responder	5	19.48	16.49	
		12	Non-responder	1	46.58	.	
			Responder	7	20.39	17.45	
		18	Non-responder	4	18.85	10.66	
			Responder	6	16.14	15.82	
		24	Non-responder	3	21.34	6.82	
			Responder	9	16.71	14.77	
CD14+ CD11c+ HLADR+ (TNFa+) in PBMC	CPE	0	Non-responder	2	0.36	0.44	
			Responder	8	2.63	3.56	
			Healthy control	5	0.71	0.92	
		6	Non-responder	4	0.16	0.15	
			Responder	5	2.38	3.31	
		12	Non-responder	1	0.02	.	
			Responder	7	0.96	2.08	
		18	Non-responder	4	0.08	0.06	
			Responder	6	2.97	3.21	
		24	Non-responder	3	0.06	0.03	
			Responder	9	2.32	3.94	
	LPS	0	Non-responder	2	0.45	0.30	
			Responder	9	3.14	3.11	
			Healthy control	5	1.95	2.59	
		6	Non-responder	4	0.48	0.52	
			Responder	5	2.84	1.78	
		12	Non-responder	1	0.07	.	
			Responder	7	1.55	1.91	
		18	Non-responder	4	0.37	0.44	
			Responder	6	3.67	2.18	
		24	Non-responder	3	0.27	0.20	
			Responder	9	2.60	3.19	
	NIL	0	Non-responder	2	1.01	1.04	
			Responder	9	2.24	2.41	
			Healthy control	5	1.72	3.42	
		6	Non-responder	4	0.36	0.32	
			Responder	5	3.16	3.13	
		12	Non-responder	1	0.23	.	
			Responder	7	1.04	1.42	
		18	Non-responder	4	0.17	0.11	
			Responder	6	2.64	1.99	
		24	Non-responder	3	0.21	0.16	
			Responder	9	2.17	3.31	
CD14+ CD11c+ HLADR+ (TNFa+) in TOTAL CD14 Monocytes	CPE	0	Non-responder	2	18.21	10.56	
			Responder	8	21.21	23.44	
			Healthy control	5	5.35	5.01	
		6	Non-responder	4	9.32	7.18	
			Responder	5	16.29	11.84	
		12	Non-responder	1	22.45	.	
			Responder	7	9.59	20.00	
		18	Non-responder	4	14.83	13.17	
			Responder	6	19.18	14.34	
		24	Non-responder	3	18.55	7.05	
			Responder	9	13.41	20.81	
	LPS	0	Non-responder	2	54.38	0.66	
			Responder	9	52.20	31.23	
			Healthy control	5	26.34	19.86	
		6	Non-responder	4	28.69	24.07	
			Responder	5	56.87	29.21	
		12	Non-responder	1	56.64	.	
			Responder	7	27.50	30.68	
		18	Non-responder	4	41.40	28.27	
			Responder	6	51.89	22.70	
		24	Non-responder	3	56.82	13.32	
			Responder	9	40.46	27.21	
	NIL	0	Non-responder	2	35.89	11.47	
			Responder	9	24.82	21.77	
			Healthy control	5	9.56	11.07	
		6	Non-responder	4	20.06	15.70	
			Responder	5	21.72	13.08	
		12	Non-responder	1	51.83	.	
			Responder	7	12.40	18.18	
		18	Non-responder	4	21.18	15.54	
			Responder	6	24.86	14.57	
		24	Non-responder	3	33.02	8.45	
			Responder	9	14.76	16.34	
CD14- DCs in PBMC	CPE	0	Non-responder	2	93.75	3.23	
			Responder	8	82.81	9.48	
			Healthy control	5	85.01	11.60	
		6	Non-responder	4	93.24	3.28	
			Responder	5	79.63	15.14	
		12	Non-responder	1	98.25	.	
			Responder	7	80.21	15.13	
		18	Non-responder	4	96.77	1.28	
			Responder	6	72.98	3.16	
		24	Non-responder	3	96.93	1.06	
			Responder	9	77.18	11.49	
	LPS	0	Non-responder	2	96.86	0.86	
			Responder	9	89.71	5.49	
			Healthy control	5	91.35	8.38	
		6	Non-responder	4	96.14	1.72	
			Responder	5	91.00	4.50	
		12	Non-responder	1	98.53	.	
			Responder	7	86.98	15.67	
		18	Non-responder	4	96.59	1.89	
			Responder	6	85.10	7.74	
		24	Non-responder	3	96.31	2.98	
			Responder	9	88.51	6.40	
	NIL	0	Non-responder	2	92.72	3.56	
			Responder	9	86.41	6.44	
			Healthy control	5	79.73	31.31	
		6	Non-responder	4	92.84	3.18	
			Responder	5	79.46	14.47	
		12	Non-responder	1	97.18	.	
			Responder	7	81.13	12.94	
		18	Non-responder	4	96.11	1.19	
			Responder	6	78.93	5.77	
		24	Non-responder	3	96.44	1.54	
			Responder	9	78.34	8.95	
CD14- CD11c+ HLADR+ (TOTAL mDCs) in PBMC	CPE	0	Non-responder	2	1.88	1.40	
			Responder	8	4.78	2.34	
			Healthy control	5	2.64	1.04	
		6	Non-responder	4	1.68	0.94	
			Responder	5	4.19	2.31	
		12	Non-responder	1	1.05	.	
			Responder	7	7.78	7.85	
		18	Non-responder	4	0.71	0.16	
			Responder	6	5.73	4.58	
		24	Non-responder	3	0.97	0.86	
			Responder	9	6.54	5.63	
	LPS	0	Non-responder	2	0.96	0.11	
			Responder	9	3.84	2.45	
			Healthy control	5	2.74	1.15	
		6	Non-responder	4	1.12	0.45	
			Responder	5	3.86	2.61	
		12	Non-responder	1	0.85	.	
			Responder	7	5.69	5.81	
		18	Non-responder	4	0.71	0.24	
			Responder	6	4.65	3.12	
		24	Non-responder	3	0.70	0.43	
			Responder	9	5.69	4.73	
	NIL	0	Non-responder	2	1.70	1.17	
			Responder	9	3.28	1.74	
			Healthy control	5	3.49	3.03	
		6	Non-responder	4	1.48	0.79	
			Responder	5	4.62	3.74	
		12	Non-responder	1	0.74	.	
			Responder	7	7.12	7.43	
		18	Non-responder	4	0.78	0.44	
			Responder	6	5.25	4.10	
		24	Non-responder	3	0.75	0.38	
			Responder	9	6.71	5.94	
CD14- CD11c+ HLADR+ (TOTAL mDCs) in CD14-	CPE	0	Non-responder	2	2.03	1.57	
			Responder	8	5.95	3.23	
			Healthy control	5	3.23	1.67	
		6	Non-responder	4	1.82	1.08	
			Responder	5	5.81	4.46	
		12	Non-responder	1	1.07	.	
			Responder	7	9.96	9.61	
		18	Non-responder	4	0.73	0.16	
			Responder	6	7.71	5.79	
		24	Non-responder	3	1.00	0.87	
			Responder	9	8.94	8.00	
	LPS	0	Non-responder	2	0.99	0.10	
			Responder	9	4.35	2.83	
			Healthy control	5	3.03	1.30	
		6	Non-responder	4	1.17	0.49	
			Responder	5	4.32	3.08	
		12	Non-responder	1	0.87	.	
			Responder	7	6.49	6.37	
		18	Non-responder	4	0.73	0.24	
			Responder	6	5.41	3.38	
		24	Non-responder	3	0.72	0.43	
			Responder	9	6.58	5.57	
	NIL	0	Non-responder	2	1.86	1.33	
			Responder	9	3.80	2.02	
			Healthy control	5	9.25	15.57	
		6	Non-responder	4	1.61	0.89	
			Responder	5	6.79	7.43	
		12	Non-responder	1	0.76	.	
			Responder	7	9.06	9.67	
		18	Non-responder	4	0.81	0.45	
			Responder	6	6.58	4.99	
		24	Non-responder	3	0.78	0.38	
			Responder	9	8.90	7.97	
CD14- CD11c+ HLADR+ (CD80+) in PBMC	CPE	0	Non-responder	2	0.57	0.51	
			Responder	8	1.98	1.18	
			Healthy control	5	0.59	0.33	
		6	Non-responder	4	0.30	0.23	
			Responder	5	1.36	1.22	
		12	Non-responder	1	0.10	.	
			Responder	7	3.31	4.62	
		18	Non-responder	4	0.11	0.06	
			Responder	6	2.15	2.74	
		24	Non-responder	3	0.16	0.08	
			Responder	9	2.58	3.23	
	LPS	0	Non-responder	2	0.37	0.20	
			Responder	9	1.94	1.49	
			Healthy control	5	1.01	0.55	
		6	Non-responder	4	0.37	0.34	
			Responder	5	1.88	1.48	
		12	Non-responder	1	0.08	.	
			Responder	7	2.79	3.21	
		18	Non-responder	4	0.18	0.13	
			Responder	6	2.38	1.97	
		24	Non-responder	3	0.17	0.11	
			Responder	9	3.44	3.53	
	NIL	0	Non-responder	2	0.72	0.66	
			Responder	9	1.47	0.99	
			Healthy control	5	0.97	0.68	
		6	Non-responder	4	0.34	0.24	
			Responder	5	1.42	0.71	
		12	Non-responder	1	0.10	.	
			Responder	7	3.82	5.07	
		18	Non-responder	4	0.16	0.15	
			Responder	6	2.16	2.45	
		24	Non-responder	3	0.22	0.12	
			Responder	9	3.23	3.81	
CD14- CD11c+ HLADR+ (CD80+) in TOTAL mDCs	CPE	0	Non-responder	2	27.68	6.68	
			Responder	8	39.43	16.25	
			Healthy control	5	22.77	14.19	
		6	Non-responder	4	15.26	7.60	
			Responder	5	30.69	17.47	
		12	Non-responder	1	9.66	.	
			Responder	7	26.33	26.84	
		18	Non-responder	4	15.93	7.93	
			Responder	6	30.00	13.34	
		24	Non-responder	3	24.65	16.30	
			Responder	9	32.13	21.40	
	LPS	0	Non-responder	2	37.11	16.08	
			Responder	9	45.69	13.41	
			Healthy control	5	34.98	15.34	
		6	Non-responder	4	28.58	16.47	
			Responder	5	45.17	15.66	
		12	Non-responder	1	9.11	.	
			Responder	7	33.78	24.23	
		18	Non-responder	4	28.40	19.27	
			Responder	6	44.92	14.15	
		24	Non-responder	3	31.74	19.86	
			Responder	9	47.79	21.16	
	NIL	0	Non-responder	2	38.09	12.53	
			Responder	9	40.08	18.04	
			Healthy control	5	30.74	13.69	
		6	Non-responder	4	21.98	11.53	
			Responder	5	36.17	17.98	
		12	Non-responder	1	13.15	.	
			Responder	7	32.36	27.32	
		18	Non-responder	4	20.03	13.05	
			Responder	6	35.26	12.95	
		24	Non-responder	3	31.98	17.38	
			Responder	9	36.55	23.93	
CD14- CD11c+ HLADR+ (CD83+) in PBMC	CPE	0	Non-responder	2	0.38	0.21	
			Responder	8	1.25	0.68	
			Healthy control	5	0.49	0.18	
		6	Non-responder	4	0.29	0.11	
			Responder	5	0.89	0.68	
		12	Non-responder	1	0.12	.	
			Responder	7	3.43	5.40	
		18	Non-responder	4	0.13	0.04	
			Responder	6	1.76	2.40	
		24	Non-responder	3	0.19	0.05	
			Responder	9	1.90	3.04	
	LPS	0	Non-responder	2	0.21	0.05	
			Responder	9	1.17	0.94	
			Healthy control	5	0.76	0.37	
		6	Non-responder	4	0.24	0.14	
			Responder	5	1.01	0.65	
		12	Non-responder	1	0.09	.	
			Responder	7	2.32	2.72	
		18	Non-responder	4	0.21	0.06	
			Responder	6	1.34	1.61	
		24	Non-responder	3	0.12	0.01	
			Responder	9	2.26	2.94	
	NIL	0	Non-responder	2	0.41	0.19	
			Responder	9	0.92	0.57	
			Healthy control	5	0.64	0.54	
		6	Non-responder	4	0.31	0.12	
			Responder	5	0.86	0.52	
		12	Non-responder	1	0.14	.	
			Responder	7	3.17	4.60	
		18	Non-responder	4	0.18	0.09	
			Responder	6	1.58	2.08	
		24	Non-responder	3	0.25	0.06	
			Responder	9	2.47	3.81	
CD14- CD11c+ HLADR+ (CD83+) in TOTAL mDCs	CPE	0	Non-responder	2	22.31	5.49	
			Responder	8	30.71	15.87	
			Healthy control	5	20.87	11.82	
		6	Non-responder	4	18.95	4.38	
			Responder	5	26.28	16.38	
		12	Non-responder	1	11.59	.	
			Responder	7	32.83	25.97	
		18	Non-responder	4	19.01	6.66	
			Responder	6	28.92	17.09	
		24	Non-responder	3	30.52	18.34	
			Responder	9	27.43	21.35	
	LPS	0	Non-responder	2	22.25	7.22	
			Responder	9	31.22	9.35	
			Healthy control	5	28.69	9.76	
		6	Non-responder	4	20.43	4.67	
			Responder	5	27.96	9.41	
		12	Non-responder	1	10.46	.	
			Responder	7	35.75	18.67	
		18	Non-responder	4	30.02	3.34	
			Responder	6	24.31	14.99	
		24	Non-responder	3	23.04	16.05	
			Responder	9	34.26	18.09	
	NIL	0	Non-responder	2	26.46	7.18	
			Responder	9	31.56	14.35	
			Healthy control	5	19.29	8.27	
		6	Non-responder	4	22.48	5.99	
			Responder	5	28.00	19.48	
		12	Non-responder	1	19.46	.	
			Responder	7	36.32	21.46	
		18	Non-responder	4	25.66	9.86	
			Responder	6	32.01	20.94	
		24	Non-responder	3	36.91	11.83	
			Responder	9	32.08	23.56	
CD14- CD11c+ HLADR+ (IL4+) in PBMC	CPE	0	Non-responder	2	0.21	0.18	
			Responder	8	1.02	0.71	
			Healthy control	5	0.33	0.18	
		6	Non-responder	4	0.16	0.15	
			Responder	5	0.50	0.52	
		12	Non-responder	1	0.09	.	
			Responder	7	1.65	2.25	
		18	Non-responder	4	0.08	0.04	
			Responder	6	0.77	1.20	
		24	Non-responder	3	0.06	0.04	
			Responder	9	1.21	1.84	
	LPS	0	Non-responder	2	0.21	0.06	
			Responder	9	1.10	1.02	
			Healthy control	5	0.60	0.50	
		6	Non-responder	4	0.18	0.14	
			Responder	5	1.07	1.10	
		12	Non-responder	1	0.07	.	
			Responder	7	1.59	1.92	
		18	Non-responder	4	0.14	0.05	
			Responder	6	1.19	1.34	
		24	Non-responder	3	0.07	0.02	
			Responder	9	1.74	1.96	
	NIL	0	Non-responder	2	0.34	0.31	
			Responder	9	0.73	0.61	
			Healthy control	5	0.33	0.10	
		6	Non-responder	4	0.17	0.08	
			Responder	5	0.54	0.42	
		12	Non-responder	1	0.11	.	
			Responder	7	1.84	2.46	
		18	Non-responder	4	0.10	0.07	
			Responder	6	0.73	1.09	
		24	Non-responder	3	0.07	0.03	
			Responder	9	1.28	1.65	
CD14- CD11c+ HLADR+ (IL4+) in TOTAL mDCs	CPE	0	Non-responder	2	10.52	1.84	
			Responder	8	18.50	11.87	
			Healthy control	5	12.46	6.77	
		6	Non-responder	4	8.77	3.17	
			Responder	5	9.73	9.30	
		12	Non-responder	1	9.01	.	
			Responder	7	13.32	13.15	
		18	Non-responder	4	11.28	3.03	
			Responder	6	8.73	7.81	
		24	Non-responder	3	7.75	2.42	
			Responder	9	12.27	11.81	
	LPS	0	Non-responder	2	21.98	3.51	
			Responder	9	22.88	13.48	
			Healthy control	5	22.45	12.77	
		6	Non-responder	4	14.13	5.30	
			Responder	5	22.37	15.96	
		12	Non-responder	1	7.66	.	
			Responder	7	18.60	14.22	
		18	Non-responder	4	20.48	7.24	
			Responder	6	17.45	17.09	
		24	Non-responder	3	12.95	8.43	
			Responder	9	20.70	15.93	
	NIL	0	Non-responder	2	18.33	5.77	
			Responder	9	17.99	12.15	
			Healthy control	5	13.46	7.77	
		6	Non-responder	4	12.71	4.82	
			Responder	5	13.14	11.91	
		12	Non-responder	1	14.69	.	
			Responder	7	16.06	13.77	
		18	Non-responder	4	12.34	3.86	
			Responder	6	9.73	8.74	
		24	Non-responder	3	9.54	1.94	
			Responder	9	11.58	10.98	
CD14- CD11c+ HLADR+ (IL6+) in PBMC	CPE	0	Non-responder	2	0.15	0.18	
			Responder	8	1.55	0.95	
			Healthy control	5	0.37	0.30	
		6	Non-responder	4	0.08	0.08	
			Responder	5	1.08	1.02	
		12	Non-responder	1	0.05	.	
			Responder	7	3.72	5.62	
		18	Non-responder	4	0.06	0.10	
			Responder	6	1.77	2.60	
		24	Non-responder	3	0.06	0.07	
			Responder	9	2.65	3.64	
	LPS	0	Non-responder	2	0.13	0.03	
			Responder	9	1.59	1.30	
			Healthy control	5	0.96	0.94	
		6	Non-responder	4	0.18	0.11	
			Responder	5	1.71	1.56	
		12	Non-responder	1	0.07	.	
			Responder	7	2.98	3.43	
		18	Non-responder	4	0.19	0.25	
			Responder	6	2.02	2.25	
		24	Non-responder	3	0.06	0.02	
			Responder	9	3.13	3.43	
	NIL	0	Non-responder	2	0.16	0.17	
			Responder	9	1.04	0.83	
			Healthy control	5	0.42	0.17	
		6	Non-responder	4	0.10	0.08	
			Responder	5	1.08	1.09	
		12	Non-responder	1	0.03	.	
			Responder	7	3.46	4.99	
		18	Non-responder	4	0.05	0.06	
			Responder	6	1.72	2.32	
		24	Non-responder	3	0.06	0.07	
			Responder	9	3.12	4.08	
CD14- CD11c+ HLADR+ (IL6+) in TOTAL mDCs	CPE	0	Non-responder	2	6.22	5.03	
			Responder	8	28.96	16.84	
			Healthy control	5	14.38	10.79	
		6	Non-responder	4	4.79	2.84	
			Responder	5	19.63	16.26	
		12	Non-responder	1	4.51	.	
			Responder	7	27.43	30.87	
		18	Non-responder	4	7.22	11.47	
			Responder	6	19.99	18.77	
		24	Non-responder	3	8.91	12.31	
			Responder	9	27.87	24.09	
	LPS	0	Non-responder	2	13.73	1.48	
			Responder	9	35.77	13.38	
			Healthy control	5	36.65	23.77	
		6	Non-responder	4	15.87	6.21	
			Responder	5	38.84	16.13	
		12	Non-responder	1	8.01	.	
			Responder	7	40.15	22.92	
		18	Non-responder	4	23.52	23.62	
			Responder	6	30.50	27.17	
		24	Non-responder	3	11.29	6.31	
			Responder	9	40.78	25.47	
	NIL	0	Non-responder	2	7.87	4.68	
			Responder	9	25.51	15.98	
			Healthy control	5	16.54	10.53	
		6	Non-responder	4	7.15	2.69	
			Responder	5	21.30	16.27	
		12	Non-responder	1	4.65	.	
			Responder	7	27.13	28.01	
		18	Non-responder	4	7.52	9.56	
			Responder	6	21.39	19.38	
		24	Non-responder	3	8.52	10.06	
			Responder	9	28.56	25.47	
CD14- CD11c+ HLADR+ (IL10+) in PBMC	CPE	0	Non-responder	2	0.08	0.08	
			Responder	8	1.26	0.93	
			Healthy control	5	0.30	0.36	
		6	Non-responder	4	0.06	0.06	
			Responder	5	0.57	0.48	
		12	Non-responder	1	0.07	.	
			Responder	7	1.47	2.27	
		18	Non-responder	4	0.02	0.02	
			Responder	6	0.71	0.58	
		24	Non-responder	3	0.03	0.02	
			Responder	9	1.31	1.85	
	LPS	0	Non-responder	2	0.17	0.09	
			Responder	9	1.77	1.48	
			Healthy control	5	0.97	0.99	
		6	Non-responder	4	0.20	0.20	
			Responder	5	1.73	1.75	
		12	Non-responder	1	0.11	.	
			Responder	7	2.54	3.42	
		18	Non-responder	4	0.16	0.10	
			Responder	6	1.69	1.63	
		24	Non-responder	3	0.07	0.03	
			Responder	9	2.36	2.30	
	NIL	0	Non-responder	2	0.12	0.11	
			Responder	9	0.91	0.75	
			Healthy control	5	0.31	0.19	
		6	Non-responder	4	0.11	0.10	
			Responder	5	0.55	0.41	
		12	Non-responder	1	0.03	.	
			Responder	7	2.02	3.08	
		18	Non-responder	4	0.02	0.01	
			Responder	6	0.68	0.64	
		24	Non-responder	3	0.03	0.03	
			Responder	9	1.35	1.78	
CD14- CD11c+ HLADR+ (IL10+) in TOTAL mDCs	CPE	0	Non-responder	2	3.47	1.77	
			Responder	8	22.59	14.87	
			Healthy control	5	9.58	8.37	
		6	Non-responder	4	3.39	1.59	
			Responder	5	10.78	7.65	
		12	Non-responder	1	6.22	.	
			Responder	7	9.89	11.77	
		18	Non-responder	4	2.35	1.81	
			Responder	6	11.58	9.21	
		24	Non-responder	3	3.77	3.90	
			Responder	9	12.32	10.80	
	LPS	0	Non-responder	2	17.45	7.57	
			Responder	9	38.66	19.33	
			Healthy control	5	32.75	22.18	
		6	Non-responder	4	14.76	9.04	
			Responder	5	37.09	16.88	
		12	Non-responder	1	13.04	.	
			Responder	7	26.31	22.04	
		18	Non-responder	4	22.20	8.13	
			Responder	6	28.60	18.86	
		24	Non-responder	3	11.69	3.51	
			Responder	9	32.59	18.51	
	NIL	0	Non-responder	2	6.00	2.50	
			Responder	9	22.14	14.83	
			Healthy control	5	12.33	10.70	
		6	Non-responder	4	6.58	4.44	
			Responder	5	12.99	9.91	
		12	Non-responder	1	3.70	.	
			Responder	7	14.71	16.73	
		18	Non-responder	4	3.27	1.63	
			Responder	6	11.07	7.69	
		24	Non-responder	3	4.20	3.40	
			Responder	9	13.39	11.40	
CD14- CD11c+ HLADR+ (OX40L+) in PBMC	CPE	0	Non-responder	2	0.19	0.01	
			Responder	8	0.98	1.35	
			Healthy control	5	0.27	0.23	
		6	Non-responder	4	0.23	0.08	
			Responder	5	0.25	0.25	
		12	Non-responder	1	0.29	.	
			Responder	7	2.28	4.13	
		18	Non-responder	4	0.17	0.17	
			Responder	6	0.72	1.16	
		24	Non-responder	3	0.21	0.11	
			Responder	9	1.14	2.45	
	LPS	0	Non-responder	2	0.08	0.07	
			Responder	9	0.37	0.32	
			Healthy control	5	0.17	0.08	
		6	Non-responder	4	0.09	0.06	
			Responder	5	0.18	0.09	
		12	Non-responder	1	0.15	.	
			Responder	7	0.99	1.42	
		18	Non-responder	4	0.32	0.29	
			Responder	6	0.28	0.38	
		24	Non-responder	3	0.11	0.05	
			Responder	9	0.75	1.07	
	NIL	0	Non-responder	3	0.10	0.06	
			Responder	11	0.41	0.43	
			Healthy control	5	0.29	0.24	
		6	Non-responder	4	0.10	0.08	
			Responder	8	0.23	0.16	
		12	Non-responder	1	0.04	.	
			Responder	7	1.70	2.78	
		18	Non-responder	4	0.18	0.13	
			Responder	6	0.49	0.78	
		24	Non-responder	3	0.14	0.05	
			Responder	9	1.47	2.92	
CD14- CD11c+ HLADR+ (OX40L+) in TOTAL mDCs	CPE	0	Non-responder	3	12.76	8.55	
			Responder	8	19.32	22.20	
			Healthy control	5	12.70	12.24	
		6	Non-responder	3	12.31	6.24	
			Responder	7	13.68	14.11	
		12	Non-responder	2	13.95	19.73	
			Responder	6	25.32	20.78	
		18	Non-responder	3	14.31	6.65	
			Responder	8	15.10	13.33	
		24	Non-responder	3	26.67	8.21	
			Responder	9	14.65	15.67	
	LPS	0	Non-responder	2	9.00	8.10	
			Responder	9	11.74	7.79	
			Healthy control	5	9.22	8.65	
		6	Non-responder	4	9.05	6.09	
			Responder	5	6.84	5.05	
		12	Non-responder	1	17.39	.	
			Responder	7	14.88	9.61	
		18	Non-responder	4	41.36	28.83	
			Responder	6	6.60	5.44	
		24	Non-responder	3	18.56	8.53	
			Responder	9	12.56	7.97	
	NIL	0	Non-responder	2	7.79	9.42	
			Responder	9	11.03	9.58	
			Healthy control	5	10.40	10.54	
		6	Non-responder	4	7.33	4.75	
			Responder	5	5.86	4.95	
		12	Non-responder	1	5.77	.	
			Responder	7	18.07	14.21	
		18	Non-responder	4	26.52	21.78	
			Responder	6	8.61	6.91	
		24	Non-responder	3	19.25	3.81	
			Responder	9	16.70	17.74	
CD14- CD11c+ HLADR+ (TNFa+) in PBMC	CPE	0	Non-responder	2	0.22	0.25	
			Responder	8	0.08	0.06	
			Healthy control	5	0.11	0.12	
		6	Non-responder	4	0.14	0.11	
			Responder	5	0.04	0.03	
		12	Non-responder	1	0.12	.	
			Responder	7	0.12	0.13	
		18	Non-responder	4	0.10	0.09	
			Responder	6	0.10	0.18	
		24	Non-responder	3	0.08	0.07	
			Responder	9	0.09	0.14	
	LPS	0	Non-responder	2	0.21	0.06	
			Responder	9	0.15	0.09	
			Healthy control	5	0.28	0.17	
		6	Non-responder	4	0.23	0.18	
			Responder	5	0.14	0.09	
		12	Non-responder	1	0.12	.	
			Responder	7	0.31	0.32	
		18	Non-responder	4	0.21	0.14	
			Responder	6	0.11	0.05	
		24	Non-responder	3	0.10	0.05	
			Responder	9	0.24	0.27	
	NIL	0	Non-responder	2	0.25	0.22	
			Responder	9	0.07	0.05	
			Healthy control	5	0.20	0.18	
		6	Non-responder	4	0.15	0.07	
			Responder	5	0.07	0.08	
		12	Non-responder	1	0.08	.	
			Responder	7	0.17	0.20	
		18	Non-responder	4	0.08	0.05	
			Responder	6	0.08	0.12	
		24	Non-responder	3	0.08	0.06	
			Responder	9	0.09	0.14	
CD14- CD11c+ HLADR+ (TNFa+) in TOTAL mDCs	CPE	0	Non-responder	2	9.67	5.87	
			Responder	8	1.91	1.03	
			Healthy control	5	4.59	5.09	
		6	Non-responder	4	7.94	2.24	
			Responder	5	1.49	1.31	
		12	Non-responder	1	11.80	.	
			Responder	7	3.69	4.39	
		18	Non-responder	4	12.60	10.59	
			Responder	6	2.52	4.05	
		24	Non-responder	3	12.09	11.94	
			Responder	9	1.08	0.85	
	LPS	0	Non-responder	2	21.08	3.77	
			Responder	9	5.83	5.10	
			Healthy control	5	13.67	14.51	
		6	Non-responder	4	18.16	6.89	
			Responder	5	5.03	4.32	
		12	Non-responder	1	13.63	.	
			Responder	7	9.64	8.58	
		18	Non-responder	4	27.83	10.78	
			Responder	6	3.43	2.40	
		24	Non-responder	3	18.02	9.74	
			Responder	9	4.54	4.84	
	NIL	0	Non-responder	2	13.69	3.70	
			Responder	9	2.58	2.10	
			Healthy control	5	6.05	5.48	
		6	Non-responder	4	10.61	4.31	
			Responder	5	2.37	2.78	
		12	Non-responder	1	10.23	.	
			Responder	7	5.95	6.41	
		18	Non-responder	4	11.21	6.81	
			Responder	6	2.94	4.88	
		24	Non-responder	3	10.78	8.75	
			Responder	9	1.08	0.90	
CD14- CD11c- HLADR+ in PBMC	CPE	0	Non-responder	2	2.12	2.24	
			Responder	8	3.96	1.37	
			Healthy control	5	2.49	1.23	
		6	Non-responder	4	1.99	1.06	
			Responder	5	3.09	1.40	
		12	Non-responder	1	0.94	.	
			Responder	7	3.29	1.48	
		18	Non-responder	4	1.76	1.90	
			Responder	6	3.59	1.72	
		24	Non-responder	3	1.09	1.13	
			Responder	9	3.66	1.83	
	LPS	0	Non-responder	2	1.21	1.09	
			Responder	9	4.25	2.52	
			Healthy control	5	2.60	0.73	
		6	Non-responder	4	1.85	1.28	
			Responder	5	3.50	1.18	
		12	Non-responder	1	0.76	.	
			Responder	7	4.63	2.90	
		18	Non-responder	4	1.77	2.43	
			Responder	6	3.58	1.19	
		24	Non-responder	3	0.88	0.82	
			Responder	9	4.32	3.11	
	NIL	0	Non-responder	2	2.85	3.40	
			Responder	9	3.30	1.60	
			Healthy control	5	3.45	2.48	
		6	Non-responder	4	2.15	0.86	
			Responder	5	3.34	1.85	
		12	Non-responder	1	0.77	.	
			Responder	7	4.71	2.97	
		18	Non-responder	4	2.32	2.51	
			Responder	6	3.74	2.20	
		24	Non-responder	3	0.84	0.87	
			Responder	9	3.86	2.77	
CD14- CD11c- HLADR+ in CD14-	CPE	0	Non-responder	2	2.30	2.47	
			Responder	8	4.84	1.74	
			Healthy control	5	2.94	1.41	
		6	Non-responder	4	2.15	1.17	
			Responder	5	3.92	1.58	
		12	Non-responder	1	0.96	.	
			Responder	7	4.26	2.01	
		18	Non-responder	4	1.82	1.97	
			Responder	6	4.86	2.15	
		24	Non-responder	3	1.12	1.14	
			Responder	9	4.79	2.48	
	LPS	0	Non-responder	2	1.24	1.12	
			Responder	9	4.73	2.69	
			Healthy control	5	2.84	0.71	
		6	Non-responder	4	1.91	1.30	
			Responder	5	3.84	1.24	
		12	Non-responder	1	0.77	.	
			Responder	7	5.23	2.93	
		18	Non-responder	4	1.82	2.48	
			Responder	6	4.15	1.10	
		24	Non-responder	3	0.91	0.83	
			Responder	9	4.91	3.52	
	NIL	0	Non-responder	2	3.15	3.78	
			Responder	9	3.76	1.62	
			Healthy control	5	8.07	11.96	
		6	Non-responder	4	2.32	0.92	
			Responder	5	4.18	2.18	
		12	Non-responder	1	0.79	.	
			Responder	7	5.89	4.14	
		18	Non-responder	4	2.40	2.59	
			Responder	6	4.70	2.64	
		24	Non-responder	3	0.86	0.89	
			Responder	9	5.03	3.80	
CD14- CD123+ HLADR+ in TOTAL pBMCs	CPE	0	Non-responder	2	0.17	0.09	
			Responder	8	1.07	0.70	
			Healthy control	5	0.31	0.29	
		6	Non-responder	4	0.10	0.04	
			Responder	5	0.68	0.62	
		12	Non-responder	1	0.01	.	
			Responder	7	0.56	0.58	
		18	Non-responder	4	0.06	0.06	
			Responder	6	0.63	0.61	
		24	Non-responder	3	0.04	0.02	
			Responder	9	0.89	0.89	
	LPS	0	Non-responder	2	0.26	0.16	
			Responder	9	1.44	1.15	
			Healthy control	5	0.54	0.40	
		6	Non-responder	4	0.18	0.06	
			Responder	5	1.06	0.68	
		12	Non-responder	1	0.03	.	
			Responder	7	1.10	1.14	
		18	Non-responder	4	0.09	0.05	
			Responder	6	0.82	0.59	
		24	Non-responder	3	0.05	0.03	
			Responder	9	1.43	1.24	
	NIL	0	Non-responder	2	0.19	0.15	
			Responder	9	0.88	0.74	
			Healthy control	5	0.35	0.33	
		6	Non-responder	4	0.11	0.05	
			Responder	5	0.59	0.46	
		12	Non-responder	1	0.02	.	
			Responder	7	0.96	1.22	
		18	Non-responder	4	0.05	0.02	
			Responder	6	0.59	0.63	
		24	Non-responder	3	0.03	0.01	
			Responder	9	1.05	1.16	
CD14- CD123+ HLADR+ in (TOTAL pDCs) in CD14-	CPE	0	Non-responder	2	0.18	0.10	
			Responder	8	1.28	0.80	
			Healthy control	5	0.36	0.30	
		6	Non-responder	4	0.11	0.04	
			Responder	5	0.84	0.67	
		12	Non-responder	1	0.01	.	
			Responder	7	0.70	0.73	
		18	Non-responder	4	0.06	0.06	
			Responder	6	0.86	0.83	
		24	Non-responder	3	0.04	0.02	
			Responder	9	1.17	1.22	
	LPS	0	Non-responder	2	0.26	0.16	
			Responder	9	1.61	1.24	
			Healthy control	5	0.60	0.41	
		6	Non-responder	4	0.19	0.06	
			Responder	5	1.18	0.77	
		12	Non-responder	1	0.03	.	
			Responder	7	1.21	1.24	
		18	Non-responder	4	0.09	0.05	
			Responder	6	0.97	0.73	
		24	Non-responder	3	0.05	0.03	
			Responder	9	1.64	1.43	
	NIL	0	Non-responder	2	0.21	0.17	
			Responder	9	0.99	0.78	
			Healthy control	5	0.55	0.49	
		6	Non-responder	4	0.12	0.06	
			Responder	5	0.71	0.46	
		12	Non-responder	1	0.02	.	
			Responder	7	1.24	1.69	
		18	Non-responder	4	0.05	0.02	
			Responder	6	0.73	0.76	
		24	Non-responder	3	0.03	0.01	
			Responder	9	1.38	1.58	
CD14- CD123+ HLADR+ (CD80+) in PBMC	CPE	0	Non-responder	2	0.03	0.03	
			Responder	8	0.35	0.32	
			Healthy control	5	0.10	0.11	
		6	Non-responder	4	0.03	0.01	
			Responder	5	0.17	0.24	
		12	Non-responder	1	0.00	.	
			Responder	7	0.13	0.10	
		18	Non-responder	4	0.03	0.05	
			Responder	6	0.13	0.10	
		24	Non-responder	3	0.01	0.01	
			Responder	9	0.24	0.22	
	LPS	0	Non-responder	2	0.10	0.09	
			Responder	9	0.49	0.34	
			Healthy control	5	0.20	0.15	
		6	Non-responder	4	0.07	0.04	
			Responder	5	0.35	0.23	
		12	Non-responder	1	0.01	.	
			Responder	7	0.41	0.43	
		18	Non-responder	4	0.03	0.02	
			Responder	6	0.31	0.22	
		24	Non-responder	3	0.02	0.01	
			Responder	9	0.51	0.43	
	NIL	0	Non-responder	2	0.05	0.06	
			Responder	9	0.25	0.23	
			Healthy control	5	0.14	0.14	
		6	Non-responder	4	0.04	0.04	
			Responder	5	0.16	0.19	
		12	Non-responder	1	0.00	.	
			Responder	7	0.19	0.19	
		18	Non-responder	4	0.02	0.01	
			Responder	6	0.15	0.13	
		24	Non-responder	3	0.01	0.01	
			Responder	9	0.25	0.25	
CD14- CD123+ HLADR+ (CD80+) in TOTAL pDCs	CPE	0	Non-responder	2	17.08	10.74	
			Responder	8	27.96	10.33	
			Healthy control	5	29.45	10.67	
		6	Non-responder	4	27.05	7.70	
			Responder	5	22.13	13.14	
		12	Non-responder	1	16.67	.	
			Responder	7	29.99	19.07	
		18	Non-responder	4	41.57	18.35	
			Responder	6	27.41	15.40	
		24	Non-responder	3	32.38	13.19	
			Responder	9	33.64	15.19	
	LPS	0	Non-responder	2	36.84	10.83	
			Responder	9	37.45	10.51	
			Healthy control	5	37.21	4.26	
		6	Non-responder	4	38.16	11.69	
			Responder	5	34.09	7.94	
		12	Non-responder	1	32.62	.	
			Responder	7	36.08	13.43	
		18	Non-responder	4	37.58	8.64	
			Responder	6	40.06	13.77	
		24	Non-responder	3	39.41	4.34	
			Responder	9	41.86	13.29	
	NIL	0	Non-responder	2	23.10	11.62	
			Responder	9	30.82	18.29	
			Healthy control	5	38.13	17.43	
		6	Non-responder	4	31.63	18.38	
			Responder	5	24.02	11.95	
		12	Non-responder	1	24.78	.	
			Responder	7	29.85	18.20	
		18	Non-responder	4	38.14	5.23	
			Responder	6	30.93	14.99	
		24	Non-responder	3	34.27	19.22	
			Responder	9	26.99	9.14	
CD14- CD123+ HLADR+ (CD83+) in PBMC	CPE	0	Non-responder	2	0.05	0.03	
			Responder	8	0.20	0.16	
			Healthy control	5	0.04	0.04	
		6	Non-responder	4	0.02	0.01	
			Responder	5	0.12	0.11	
		12	Non-responder	1	0.00	.	
			Responder	7	0.13	0.15	
		18	Non-responder	4	0.01	0.01	
			Responder	6	0.13	0.12	
		24	Non-responder	3	0.01	0.00	
			Responder	9	0.23	0.35	
	LPS	0	Non-responder	2	0.14	0.08	
			Responder	9	0.41	0.39	
			Healthy control	5	0.13	0.07	
		6	Non-responder	4	0.06	0.02	
			Responder	5	0.39	0.40	
		12	Non-responder	1	0.01	.	
			Responder	7	0.40	0.50	
		18	Non-responder	4	0.04	0.02	
			Responder	6	0.30	0.32	
		24	Non-responder	3	0.02	0.01	
			Responder	9	0.54	0.60	
	NIL	0	Non-responder	2	0.05	0.03	
			Responder	9	0.14	0.17	
			Healthy control	5	0.03	0.02	
		6	Non-responder	4	0.02	0.01	
			Responder	5	0.10	0.08	
		12	Non-responder	1	0.01	.	
			Responder	7	0.15	0.17	
		18	Non-responder	4	0.01	0.00	
			Responder	6	0.10	0.08	
		24	Non-responder	3	0.01	0.00	
			Responder	9	0.19	0.30	
CD14- CD123+ HLADR+ (CD83+) in TOTAL pDCs	CPE	0	Non-responder	2	29.81	2.63	
			Responder	8	22.25	15.06	
			Healthy control	5	16.38	12.57	
		6	Non-responder	4	25.11	8.00	
			Responder	5	22.06	10.04	
		12	Non-responder	1	16.67	.	
			Responder	7	19.83	10.10	
		18	Non-responder	4	23.57	11.60	
			Responder	6	22.69	9.70	
		24	Non-responder	3	29.73	11.44	
			Responder	9	21.17	13.35	
	LPS	0	Non-responder	2	52.92	0.81	
			Responder	9	33.87	22.09	
			Healthy control	5	30.27	15.70	
		6	Non-responder	4	33.88	12.55	
			Responder	5	33.48	19.71	
		12	Non-responder	1	31.21	.	
			Responder	7	30.26	16.65	
		18	Non-responder	4	41.73	6.52	
			Responder	6	31.49	19.04	
		24	Non-responder	3	38.55	9.30	
			Responder	9	33.57	20.82	
	NIL	0	Non-responder	2	30.86	11.17	
			Responder	9	18.46	12.94	
			Healthy control	5	13.36	11.74	
		6	Non-responder	4	21.19	10.08	
			Responder	5	18.87	7.95	
		12	Non-responder	1	29.20	.	
			Responder	7	18.01	10.75	
		18	Non-responder	4	24.67	10.82	
			Responder	6	19.98	10.30	
		24	Non-responder	3	27.73	4.47	
			Responder	9	21.80	15.30	
CD14- CD123+ HLADR+ (IL4+) in PBMC	CPE	0	Non-responder	2	0.01	0.01	
			Responder	8	0.15	0.16	
			Healthy control	5	0.03	0.03	
		6	Non-responder	4	0.01	0.00	
			Responder	5	0.08	0.09	
		12	Non-responder	1	0.00	.	
			Responder	7	0.08	0.11	
		18	Non-responder	4	0.01	0.00	
			Responder	6	0.07	0.08	
		24	Non-responder	3	0.00	0.00	
			Responder	9	0.21	0.38	
	LPS	0	Non-responder	2	0.08	0.05	
			Responder	9	0.33	0.34	
			Healthy control	5	0.12	0.08	
		6	Non-responder	4	0.03	0.02	
			Responder	5	0.33	0.36	
		12	Non-responder	1	0.01	.	
			Responder	7	0.28	0.41	
		18	Non-responder	4	0.01	0.00	
			Responder	6	0.25	0.27	
		24	Non-responder	3	0.01	0.00	
			Responder	9	0.42	0.46	
	NIL	0	Non-responder	2	0.03	0.02	
			Responder	9	0.12	0.14	
			Healthy control	5	0.03	0.02	
		6	Non-responder	4	0.01	0.01	
			Responder	5	0.06	0.05	
		12	Non-responder	1	0.00	.	
			Responder	7	0.11	0.15	
		18	Non-responder	4	0.00	0.00	
			Responder	6	0.06	0.05	
		24	Non-responder	3	0.00	0.00	
			Responder	9	0.21	0.35	
CD14- CD123+ HLADR+ (IL4+) in TOTAL pDCs	CPE	0	Non-responder	2	7.00	3.31	
			Responder	8	14.24	12.36	
			Healthy control	5	11.43	10.16	
		6	Non-responder	4	7.82	2.24	
			Responder	5	12.24	10.91	
		12	Non-responder	1	0.00	.	
			Responder	7	9.97	10.76	
		18	Non-responder	4	12.77	6.73	
			Responder	6	10.54	5.72	
		24	Non-responder	3	6.88	3.59	
			Responder	9	14.25	13.81	
	LPS	0	Non-responder	2	28.10	4.38	
			Responder	9	25.12	21.15	
			Healthy control	5	24.96	14.39	
		6	Non-responder	4	16.80	7.55	
			Responder	5	26.32	18.68	
		12	Non-responder	1	21.99	.	
			Responder	7	17.16	17.13	
		18	Non-responder	4	20.43	8.21	
			Responder	6	22.78	18.06	
		24	Non-responder	3	15.47	3.89	
			Responder	9	23.29	20.26	
	NIL	0	Non-responder	2	14.85	0.04	
			Responder	9	14.87	11.76	
			Healthy control	5	11.64	6.11	
		6	Non-responder	4	12.98	8.49	
			Responder	5	11.28	6.73	
		12	Non-responder	1	23.01	.	
			Responder	7	9.64	8.03	
		18	Non-responder	4	9.00	2.30	
			Responder	6	11.18	7.87	
		24	Non-responder	3	5.42	3.02	
			Responder	9	14.79	13.51	
CD14- CD123+ HLADR+ (IL6+) in PBMC	CPE	0	Non-responder	2	0.01	0.01	
			Responder	8	0.08	0.07	
			Healthy control	5	0.03	0.03	
		6	Non-responder	4	0.01	0.00	
			Responder	5	0.05	0.04	
		12	Non-responder	1	0.00	.	
			Responder	7	0.08	0.09	
		18	Non-responder	4	0.01	0.01	
			Responder	6	0.06	0.05	
		24	Non-responder	3	0.00	0.00	
			Responder	9	0.17	0.28	
	LPS	0	Non-responder	2	0.04	0.04	
			Responder	9	0.28	0.34	
			Healthy control	5	0.11	0.09	
		6	Non-responder	4	0.03	0.02	
			Responder	5	0.29	0.30	
		12	Non-responder	1	0.00	.	
			Responder	7	0.39	0.50	
		18	Non-responder	4	0.02	0.03	
			Responder	6	0.22	0.23	
		24	Non-responder	3	0.01	0.01	
			Responder	9	0.50	0.48	
	NIL	0	Non-responder	2	0.02	0.02	
			Responder	9	0.07	0.11	
			Healthy control	5	0.02	0.02	
		6	Non-responder	4	0.01	0.01	
			Responder	5	0.03	0.03	
		12	Non-responder	1	0.00	.	
			Responder	7	0.13	0.18	
		18	Non-responder	4	0.01	0.01	
			Responder	6	0.05	0.05	
		24	Non-responder	3	0.00	0.00	
			Responder	9	0.18	0.26	
CD14- CD123+ HLADR+ (IL6+) in TOTAL pDCs	CPE	0	Non-responder	2	6.33	3.78	
			Responder	8	8.58	5.99	
			Healthy control	5	9.00	9.08	
		6	Non-responder	4	6.18	2.64	
			Responder	5	6.07	4.11	
		12	Non-responder	1	0.00	.	
			Responder	7	12.38	7.01	
		18	Non-responder	4	8.08	8.19	
			Responder	6	9.39	5.53	
		24	Non-responder	3	12.61	15.19	
			Responder	9	12.44	9.79	
	LPS	0	Non-responder	2	16.05	4.23	
			Responder	9	20.47	19.54	
			Healthy control	5	24.66	21.30	
		6	Non-responder	4	14.19	3.93	
			Responder	5	23.35	14.75	
		12	Non-responder	1	9.22	.	
			Responder	7	27.64	18.47	
		18	Non-responder	4	21.21	19.91	
			Responder	6	20.72	14.63	
		24	Non-responder	3	15.93	4.75	
			Responder	9	27.28	19.95	
	NIL	0	Non-responder	2	7.57	2.88	
			Responder	9	8.22	9.39	
			Healthy control	5	6.56	4.98	
		6	Non-responder	4	9.78	4.52	
			Responder	5	5.97	3.77	
		12	Non-responder	1	8.85	.	
			Responder	7	12.55	9.06	
		18	Non-responder	4	8.72	9.94	
			Responder	6	8.88	5.63	
		24	Non-responder	3	9.51	6.78	
			Responder	9	11.77	9.35	
CD14- CD123+ HLADR+ (IL10+) in PBMC	CPE	0	Non-responder	2	0.01	0.00	
			Responder	8	0.08	0.08	
			Healthy control	5	0.02	0.02	
		6	Non-responder	4	0.00	0.00	
			Responder	5	0.04	0.06	
		12	Non-responder	1	0.00	.	
			Responder	7	0.04	0.05	
		18	Non-responder	4	0.01	0.01	
			Responder	6	0.03	0.03	
		24	Non-responder	3	0.00	0.00	
			Responder	9	0.06	0.08	
	LPS	0	Non-responder	2	0.02	0.02	
			Responder	9	0.21	0.17	
			Healthy control	5	0.07	0.06	
		6	Non-responder	4	0.01	0.01	
			Responder	5	0.13	0.11	
		12	Non-responder	1	0.00	.	
			Responder	7	0.27	0.39	
		18	Non-responder	4	0.02	0.01	
			Responder	6	0.10	0.08	
		24	Non-responder	3	0.01	0.00	
			Responder	9	0.21	0.21	
	NIL	0	Non-responder	2	0.01	0.00	
			Responder	9	0.06	0.06	
			Healthy control	5	0.02	0.03	
		6	Non-responder	4	0.01	0.01	
			Responder	5	0.03	0.04	
		12	Non-responder	1	0.00	.	
			Responder	7	0.08	0.13	
		18	Non-responder	4	0.00	0.00	
			Responder	6	0.03	0.04	
		24	Non-responder	3	0.00	0.00	
			Responder	9	0.07	0.10	
CD14- CD123+ HLADR+ (IL10+) in TOTAL pDCs	CPE	0	Non-responder	2	4.58	0.12	
			Responder	8	5.30	3.58	
			Healthy control	5	4.60	3.41	
		6	Non-responder	4	4.54	3.52	
			Responder	5	3.12	3.52	
		12	Non-responder	1	0.00	.	
			Responder	7	6.32	5.20	
		18	Non-responder	4	7.47	6.08	
			Responder	6	4.59	3.90	
		24	Non-responder	3	7.80	5.11	
			Responder	9	4.74	3.58	
	LPS	0	Non-responder	2	7.41	2.11	
			Responder	9	12.49	8.16	
			Healthy control	5	12.49	5.19	
		6	Non-responder	4	7.16	3.37	
			Responder	5	10.35	4.91	
		12	Non-responder	1	9.22	.	
			Responder	7	15.44	11.53	
		18	Non-responder	4	14.89	5.54	
			Responder	6	10.41	4.28	
		24	Non-responder	3	10.05	3.13	
			Responder	9	11.90	6.27	
	NIL	0	Non-responder	2	3.71	1.11	
			Responder	9	4.51	3.18	
			Healthy control	5	4.38	3.42	
		6	Non-responder	4	5.61	3.10	
			Responder	5	3.42	3.37	
		12	Non-responder	1	4.42	.	
			Responder	7	6.42	4.24	
		18	Non-responder	4	7.47	4.49	
			Responder	6	4.39	2.93	
		24	Non-responder	3	9.23	4.16	
			Responder	9	4.57	4.22	
CD14- CD123+ HLADR+ (TNFa+) in PBMC	CPE	0	Non-responder	2	0.01	0.01	
			Responder	8	0.07	0.10	
			Healthy control	5	0.01	0.01	
		6	Non-responder	4	0.01	0.00	
			Responder	5	0.01	0.01	
		12	Non-responder	1	0.00	.	
			Responder	7	0.05	0.08	
		18	Non-responder	4	0.00	0.00	
			Responder	6	0.02	0.03	
		24	Non-responder	3	0.00	0.00	
			Responder	9	0.12	0.20	
	LPS	0	Non-responder	2	0.03	0.01	
			Responder	9	0.12	0.12	
			Healthy control	5	0.04	0.03	
		6	Non-responder	4	0.02	0.01	
			Responder	5	0.07	0.06	
		12	Non-responder	1	0.01	.	
			Responder	7	0.30	0.53	
		18	Non-responder	4	0.01	0.01	
			Responder	6	0.10	0.11	
		24	Non-responder	3	0.01	0.00	
			Responder	9	0.26	0.32	
	NIL	0	Non-responder	2	0.02	0.01	
			Responder	9	0.07	0.10	
			Healthy control	5	0.01	0.01	
		6	Non-responder	4	0.01	0.01	
			Responder	5	0.02	0.02	
		12	Non-responder	1	0.00	.	
			Responder	7	0.13	0.26	
		18	Non-responder	4	0.00	0.00	
			Responder	6	0.02	0.03	
		24	Non-responder	3	0.00	0.00	
			Responder	9	0.12	0.16	
CD14- CD123+ HLADR+ (TNFa+) in TOTAL pDCs	CPE	0	Non-responder	2	7.08	2.25	
			Responder	8	5.78	6.53	
			Healthy control	5	2.66	2.01	
		6	Non-responder	4	6.89	4.13	
			Responder	5	2.05	0.87	
		12	Non-responder	1	0.00	.	
			Responder	7	5.98	8.68	
		18	Non-responder	4	4.73	2.96	
			Responder	6	3.22	3.64	
		24	Non-responder	3	8.30	7.81	
			Responder	9	6.88	7.72	
	LPS	0	Non-responder	3	15.04	3.52	
			Responder	9	7.83	4.40	
			Healthy control	5	7.40	3.57	
		6	Non-responder	4	10.65	5.81	
			Responder	5	6.38	2.09	
		12	Non-responder	1	21.28	.	
			Responder	7	14.47	15.59	
		18	Non-responder	4	14.34	4.39	
			Responder	6	9.23	7.29	
		24	Non-responder	3	14.21	3.25	
			Responder	9	11.15	9.29	
	NIL	0	Non-responder	2	11.57	2.48	
			Responder	9	6.14	7.10	
			Healthy control	5	10.15	13.20	
		6	Non-responder	4	11.31	12.40	
			Responder	5	2.42	1.94	
		12	Non-responder	1	15.93	.	
			Responder	7	6.95	6.68	
		18	Non-responder	4	4.65	2.29	
			Responder	6	4.10	4.85	
		24	Non-responder	3	5.24	2.42	
			Responder	9	5.74	6.11	
